# Synthesis, Characterisation and *In Vitro* Permeation, Dissolution and Cytotoxic Evaluation of Ruthenium(II)-Liganded Sulpiride and Amino Alcohol

**DOI:** 10.1038/s41598-019-40538-1

**Published:** 2019-03-11

**Authors:** Gretta C. M’bitsi-Ibouily, Thashree Marimuthu, Pradeep Kumar, Yahya E. Choonara, Lisa C. du Toit, Priyamvada Pradeep, Girish Modi, Viness Pillay

**Affiliations:** 1 Wits Advanced Drug Delivery Platform Research Unit, Department of Pharmacy and Pharmacology, School of Therapeutic Sciences, Faculty of Health Sciences, University of the Witwatersrand, Johannesburg, 7 York Road, Parktown, 2193 South Africa; 2 Department of Neurology, Division of Neurosciences, Faculty of Health Sciences, University of the Witwatersrand, Johannesburg, 7 York Road, Parktown, 2193 South Africa

## Abstract

Sulpiride (SPR) is a selective antagonist of central dopamine receptors but has limited clinical use due to its poor pharmacokinetics. The aim of this study was to investigate how metal ligation to SPR may improve its solubility, intestinal permeability and prolong its half-life. The synthesis and characterisation of ternary metal complexes [Ru(*p* -cymene)(L)(SPR)]PF_6_ (L1 = (R)-(+)-2-amino-3-phenyl-1-propanol, L2 = ethanolamine, L3 = (S)-(+)-2-amino-1-propanol, L4 = 3-amino-1-propanol, L5 = (S)-(+)-2-pyrrolidinemethanol) are described in this work. The stability constant of the [Ru(*p* -cymene)(SPR)] complex was determined using Job’s method. The obtained value revealed higher stability of the metal complex in the physiological pH than in an acidic environment such as the stomach. The ternary metal complexes were characterised by elemental analysis, Fourier transform infrared spectroscopy (FT-IR), ^1^H and ^13^C nuclear magnetic resonance (NMR), differential scanning calorimetry (DSC), thermal analyses, Ultraviolet-Visible (UV-Vis). Solubility studies showed higher aqueous solubility for complexed SPR than the free drug. Dissolution profiles of SPR from the metal complexes exhibited slower dissolution rate of the drug. Permeation studies through the pig’s intestine revealed enhanced membrane permeation of the complexed drug. *In vitro* methyl thiazolyl tetrazolium (MTT) assay showed no noticeable toxic effects of the ternary metal complexes on Caco-2 cell line.

## Introduction

Many drug candidates that reach the clinical trial phase are unsuccessful due to several limitations, including poor pharmacokinetic properties. Such shortcomings also affect a considerable number of therapeutic agents already on the market^1–3^. A possible solution to these problems is the design of drug delivery systems with the ability to overcome these limitations, thus pharmaceutical scientists are exploring various drug delivery strategies^4^. Such strategies include the intentional, reversible modification of the physiochemical characteristics of a pharmaceutical in clinical use through the formation of a coordination complex with a transition metal^5,6^. The rapid advances of coordination complexes, also known as metal complexes, as functional materials (catalysts, magnetic and porous materials) have motivated scientists in the pharmaceuticals and medicinal chemistry fields to focus on the research of metal complexes to investigate their potential in medical applications^7–9^. Coordination complexes using the metal as a drug carrier have subsequently shown their usefulness as both diagnostic and therapeutic agents^10,11^. Metal carriers are a simple drug delivery strategy with the ability to induce pharmacokinetic changes to improve aqueous and lipid solubility, bioavailability, permeation and to achieve controlled drug release, while avoiding the time-consuming and costly drug discovery process^12,13^. A recent study showed pharmacokinetic improvement of the standard drug for the treatment of hypothyroidism through metal coordination. In fact, a zinc-coordinated form of the drug was synthesized and formulated into coated gelatin capsules, which were orally administered to rats to achieve sustained drug release. The metal-coordinated drug exhibited slower absorption and prolonged bioavailability over time, compared to the free drug. It was demonstrated that both the slower rate of drug absorption and its sustained release were the result of a mechanism by which the drug’s molecules separate from the metal complex by exchange with endogenous ligands before absorption into the bloodstream. The ligand exchange rate contribute to the slower rate of drug’s delivery (extended drug release) as well as the extended period of drug’s absorption^14^. The use of different metals and ligands in metal coordination affects a range of pharmacokinetic changes; hence metal coordination compounds could enhance the properties of known medicinal drugs^15–17^.

Aqueous solubility improvement of a drug through metal complexation has been previously demonstrated by Ross and Riley who observed an increase in the aqueous solubility of lomefloxacin in the presence of calcium, magnesium, aluminium and iron ions^18^. Shaikh and co-workers later synthesised a bismuth(III)-norfloxacin complex and investigated its pH-solubility profile. Up to pH 6.5.,the complexed drug showed higher aqueous solubility than the free drug, after which the solubility of the drug in the metal complex declined while that of the free drug remained unchanged^19^. A further study in this area was conducted by Breda and co-workers on aluminium (III) complexes of ciprofloxacin and norfloxacin. Bioactives in both complexes exhibited higher aqueous solubility than the respective free drugs in the pH range 2–9^20^.

The manipulation of lipophilicity can be used as a drug delivery strategy to promote the enhanced diffusion of a drug through lipid membranes such as the blood-brain-barrier (BBB) and the intestinal membrane^21,22^. Pinto and co-workers have previously demonstrated that ferrocene–encephalin, an example of a organometallic linker conjugated to a neuropeptide, resulted in increased BBB penetration of [Leu5]-enkephalin^23^. Metal complexation could therefore be used to enhance the lipid solubility and thus the permeation of drugs through the lipid membranes.

The presence of bonds highly responsive to their environment within transition metals allows them to exhibit controlled drug release. Stimuli-responsive complexes can therefore be designed that are inert under normal physiological conditions but become labile with a change in environment such as redox status, pH or the localised application of light. The voluntary deactivation of the bioactive through metal complexation can reduce the incidence of undesirable effects^5^. Ruthenium metal complexes have shown great potential for use as therapeutic compounds^24^. Such metal complexes have many benefits, including improved water solubility, thus improved bioavailability, and increased lipophilicity for better absorption through the cell membrane^25^. In addition to that, the metal complexing strategy results in relatively long half-lives, which allow for fewer administrations of the drug^26^. Furthermore, ruthenium complexes can also be used as inert structures with extended valance space available for additional auxiliary ligands and drugs^27,28^ and this can enable the design and application of Ru metal complexes as potential drug carriers.

Sulpiride (SPR) is a substituted benzamide derivative antipsychotic agent that belongs to class IV in the Biopharmaceutical Classification System (BCS), and thus has poor aqueous solubility and limited intestinal permeability^29,30^. This drug is therefore slowly and poorly absorbed in the gastrointestinal tract after oral administration, resulting in a bioavailability of approximately 30%, coupled with a relatively short half-life of 6 to 8 hours^31^. SPR also has a high burst release effect and poorly penetrates the intestinal membrane^22^. These properties of SPR result in patients needing high doses of the drug to be treated, with a frequent dosing schedule such as a 400 mg tablet to be taken three times a day to reach a maximum daily dosage of 1200 mg^32^. The challenge is that high doses of SPR negatively affect patient compliance and result in undesirable side effects^29,32^. Despite all these limitations, SPR remains an effective antipsychotic, thus the need to develop strategies to improve its pharmacokinetics^33,34^.

This study focuses on the investigation of ruthenium (Ru) metal as a possible drug carrier with the ability to enhance the intestinal permeability and to retard the release of the drug bonded to it. The antipsychotic drug sulpiride is a good candidate for this work due to its low intestinal permeability and burst drug release. The following five amino alcohols are used as ancillary ligands to synthesise ternary metal complexes of sulpiride: L1 = (R)-(+)-2-amino-3-phenyl-1-propanol, L2 = ethanolamine, L3 = (S)-(+)-2-amino-1-propanol, L4 = 3-amino-1-propanol, L5 = (S)-(+)-2-pyrrolidinemethanol. These molecules help stabilise the complex without directly interfering with its chemistry. Different ones are used to investigate their possible effect on the properties of the [Ru(II) – SPR] complex. The determination of the stability constant of the [Ru(II) – SPR] complex, the synthesis and characterisation of five ternary ruthenium (II) complexes with general formula [Ru(*p* -cymene)(L)(SPR)]PF_6_, as well as, solubility, dissolution, permeation and cytotoxicity studies of the drug incorporated in the metal complexes are reported.

## Results and Discussion

### Formation/Dissociation constant of the complex [Ru(p-cymene)Cl(SPR)]

**Figure 1:**
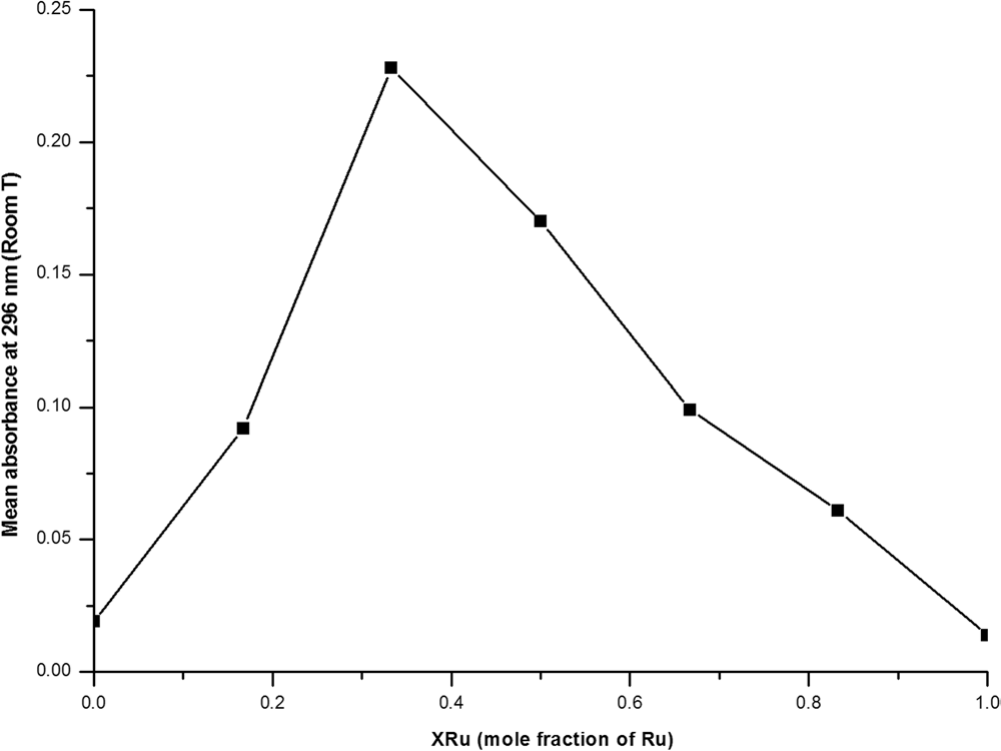
Job’s curve for the formation constant of equimolar solutions of SPR and Ru (II).

The metal to ligand ratio and the stability constant of complex [Ru(*p*-cymene)Cl(SPR)] were determined using the continuous variation method, also known as Job’s method. Experimental data of absorbance at room temperature are shown in Table 1 and the Job’s curve in Fig. 1 shows a maximum absorbance at a mole ratio X_Ru_ = 0.33, indicating the formation of a complex having 1:2 metal to ligand ratio. In Fig. 1, the extrapolated value at the point of cross section on the Job’s curve corresponds to the total absorbance of the complex, if the complex formation had been completed. Since the complex is dissociative in nature, the actual absorbance is somewhat lower than the absorbance measured at break point.

**Table 1:** Experimental data of ruthenium(II)-sulpiride complex by continuous variation method.

Sr. No.	Metal concentration (x10^−4^ M)	Ligand concentration (x10^−4^ M)	XRu (mole fraction of Ru)	Mean absorbance at 288 nm (Room T)
1	0	12	0	0,019
2	2	10	0,167	0,092
3	4	8	0,333	0,228
4	6	6	0,5	0,170
5	8	4	0,667	0,099
6	10	2	0,833	0,061
7	12	0	1	0,014

From the experimental data (Table 1) and the Job’s curve (Fig. 1), A1, A2, C(Ru) and C(SPR) were obtained and used in Eqs (1) and (2) to calculate the complex’s formation and dissociation constants. The formation constant of the complex (log K = 5.45) is between 3 and 6, indicating that the Ru(II)-SPR complex is likely to dissociate in acidic environment, such as the stomach. In the physiological pH of 7.4, however, this complex is expected to be more stable^35^.

### Infrared (IR) spectra with assignments of L1–5, complexes 1–5 and complexes 1a–5a

**Figure 2:**
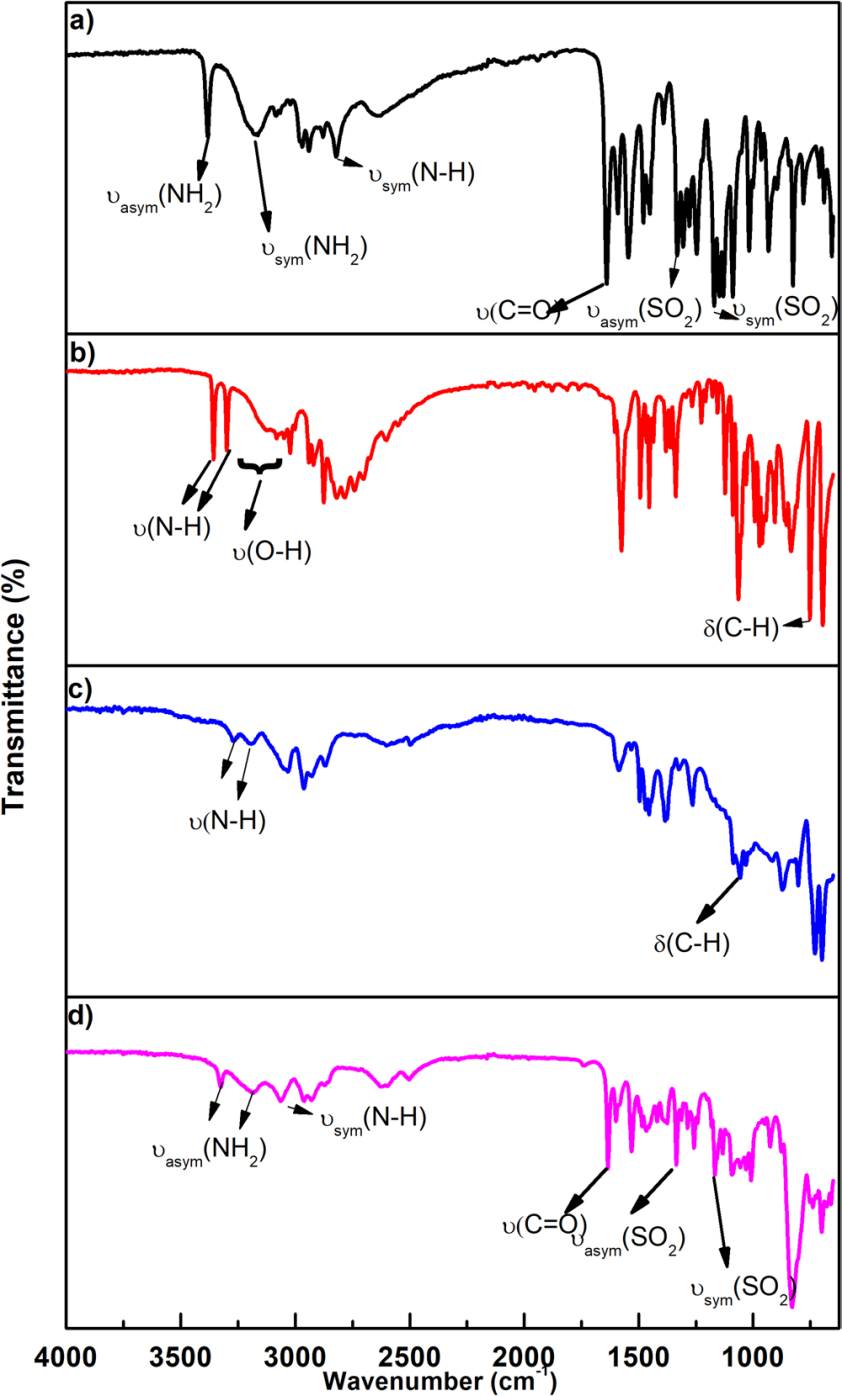
FTIR spectra of (**a**) Sulpiride (SPR), (**b**) (R)-(+)-2-amino-3-phenyl-1-propanol (L1), (**c**) [Ru(p-cymene)((R)-(+)-2-amino-3-phenyl-1-propanol)] (Complex **1**) and (**d**) [Ru(p-cymene)((R)-(+)-2-amino-3-phenyl-1-propanol)(sulpiride)]PF_6_ (Complex **1a**).

The IR spectra of the free amino alcohols L1–5 were compared with the IR spectra of the precursor complexes **1**–**5** to identify the site of coordination involved in the chelation process. The IR spectra of complexes **1–5** were similar. Figure 2b,c show the IR spectra of L1 and complex **1**. The observed absorption bands in the IR spectra of the amino alcohol ligands at wavenumbers in the range 3200–3600 cm^−1^ (Fig. 2b) confirm the presence of primary OH- groups within their structure, as observed in the literature^36^. The disappearance of the OH stretching band in the IR spectra of complexes **1–5** (Fig. 2c) was indicative of Ru-O bond formation, as reported by Wang and co-workers^36^. Upon complexation to the metal, some shifts in absorption bands of the ligands were observed. For instance, L1 has a structure with a monosubstituted aromatic ring which absorption’s band shifted from 696 cm^−1^ in L1 to 872 cm^−1^ in complex **1**. There was also a shift in the NH bending bands from 1575 and 753 cm^−1^ in L1 to 1585 and 730 cm^−1^ in complex **1** (Fig. 2b,c). These shifts in the IR spectra of complexes **1–5** confirm that metal complexation occurred.

The IR spectrum of the free SPR was compared to the IR spectra of complexes 1a–5a to assess coordination of SPR to the metal (Table 2). The IR spectra of SPR and complex **1a** are shown in Fig. 2a,d, indicating a few differences between the two spectra. The ʋ_sym_ (SO_2_) and the ʋ_asym_ (SO_2_) stretching vibrations observed at 1089 cm^−1^ and 1332 cm^−1^ respectively in the free SPR IR spectrum both shifted to higher wavenumbers in the spectrum of complex **1a** (1092 cm^−1^ for ʋ_sym_ (SO_2_) and, 1335 cm^−1^ in for ʋ_asym_ (SO_2_)), as displayed in Fig. 2a,d and Table 2. These bands are not involved in the complexation of SPR to the metal but their shift to higher wavenumbers in the metal complex may be assigned to the participation of the SO_2_ group in the formation of hydrogen bond with the neighbouring atoms^29,36^. The ʋ(C=O) stretching vibration observed at 1639 cm^−1^ in the free SPR spectrum shifted to the lower wavenumber of 1634 cm^−1^ in the spectrum of complex **1a**. This confirms the involvement of the amide O in the complexation of SPR to the metal^29,36^. Both positive and negative shifts are also observed in the NH_2_ stretching vibrations of complex **1a** (3383 and 3164 cm^−1^ for SPR; 3325 and 3184 cm^−1^ for complex **1a**) while the NH stretching vibration at 3085 cm^−1^ in the free SPR spectrum shifted to a lower wavenumber of 3063 cm^−1^ in the spectrum of complex **1a**. The shifts in these bands may be assigned to either the keto-enol form or hydrogen bond formation^36,37^. The IR spectra of complexes **2a–5a** follow a similar trend to that observed for complex **1a**, as can be observed in Table 2. The IR spectra demonstrate that SPR binds to Ru (II) through the amide O and behaves as a neutral monodentate ligand.

**Table 2:** IR spectra (4000–650 cm^−1^) of the SPR drug and its ternary metal complexes.

Compound	ʋ (C=O)	ʋ _asym_ (SO_2_)	ʋ_sym_ (SO_2_)	ʋ = NH)	ʋ (NH_2_)
Sulpiride	1639	1332	1089	3085	3383, 3164
Complex 1a	1634	1335	1092	3063	3325, 3184
Complex 2a	1634	1335	1094	3063	3324, 3189
Complex 3a	1634	1335	1094	3063	3325, 3185
Complex 4a	1634	1335	1094	3071	3324, 3223
Complex 5a	1634	1335	1095	3190	3383, 3328

### NMR spectra with assignments of L1–5, complexes 1–5 and complexes 1a–5a

**Figure 3:**
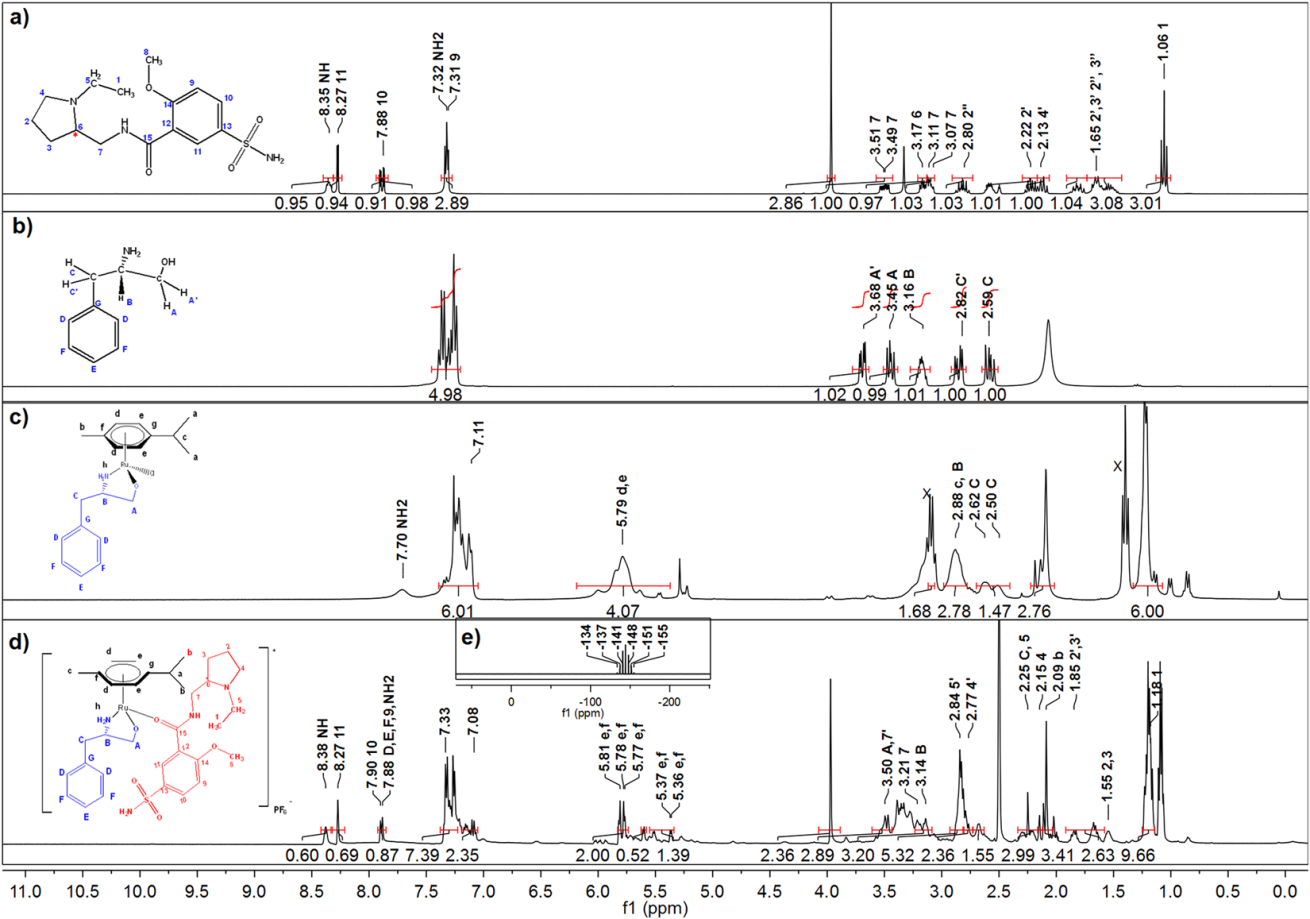
^1^H NMR spectra with assignments of (**a**) Sulpiride (SPR) in dmso-d6, (**b**) (R)-(+)-2-amino-3-phenyl-1-propanol (L1) in CDCl_3_-d, (**c**) [Ru(p-cymene)((R)-(+)-2-amino-3-phenyl-1-propanol)] (Complex **1**) in dmso-d6, (**d**) [Ru(p-cymene)((R)-(+)-2-amino-3-phenyl-1-propanol)(sulpiride)]PF_6_ (Complex **1a**) in dmso-d6 and the ^31^P NMR spectrum with assignments for (**e**) [Ru(p-cymene)((R)-(+)-2-amino-3-phenyl-1-propanol)(sulpiride)]PF_6_ (Complex **1a**) in dmso-d6.

**Figure 4:**
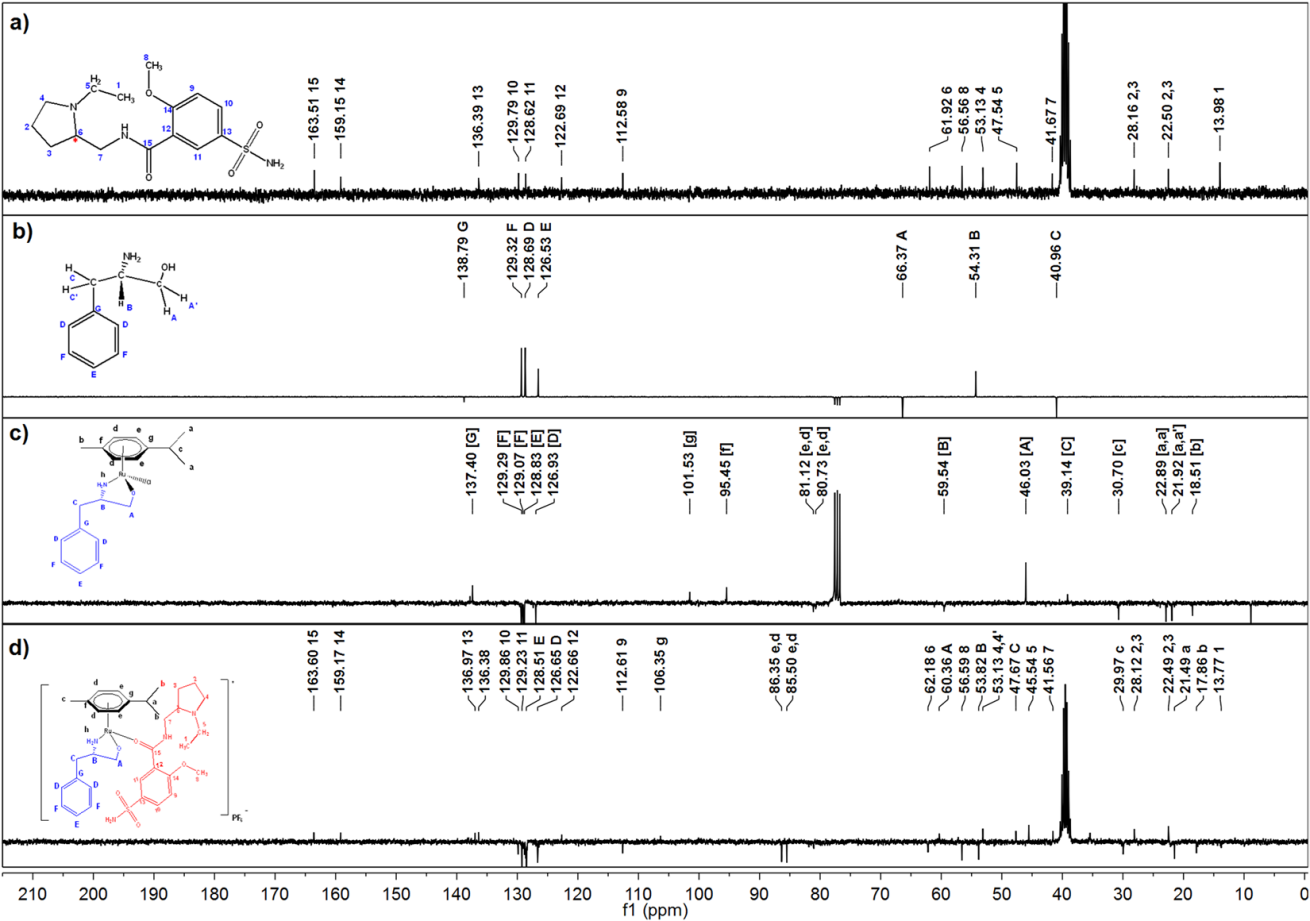
^13^ C NMR spectra with assignments of (**a**) Sulpiride (SPR) in dmso-d6, (**b**) (R)-(+)-2-amino-3-phenyl-1-propanol (L1) in CDCl_3_-d, (**c**) [Ru(p-cymene)((R)-(+)-2-amino-3-phenyl-1-propanol)] (Complex **1**) in dmso-d6 and d) [Ru(p-cymene)((R)-(+)-2-amino-3-phenyl-1-propanol)(sulpiride)]PF_6_ (Complex **1a**) in dmso-d6.

The ^1^H and ^13^C NMR spectra of L1–5 were compared to the spectra of complexes **1–5**. To be noted were the downfield shift in the NH_2_ band in the complexes, relative to the free ligands. For example, ^1^H NMR signal at δ 2.07 ppm was assigned to the protons attached to the nitrogen on L1 (Fig. 3b) and this peak appeared relative downfield (δ 7.70 ppm) on the spectrum of complex **1** (Fig. 3c). Additionally, two signals were assigned to the protons in C^A^H_2_(OH) in L1 (δ 3.68 and 3.45 ppm; Fig. 3b), which were replaced by a single signal, relative upfield in complex **1** for C^A^H_2_O (δ 3.06 ppm; Fig. 3c). A similar upfield shift was also observed for the proton attached to C^B^ in complex **1** (δ 2.88 ppm; Fig. 3c), relative to L1 (δ 3.16 ppm; Fig. 3b). These ^1^H NMR signals shifts are indicative of metal coordination to the ligand, especially since both N and O participate in bond formation between the metal and the ligand. Similar shifts (either upfield or downfield) were observed in the ^1^H NMR spectra of complexes **2–5**, as compared to L2–5. The ^13^C NMR spectrum of complex **1** (Fig. 4c), likewise, displayed shielded C^A^ (δ 39.14 ppm) and deshielded C^B^ (δ 59.54 ppm), compared to the ^13^C NMR spectrum of L1 (Fig. 4b) where these peaks appeared at δ 40.96 and 54.31 ppm respectively. A similar trend was observed in the ^13^C NMR spectra of complexes **2–5**, when compared to the ^13^C NMR spectra of L2–5. The appearance of new peaks in the ^1^H and ^13^C NMR spectra of complexes **1–5**, corresponding to the *p*-cymene molecule, was further proof that metal coordination took place (Figs 3 and 4).

The comparative ^1^H NMR spectra of SPR and complex **1a** are shown in Fig. 3a,d respectively. The presence of SPR in complex **1a** was confirmed by the appearance of new bands in its ^1^H NMR spectrum, corresponding to SPR. Several chemical shifts were observed upon complexation of SPR to the metal centre. For example, the signal at δ 8.35 ppm was attributed to the nitrogen proton in the free SPR (Fig. 3a) and this band appeared relatively downfield at δ 8.38 ppm in complex **1a** (Fig. 3d), as well as complexes **2a–5a**. The comparative ^13^C NMR spectra of SPR and complex **1a** are shown in Fig. 4a,d respectively. Compared to the ^13^C NMR spectrum of complex **1** (Fig. 4c), there are new signals in the ^13^C NMR spectrum of complex **1a** (Fig. 4d) that correspond to the signals of free SPR (Fig. 4a). The NMR signal at C^15^=O was of particular importance since the amide O has been previously shown to participate in the coordination of SPR to metal^37^. This peak, appearing at δ 163.5 ppm in free SPR (Fig. 4a, Table 3), was deshielded to δ 163.6 ppm for complexes **1a**–**3a** and to δ 163.7 ppm for complex **4a** (Fig. 4d, Table 3), confirming the binding of SPR to Ru (II) through the amide O as a neutral monodentate ligand. The proposed structures of complex **1** and complex **1a** are shown along with the NMR spectra (Figs 3 and 4). The chemical shifts were generally small, implying the minimal delocalisation of spin density from the metal into molecular orbitals of ligands^38^.

**Table 3:** Selected ^1^H NMR (500 MHz, DMSO-d6) and ^13^C NMR (126 MHz, DMSO-d6) chemical shifts (ppm) of sulpiride and complexes **1a–5a**.

	O-CH_3_		CH_2_(NH)		C=O
	^1^H NMR	^13^C NMR	^1^H NMR	^13^C NMR	^13^C NMR
Sulpiride	3.97 (s)	56.56	3.21 (m)	41.67	163.51
Complex 1a	3.97 (s)	56.58	3.21 (s)	41.56	163.6
Complex 2a	3.98 (m)	56.57	3,58 (s)	41.23	163.6
Complex 3a	3.97 (s)	56.57	3.51 (s)	41.54	163.6
Complex 4a	3.99 (s)	56.54	3.47 (m)	41.35	163.7
Complex 5a	3.98 (m)	56.58	3.29 (m)	41.10	164.1

The presence of the PF_6_^−^ counterion for complex **1a** was confirmed by the characteristic septet in ^31^P NMR spectrum (Fig. 3e) centred at −144.1 ppm. This is due to all 6 equivalent fluorine coupling with phosphorous.

### Thermal analyses (TG, DTG and DSC) studies of L1–5, complexes 1–5 and complexes 1a–5a

The TGA/DTG and DSC analyses of free SPR, the precursor complexes **1–5** and the final metal complexes **1a–5a** were carried out with heating rates of 10 °C.min^−1^ under nitrogen atmosphere and the weight loss was measured from ambient temperature to 400 °C and 900 °C for DSC and TGA/DTG respectively.The thermal analyses of SPR and its ternary Ru(II) metal complexes are summarised in Table 4, TGA thermograms were shown in Supplementary Figs S45–46 and DSC curves were shown in Supplementary Fig. S47–S48 respectively.

**Table 4:** Thermal analyses (TG and DSC) of SPR and its series of ruthenium (II) metal complexes.

Compound	TG range (°C)	N*	% Found (calcd)		Assignment	Metallic residue found (calcd %)	DSC endothermic peaks(°C)
			Weight loss	Total weight loss			
SPR	249–443	1	57.01 (59.16)		Loss of C_7_H_8_NO_4_S		180.1 (−), 287.1 (−)
	443–900	1	42.93 (40.71)	99.94 (99.87)	Loss of C_8_H_15_N_2_		
Complex 1a	30–343	1	54.047 (53.16)		Loss of SPR	RuO 15.78 (13.99)	73.68 (−), 236.1 (−), 281.7 (−)
	343–900	2	30.18 (31.55)	84.22 (84.71)	Loss of C_8_H_12_NO + C_6_H_6_		
Complex 2a	30–357	1	55.6 (58.42)		Loss of SPR	Ru 18.85 (16.00)	108.8 (−), 220.7 (−), 313.1 (−)
	357–900	2	26.03 (24.26)	81.65 (82.68)	Loss of C_2_H_7_NO + C_6_H_6_		
Complex 3a	30–341	1	56.7 (57.08)		Loss of SPR	RuO 17.78 (15.63)	137.3 (−), 223.2 (−), 286.1 (−)
	341–900	2	25.52 (25.85)	82.22 (82.93)	Loss of C_3_H_9_NO + C_6_H_6_		
Complex 4a	30–356	1			Loss of SPR	Ru 17.78 (15.63)	144.7 (−)
	356–900	2	57.54 (57.08)		Loss of C_3_H_9_NO + C_6_H_6_		306.2 (−), 316.9 (−)
			25.32 (25.85)	82.86 (82.93)			
Complex 5a	30–348	1	54.33 (54.88)		Loss of SPR	RuO 15.40 (15.03)	136.2 (−), 253.1 (−), 272.6 (−)
	348–900	2	30.24 (28.71)	84.57 (83.59)	Loss of C_5_H_11_NO + C_6_H_6_		

The TG curve of SPR shows a total weight loss of 99.94% (99.87%) which is observed in two successive decomposition steps. The first weight loss of 57.01% (59.16%) in the range of 249 to 443 °C may be assigned to the decomposition of the molecule C_7_H_8_NO_4_S. The second weight loss of 42.93 (40.71%), within the temperature range of 443 to 900 °C, is attributable to the decomposition of C_8_H_15_N_2_. The decomposition weight losses were found in agreement with the starting formula weight.

The TG curve of complex **1** displays two decomposition steps, corresponding to the loss of L1 in the TG range 30–250 °C (approximately 60% of complex **1**), followed by the loss of the *p*-cymene molecule in the TG range 250–900 °C (approximately 30% of complex **1**). The decomposition of complex **1** ended with Ru oxide (RuO) as a metallic residue.

The TG curves of the Ru (II) metal complexes of SPR are similar and show three decomposition steps in the temperature range 30 to 900 °C (Table 4). All complexes started decomposition with the loss of SPR and end with RuO as a metallic residue (Table 4). The first decomposition step happened in the range of 30 to 343 °C, during which 54.047% (53.16%) of complex **1a** was lost. In this step, SPR was lost from complex **1a** (Table 4). The second two decomposition steps were within the temperature range 343 to 900 °C, 30.18% (31.55%) of complex **1a** was lost. These steps correspond to the loss of L1, as well as the benzene ring from the Ru(*p*-cymene) molecule (Table 4). As depicted in Table 4, complexes **2a–5a** had a similar TG decomposition profile to complex **1a** in the temperature range 30 to 900 °C.

The DSC curve of SPR (Table 4) shows a sharp endothermic peak at 180.1 °C, which is its melting temperature^29^. A small and wide endothermic peak is observed at 287.1 °C (Table 4), which can be associated with some decompositions, reductions or phase transitions^39^.

The DSC curve of complex **1** shows two wide endothermic peaks at 147.2 °C and 285 °C, corresponding to the dehydration and the melting of the complex, respectively.

The DSC curve of complex **1a** shows three endothermic peaks (Table 4). The first one, observed at 73.68 °C, is small and wide and shows the dehydration of the molecule. The second wide endothermic peak at 236.1 °C corresponds to the melting temperature of complex **1a** (Table 4). Complex **1a** underwent further decomposition resulting in the final DSC peak at 281.7 °C (Table 4).

### Ultraviolet-Visible (UV-Vis) spectra of SPR, complexes 1–5 and complexes 1a–5a

**Figure 5:**
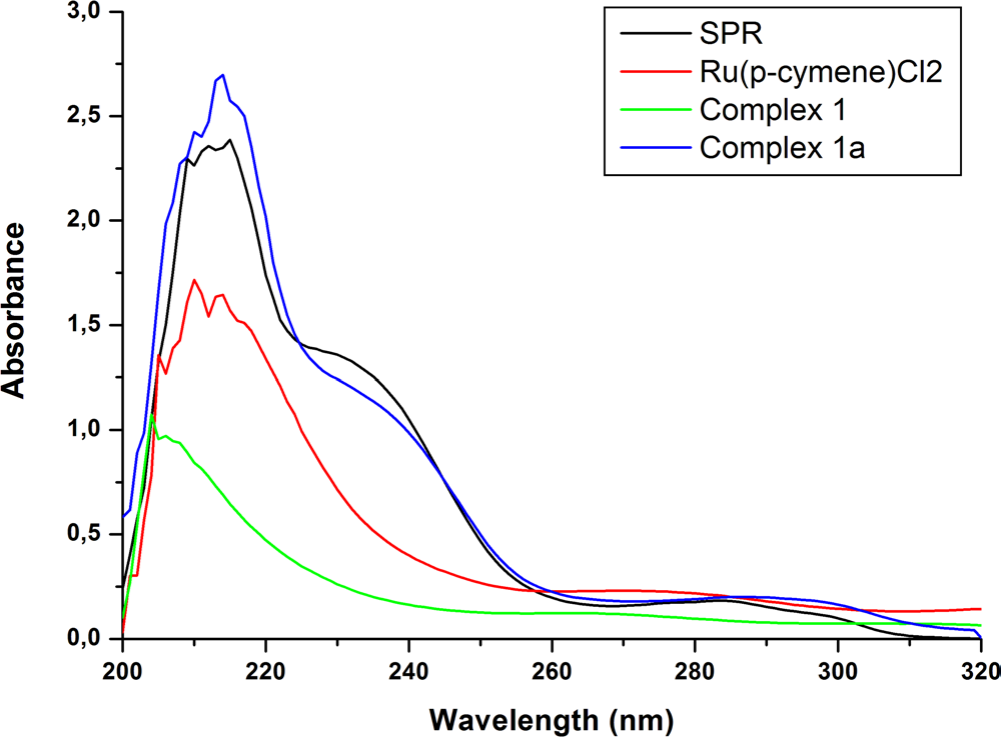
UV-Vis spectra of sulpiride (SPR), dichloro(p-cymene)ruthenium(II) dimer (Ru(p-cymene)Cl_2_), [Ru(p-cymene)((R)-(+)-2-amino-3-phenyl-1-propanol)] (Complex **1**) and [Ru(p-cymene)((R)-(+)-2-amino-3-phenyl-1-propanol)(sulpiride)]PF_6_ (Complex **1a**).

The UV-Vis spectra of SPR, complexes **1–5** and complexes **1a–5a** were recorded in the region 200–600 nm. Figure 5 shows the UV-Vis spectra of SPR, Ru(*p-*cymene)Cl_2_, complex **1** and complex **1a**. The spectrum of the SPR (20 mg/L in methanol) exhibited absorption maxima at 213 and 288 nm; this is in accordance with UV-Vis studies of levosulpiride previously conducted by Siddiqi *et al*., as well as Manjunath *et al*.^40,41^. For complex **1a** these bands shifted to 212 nm and 286 nm corresponding to a ligand-to-metal charge-transfer (LMCT) complex. There was also a presence of additional bands in the range of 300–350, which was most likely due to the exchange of the chlorido ligands from ruthenium arene complexes by water molecules, as previously reported by Rilak and co-workers^42^. This would involve spectral changes in the range of 300–350 nm. Based on these spectral differences it was possible to distinguish between the parent drug and the ruthenium complex **1a**. The spectra of complexes **1**–**5** and that of SPR all show bands in the range 210 to 220 nm. The quantification of SPR was therefore carried out using the absorbance value 288 nm, which was specific to the SPR UV-vis spectrum.

### Fluorescence study

**Figure 6:**
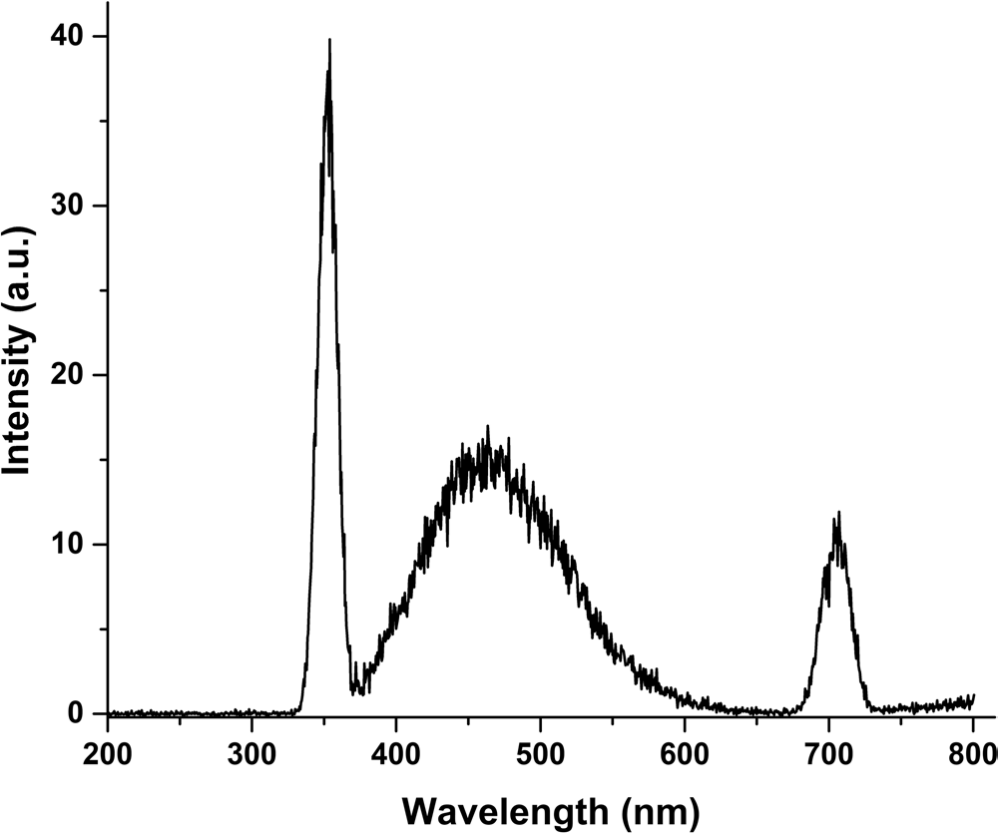
Fluorescence spectrum of complex **1a** in methanol.

The emission characteristics of complex 1a were examined in a methanol solution at a concentration of 3 × 10^−6^ mol/L at room temperature. The fluorescence spectrum was carried out at excitation wavelengths (λex) of 350 and 450 nm (Fig. 6) and the resulting emission (10 a.u.) was observed at 708 nm. Complex 1a showed relatively low emission, as compared to reported ruthenium complexes. In fact, a recent study reported a novel ruthenium-based anticancer scaffold with remarkable fluorescence intensity (400 a.u.), which was measured at a concentration of 4 × 10^−7^ mol/L in methanol^43^. Complex 1a displayed no significant luminescent behaviour. Complex 1a displayed weak MLCT due to the interaction of π* (benzene ring, electron rich group) of sulpiride and *d* electrons of ruthenium. Fluorescence studies revealed emissions originating from the lowest energy MLCT state, attributed to the excitation involving *d*_π- π_ligand*. Such emission properties of ruthenium (II) metal complexes were previously reported^44^.

### Solubility studies of free SPR and SPR in complexes 1a, 3a and 5a

The results of the solubility studies of free SPR and SPR in metal complexes are shown in Table 5. SPR in metal complexes showed improved solubility in all solvents tested compared to free SPR. The solubility of SPR in water was more than twice higher (695 mg/mL vs 1659, 1518 and 1549 mg/L for complexes **1a**, **3a** and **5a** respectively) following coordination of the drug to the metal. A similar but slightly lower trend is observed in PBS pH 6.8 and in PBS pH 7.4, while solubility improvement of SPR in methanol is also more than doubled with metal complexation.

**Table 5:** Solubility values of free SPR and SPR in Ru(II) metal complexes in different solvents (mg/L).

	Water (%RSD)	PBS pH 6.8 (%RSD)	PBS pH 7.4 (%RSD)	Methanol (%RSD)*
SPR	697 mg/L (1.18)	892 mg/L (2.99)	853 mg/L (1.83)	910 mg/L (2.80)
SPR in complex 1a	1668 mg/L (1.38)	1339 mg/L (1.64)	1287 mg/L (1.38)	1954 mg/L (3.35)
SPR in complex 3a	1529 mg/L (1.55)	1575 mg/L (2.79)	1494 mg/L (2.01)	2156 mg/L (2.29)
SPR in complex 5a	1551 mg/L (1.56)	1757 mg/L (1.18)	1621 mg/L (1.04)	2208 mg/L (2.28)

^*^Number of replicates for each solvent: 20.

Complexes **1a** ([Ru(p-cymene)(C_9_H_13_NO)(SPR)]PF_6_), **3a** ([Ru(p-cymene)(C_3_H_9_NO)(SPR)]PF_6_) and **5a** ([Ru(p-cymene)(C_5_H_11_NO)(SPR)]PF_6_) each contain two coligands (L1/3/5 and SPR) attached to the metal centre. The aqueous (water, PBS buffer) solubility improvement of complexed SPR compared to free SPR may be achieved from the presence of the water soluble amino alcohols L1, L3 and L5 as coligands of SPR in complexes **1a**, **3a** and **5a** respectively. These ancillary ligands influence the environment surrounding SPR, thereby positively affecting its water solubility. Such phenomenon has been previously observed in Ru(II) metal complexes^45^. The variation in complexed SPR solubility can be attributed to the differences in structure and solubility between L1, L3 and L5, which have an effect on their interactions with neighbouring molecules. Previous studies in this area have in fact shown the importance of the choice of coligand to achieve desired water solubility improvement of a drug through metal complexation^42^. Water and methanol are both polar molecules but methanol, with a polarity index value of 5.1, is somewhat less polar and therefore more lipophilic than water (polarity index 10.2)^46–48^. This difference in polarity explains the higher solubility of SPR in methanol, as compared to water. The dichloro(p-cymene)ruthenium(II) dimer present in complexes **1a**, **3a** and **5a** is known to be lipophilic and has been previously shown to improve the lipophilicity of the compounds complexed to its centre^42,49,50^. A similar phenomenon is observed in this study, with the highest solubility values of SPR obtained by dissolution of the metal complexes in the more lipophilic compound, methanol.

Ruthenium metal carrier therefore demonstrated the ability to improve the pharmaceutical profiles of drugs by improving both their aqueous and lipid solubility, which is advantageous for pharmaceutical formulation^51^.

### Dissolution studies of free SPR and SPR in complexes 1a, 3a and 5a

**Figure 7:**
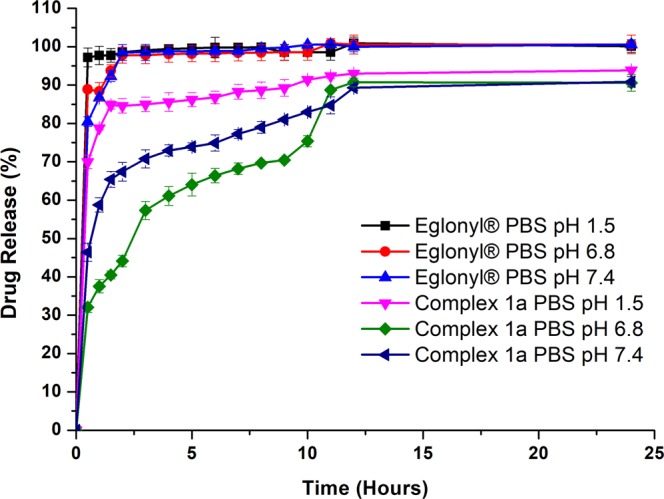
Dissolution profiles of sulpiride (SPR) and [Ru(p-cymene)((R)-(+)-2-amino-3-phenyl-1-propanol) (sulpiride)]PF_6_ (Complex **1a**) in PBS buffer at different pH values.

The dissolution profiles of Eglonyl®, SPR in complex **3a** and SPR in complex **5a** are similar to each other. In PBS pH 1.5, 6.8 and 7.4, there is a burst release of the drug within 30 minutes of the dissolution test. Approximately 90, 80 and 70% of the drug is released at pH 1.5, 6.8 and 7.4 respectively for Eglonyl® and complexes **3a** and **5a**. The total amount of the drug is fully released by 2 hours for all three compounds. Complex **1a** has a slower dissolution rate compared to Eglonyl® and the other two complexes. Figure 7 shows the dissolution profiles of Eglonyl® and SPR in complex **1a**. A burst release of SPR is observed 30 minutes after the start of the dissolution test of complex **1a** but it is lower than that observed for Eglonyl® and SPR in complexes **3a** and **5a** in PBS; 32% and 46% SPR are released from complex **1a** in PBS pH 6.8 and 7.4 respectively within 30 minutes of the dissolution test. At all pH values, total release of the drug from complex **1a** is observed by 24 hours.

Solubility and dissolution rate are directly proportional, increased solubility should therefore result in improved dissolution rate^52,53^. However, this is not observed. Although the solubility of complexed SPR in the dissolution media is improved, the dissolution rate of SPR from the metal complexes is slower than that of free SPR. The slowest dissolution rates were observed in SPR from complex **1a**.

The formation constant of [Ru(*p*-cymene)Cl(SPR)] was determined previously in this paper and the value of 5.45 was obtained, indicating the likelihood of the Ru(II)-SPR complex to dissociate in acidic environment^35,41^. The breakage of the metal-ligand bond is therefore easier in acidic media, explaining the fast dissolution of complexed SPR in PBS pH values 1.5 and pH 6.8. In the physiological pH of 7.4, on the other hand, the [Ru(*p*-cymene)Cl(SPR)] complex is expected to be more stable, thereby limiting the release of SPR. This behaviour was, however, only observed in SPR in complex **1a**. This can be attributed to the presence of coligands with different chemical structures and properties in the complexes, which may have an influence on the stability of the [Ru*(p* -cymene)Cl(SPR)] complex^45^. This would imply the ability of L1 as a coligand to SPR in complex **1a** to maintain or improve the stability of [Ru(*p*-cymene)Cl(SPR)], thus the slower dissolution of SPR from this complex, in comparison to free SPR and the other two complexes.

It has been shown that transition metal complexes are good candidates for controlled drug release because they possess bonds that are highly responsive to their environment^5^. In the particular case of ruthenium complexes, their various oxidation states, different mechanisms of action and kinetics give them several advantages, including low toxicity^54,55^. Few metal-based drugs reach their biological targets without any chemical modification, thus the importance of ligand exchange in biological activity. The mechanism of ligand exchange varies depending on both the metal and the coordinated ligand(s). The ligand exchange processes of ruthenium compounds are known to take place at slow rates in various cell lines, within the range of one to two hours, which are close to those of cellular processes^56,57^. This indicates that ruthenium complexes, when administered parenterally are not dissociated prior to any of their biological targets being reached. As a result, under physiological conditions (pH 7.4), metal interaction with nucleic acids, proteins and water could occur in the cells and such interactions are crucial for inducing the therapeutic effect of a drug^56,57^. The above-described properties of ruthenium complexes could be a further explanation for the selective release of SPR from the metal complex, leading to slower dissolution of SPR from complex **1a**. This is an advantage for SPR, as it reduces its initial burst release, which is one of the causes for its short half-life and its frequent dosing schedule^31,32^. However, if oral delivery of SPR is to be maintained, drug formulation strategies will need to be applied to protect the metal complex containing SPR from dissociation in the acidic environment. This shows the potential of ruthenium metal complexation to the appropriate ligands to achieve sustained release of a drug.

### Permeation studies of free SPR and SPR in complexes 1a, 3a and 5a

The values of the ionic conductivity of the porcine intestinal tissue at t_0_ and t_8_ were similar (18 and 17 mV respectively) and their FTIR spectra at these time points showed similar bands, both implying tissue structural integrity at t_8_. At t_12_ and t_24_, on the other hand, the ionic conductivities dropped to −8 and −22 mV but the FTIR spectra remained unchanged, implying that some of the integrity of the porcine intestinal tissue was compromised at these time points.

The permeation profiles of free SPR and SPR from the Ru (II) metal complexes through the pig’s small intestine were therefore determined up to 8 hours and are shown in Table 6.

**Table 6:** Cumulative permeability and cumulative relative permeability of free SPR, SPR released from metal complexes **1a**, **3a** and **5a**.

Compound	Cumulative Permeability (µg/cm^2^)	Cumulative Relative Permeability (%)
Sulpiride	88.42	14.17
Complex 1a	199.12	27.20
Complex 3a	185.64	27.09
Complex 5a	165.95	24.24

Higher amounts of SPR were permeated through the membrane from the metal complexes than the free drug. All metal complexes improved the permeation of SPR across the pig’s intestinal membrane by more than 10%. Complexation to Ru (II) therefore resulted in increased permeation of the drug across the pig’s intestine. This suggests that the lipophilicity of the drug is improved, thereby enhancing its diffusion through the membrane. Lipophilicity improvement through coordination to a ruthenium (II) arene molecule has been previously demonstrated and attributed to the presence of methyl groups in the ruthenium arene moiety, as is the case for dichloro(*p*-cymene)ruthenium(II) dimer^50^. The lipid soluble chemical groups on the metal positively affect the lipophilicity of SPR upon complexation. Coordination to the ruthenium metal could be used to enhance the lipid solubility and thus the intestinal membrane permeation of drugs.

### Effects of free SPR and SPR in complexes 1a, 3a and 5a on Caco-2 cell viability

**Figure 8:**
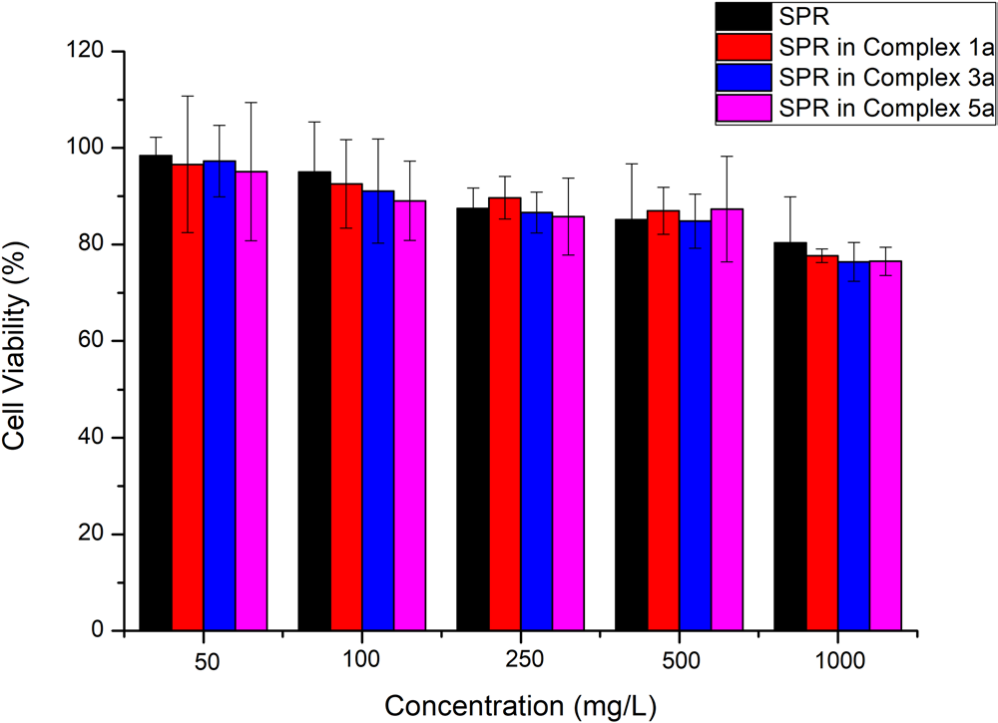
Percentage Caco-2 cell viability following treatment with SPR, complexes **1a**, **3a** and **5a**.

Figure 8 depicts the percentage cell viability after cell treatment with different concentrations of free SPR, SPR in complex 1a, SPR in complex 3a and SPR in complex 5a for 24 hours. No significant differences were observed in percentage cell viability of the complexed SPR, compared to the free SPR. Previously conducted intestinal absorption studies of SPR using the Caco-2 cell line to make *in vitro* model of the human intestine have also demonstrated minimal effect of SPR on Caco-2 cells^58,59^. Ruthenium (II) metal complexes, on the other hand, have been associated with anticancer activity against a variety of human cell lines, including Caco-2. Recent studies have in fact reported moderate to high (higher than cisplatin) *in vitro* toxicity of ruthenium arene complexes against Caco-2 cell line^60,61^. In these ruthenium complexes exhibiting anticancer properties, the metal in the molecule is active and therefore the tested concentrations are metal-dependent. This is not the case in the current study, where SPR is the active ligand and ruthenium is used as a drug carrier, thus the tested concentrations are SPR-dependent and contain less metal than metal-based concentrations. This lower ruthenium (II) concentration in ternary metal complexes of sulpiride could explain the lack of toxicity of the metal on the Caco-2 cells. It was noticed that the percentage cell viability slightly decreased with increased concentration of the tested compounds, which was expected, especially in the presence of ruthenium^62^. Free and complexed SPR thus demonstrated no noticeable toxic effects on the intestinal epithelium tissue.

## Conclusions

In this study, it is the first time that five ruthenium(II)-liganded sulpiride and amino alcohol complexes have been successfully synthesised in a 1:1:1 ratio (metal:drug:amino alcohol) and characterised. Subsequent *in vitro* studies showed improved aqueous solubility of sulpiride when complexed to the metal, slower dissolution rate of the drug from the metal complexes, enhanced permeation of the complexed drug through the pig’s intestine and low cytotoxicity of the metal complexes. These results demonstrate the potential of ruthenium-based metal carrier as a non-toxic drug carrier for aqueous solubility, sustained release and permeation enhancement. Formulation studies should be undertaken to improve the drug’s sustained delivery profile using ruthenium metal-based carrier and to avoid the metal-drug bond breakage in acidic environment, if oral drug delivery is to be maintained.

## Materials and Methods

### Materials

All chemicals used were of the analytical reagent grade and of the highest available purity. Sulpiride (SPR), (R)-(+)-2-amino-3-phenyl-1-propanol (C_9_H_13_NO, L1), ethanolamine (C_2_H_7_NO, L2), (S)-(+)-2-amino-1-propanol (C_3_H_9_NO, L3), 3-amino-1-propanol (C_3_H_9_NO, L4), (S)-(+)-2-pyrrolidinemethanol (C_5_H_11_NO, L5), triethylamine (TEA), ammonium hexafluorophosphate (NH_4_PF_6_) and dichloro(*p*-cymene)ruthenium(II) dimer were all purchased from Sigma-Aldrich and used as received. Sodium chloride, potassium chloride, disodium hydrogen phosphate and monopotassium phosphate were all purchased from Merck and used as received to prepare phosphate-buffered saline (PBS) following methods from the US Pharmacopeia. Eglonyl® 50 mg capsules were purchased from a local pharmacy. Organic solvents were purchased from Sigma-Aldrich and included dichloromethane (DCM), methanol (MeOH) and pentane. Dichloromethane and methanol were dried using a suitable drying agent under nitrogen and stored over 3 Å molecular sieves prior to use. Millipore water was used where needed.

### Instruments

Infrared spectra were recorded in the wavenumber region 4000–650 cm^−1^ on a Spectrum 100 FTIR spectrometer (Perkin-Elmer Inc. MA, USA) equipped with the attenuated total reflectance (ATR) sampling device. ^1^H, ^13^C and ^31^P NMR spectra were recorded on a 300 MHz Bruker AVANCE II and 500 MHz Bruker AVANCE II spectrometer (Bruker Avance Biospin Germany) at the Department of Chemistry of the University of the Witwatersrand (Johannesburg, South Africa). All signals were confirmed by the ^1^H-^1^H COSY and ^1^H-^13^C HSQC experiments. A temperature-modulated differential scanning calorimeter (Mettler Toledo DSC1 STARe System, Switzerland) was used to investigate the thermal behaviour of the metal complexes. The thermogravimetric (TG and DTG) analyses were performed under a nitrogen atmosphere with a heating rate of 10 °C.min^−1^ using the Thermogravimetric Analyzer TGA 4000 (Perkin-Elmer Inc. MA, USA). The UV-Vis measurements were recorded on a Lambda 25 UV/VIS Spectrophotometer (Perkin-Elmer Inc. MA, USA). The fluorescence spectrum was recorded on a Perkin Elmer LS-40 fluorescence spectrophotometer (Perkin-Elmer Inc. MA, USA).

### Formation/Dissociation constant of the complex [Ru(p-cymene)Cl(SPR)]

#### Preparation of 1 × 10^−1^ M [Ru(*p*-cymene)Cl_2_] and 1 × 10^−1^ M SPR

[Ru(*p*-cymene)Cl_2_] (0.6124 g, 1 mmol, M. Wt. = 612.4 g.mol^−1^) was dissolved in dry methanol and made up to the mark in a 100 mL volumetric flask.

SPR (0.34 g, 1 mmol, M. Wt. = 341.43 g.mol^−1^) was dissolved in dry methanol and made up to the mark in a 100 mL volumetric flask.

#### Procedure for continuous variation method (Job’s method)

The stoichiometric ratio of SPR to Ru(II) in the complex was determined by Job’s method of equimolar solutions^63,64^. Ru(*p*-cymene)Cl_2_ 1 × 10^−1^ M stock solution (0, 1, 2, …, 6 mL) was pipetted out and transferred into seven 50 mL volumetric flasks and an aliquot (6, 5, …, 0 mL) of 1 × 10^−1^ M SPR was added, respectively in such a way that the mole fraction of solution remained constant. The colour of the solution changed from brown to orange. Wavelength of maximum absorbance was noted against a blank, which appeared at 296 nm. All the measurements were made at 296 nm. The following equations were used to calculate the stability constant (K) and the dissociation constant (K_d_):

1 $${\rm{K}}=\frac{[{\boldsymbol{ML}}]}{[{\boldsymbol{M}}]x\,[{\boldsymbol{L}}]\,}={}^{\underline{[\frac{{\boldsymbol{A}}2}{{\boldsymbol{A}}1}]}}[1-\frac{{\boldsymbol{A}}2}{{\boldsymbol{A}}1}]\ast [{\boldsymbol{C}}({\boldsymbol{SPR}})-{\boldsymbol{C}}({\boldsymbol{Ru}})\ast \frac{{\boldsymbol{A}}2}{{\boldsymbol{A}}1}]$$

Where M = amount of metal ion, L = amount of ligand, A1 = absorbance at break point, A2 = actual absorbance, C(SPR) = concentration of sulpiride and C(Ru) = concentration of ruthenium.

2 $${{\rm{K}}}_{{\rm{d}}}=\frac{{\bf{1}}}{{\boldsymbol{K}}}$$

Where K = formation constant and K_d_ = dissociation constant.

### Synthesis of metal complexes

**Figure 9:**
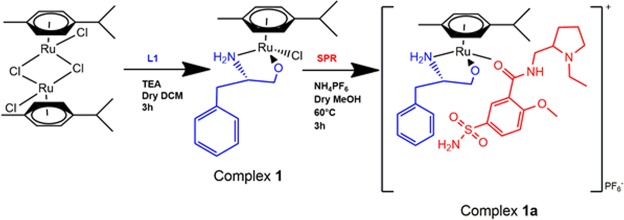
Synthesis procedure of ternary metal complexes of sulpiride. L1 is used as an example of ancillary ligand.

All metal complexations were carried out under inert atmosphere of nitrogen. Dichloromethane and methanol were dried using a suitable drying agent under nitrogen and stored over 3 Å molecular sieves prior to use. The synthesis procedure is summarised in Fig. 9.

#### Representative synthesis of the precursor complexes 1–5

The synthesis of the precursor metal complexes was adapted from literature^65,66^. Respective dry Schlenk tubes were charged with 20 mL dry DCM, amino alcohol (1.633 mmol) and TEA (1.633 mmol; 228 µL) and left to stir for 30 minutes at room temperature. Complexes **1–5** required L1: 247 mg, L2: 100 mg, L3: 123 mg, L4: 123 mg and L5: 165 mg respectively. Dichloro(*p*-cymene)ruthenium(II) (0.816 mmol; 500 mg) was then added. The resulting orange solutions were left to stir for 3h at room temperature and thereafter solvent was removed *in vacuo* to afford the complexes **1–5**.

[Ru(p-cymene)Cl(L1)](**1**)

Physical state: orange powder. Yield: 657 mg (95.36%). Melting point: 160 °C. Selected IR absorption bands (ATR, cm^−1^): 871.6 (*p*-substituted aromatic ring), 730, 1585 (N-H bending), 1087 (C-H in plane bending), 871.6 (C-H out of plane bending), 1092.41. ^1^H NMR (500 MHz, chloroform-*d*) δ 7.70 (s, 2H, NH_2_; L1), 7.22–7.11 (m, 5H, CH, CH, CH, CH, CH overlapping; L1), 5.79 (m, 4H, CH, CH, CH, CH; *p*-cymene), 3.06 (m, 2H, CH_2_O; L1), 2.88 (s, 1H, CH; *p*-cymene), 2.88 (s, 1H, CH(NH_2_); L1), 2.62 (s, H, CH; L1), 2.50 (s, H, CH; L1), 2.09 (m, 3H, CH_3_; *p*-cymene), (1.23, m, 6H, CH_3_, CH_3_; *p*-cymene). ^13^C NMR (126 MHz, Chloroform-*d*) δ 137.4 (C, OH; L1), 129.3 (CH, CH; L1), 129.1 (CH, CH; L1), 128.9 (CH; L1), 126.9 (CH, CH; L1), 101.5 (C; *p*-cymene), 95.45 (C; *p*-cymene), 81.12 (CH, CH; *p*-cymene), 80.73 (CH, CH; *p*-cymene), 59.54 (CH(NH_2_)); L1), 46.03 (CH_2_; L1), 39.14 (CH_2_O; L1), 30.70 (CH; *p*-cymene); 22.89 (CH_3_; *p*-cymene), 21.92 (CH_3_; *p*-cymene), 18.51 (CH_3_; *p*-cymene). UV-VIS. λ_max_ (nm)_:_ 219, 319.

[Ru(p-cymene)Cl(L2)] (**2**)

Physical state: yellow residue. Yield: 445 mg (82.13%). Melting point: 148 °C. Selected IR absorption bands (ATR, cm^−1^): 875.4 (p-substituted), 730.8 (C-H bending), 1595 (C = C). ^1^H NMR (500 MHz, Chloroform-*d*) δ 5.57 (d, *J* = 5.8 Hz, 2H, CH, CH; *p*-cymene), 5.42 (d, *J* = 5.7 Hz, 2H, CH, CH, *p*-cymene), 4.76 (s, 2H; NH_2_), 3.75 (bs, 2H, CH_2_O; L2), 3.14 (bs, 2H, CH_2_(NH_2_), L2), 2.95 (dt, *J* = 13.5, 6.8 Hz, 1H, CH; *p*-cymene), 2.35 (s, 3H, CH_3_; *p*-cymene), 1.29 (d, *J* = 6.9 Hz, 6H, CH_3_, CH_3_; *p*-cymene). ^13^C NMR (126 MHz, Chloroform-*d*) δ 105.32 (C; *p*-cymene), 95.22 (C; *p*-cymene), 82.76 (CH, *p*-cymene), 80.93 (CH, *p*-cymene), 62.63 (CH_2_O; L2), 52.28 (CH_2_(NH_2_); L2), 31.03 (CH; *p*-cymene), 22.49 (CH_3_; *p*-cymene), 18.48 (CH_3_; *p*-cymene). UV-VIS. λ_max_ (nm)_:_ 217, 322.

[Ru(p-cymene)Cl(L3)] (**3**)

Physical state: brick red powder. Yield: 535 mg (94.74%). Melting point: 126 °C. Selected IR absorption bands (ATR, cm^−1^): 870.5 (p-substituted), 802.8 (C-H bending), 1087 (C-H in plane bending), 3188 (C-H stretching). ^1^H NMR (500 MHz, Chloroform-*d*) δ 7.93 (s, 2H, NH_2_; L3), 5.95 (d, *J* = 5.3 Hz, 1H, CH; *p*-cymene), 5.85 (s, 1H, CH; *p*-cymene), 5.82 (d, *J* = 5.4 Hz, 1H; *p*-cymene), 5.60 (m, 1H, CH; *p*-cymene), 3.24 (m, 2H, CH_2_; L3), 2.90 (dt, *J* = 16.0, 8.4 Hz, 1H, CH; L3), 2.76 (s, 1H, CH; *p*-cymene), 2.12 (m, 3H, CH_3_; *p*-cymene), 1.25 (dd, *J* = 6.8, 1.8 Hz, 6H, CH_3_, CH_3_; *p*-cymene), 1.20 (d, *J* = 6.0 Hz, 3H, CH_3_; L3). ^13^C NMR (126 MHz, Chloroform-*d*) δ 101.45 (C; *p*-cymene), 95.37 (C; *p*-cymene), 81.02 (CH; *p*-cymene), 80.74 (CH; *p*-cymene), 80.06 (CH; *p*-cymene), 78.58 (CH; *p*-cymene), 67.13 (CH_2_O; L3), 39.16 (CH(NH_2_); L3), 30.70 (CH; *p*-cymene), 22.91 (CH_3_; L3), 21.90 (CH_3_, *p*-cymene), 18.47 (CH_3_, *p*-cymene).UV-Vis λ_max_ (nm)_:_ 218, 319.

[Ru(p-cymene)Cl(L4)] (**4**)

Physical state: brick red residue. Yield: 462 mg (81.91%). Melting point: 126 °C. Selected IR absorption bands (ATR, cm^−1^): 861.3 (p-substituted), 697.7, 1579 (N-H bending), 731.2 (C-H out of plane bending), 1465, 1579 (C-C stretching). ^1^H NMR (500 MHz, Chloroform-*d*) δ 5.45 (d, *J* = 5.9 Hz, 2H, CH, CH; *p* -cymene), 5.37 (d, *J* = 5.9 Hz, 2H, CH, CH; *p*-cymene), 3.75 (t, *J* = 5.1 Hz, 2H, CH_2_(NH); L4), 3.23 (s, 2H, CH_2_O; L4), 2.97 (hept, *J* = 6.9 Hz, 1H, CH, L4), 2.24 (s, 3H, CH_3_; *p*-cymene), 1.76 (m, 2H, CH_2_; L4), 1.30 (d, *J* = 6.9 Hz, 6H, CH_3_; *p*-cymene). ^13^C NMR (126 MHz, Chloroform-*d*) δ 102.7 (C; *p*-cymene), 95.80 (C; *p*-cymene), 81.22 (CH; *p*-cymene), 80.41 (CH; *p*-cymene), 60.71 (CH_2_O; L4), 47.61 (CH_2_(NH); L4), 34.65 (CH_2_; L4), 31.02 (CH; *p*-cymene), 22.40 (CH_3_; *p*-cymene), 18.86 (CH_3_; *p*-cymene). UV-Vis λ_max_ (nm)_:_ 217, 320.

[Ru(p-cymene)Cl(L5)] (**5**)

Physical state: dark orange powder. Yield: 558 mg (91.89%). Melting point: 132 °C. Selected IR absorption bands (ATR, cm^−1^): 872.9 (p-substituted), 729.2, 1469 (N-H bending), 731.2 (C-H out of plane bending), 3081 (N-H stretching), 802.7 (C-H out of plan bending). ^1^H NMR (500 MHz, Chloroform-*d*) δ 8.45 (s, 1H, NH; L5), 6.01 (d, *J* = 32.0 Hz, 1H, CH; *p*-cymene), 5.83 (1H, CH, *p*-cymene), 5.78 (d, *J* = 46.1 Hz, 1H; *p*-cymene), 5.73 (1H, CH, *p*-cymene), 3.29 (s, 2H, CH_2_O; L5), 2.97 (s, 1H, CH; *p*-cymene), 2.20 (s, 2H, CH_2_(NH); L5), 2.13 (m, 3H, CH_3_; *p*-cymene), 1.82 (s, 2H, CH_2_; L5), 1.30 (m, 2H, CH_2_; L5), 1.25 (m, 3H, CH_3_; *p*-cymene). ^13^C NMR (126 MHz, Chloroform-*d*) δ 101.66 (C; *p*-cymene), 94.20 (C; *p*-cymene), 81.42 (CH; *p*-cymene), 81.06 (CH; *p*-cymene), 80.66 (CH; *p*-cymene), 79.85 (CH; *p*-cymene), 68.61 (CH_2_O; L5), 62.13 (CH(NH); L5), 49.40 (CH_2_(NH); L5), 30.67 (CH; *p*-cymene); 24.87 (CH_2_; L5), 21.65 (CH3; *p*-cymene), 18.28 (CH_3_; *p*-cymene). UV-Vis λ_max_ (nm)_:_ 217, 318.

#### Representative synthesis of the final complexes **1a–5a**

The synthesis of the final metal complexes **1a–5a** was adapted from literature^67,68^. To respective solutions of complexes 1–5 (1.541 mmol, 1.341 mmol, 1.533 mmol, 1.336 mmol and 1.479 mmol respectively) in 20 mL methanol at 60 °C, NH_4_PF_6_ was added (1.541 mmol, 1.341 mmol, 1.533 mmol, 1.336 mmol and 1.479 mmol respectively). The resulting orange mixtures were left to stir for 30 minutes at 60 °C. SPR was then added (1.541 mmol, 1.341 mmol, 1.533 mmol, 1.336 mmol and 1.479 mmol respectively). The resulting orange solutions were left to stir for 3h at 60 °C under reflux and thereafter solvent was removed *in vacuo* to afford the complexes **1a–5a**. Residues were obtained which were solubilised in DCM and layered with pentane to afford the products. A cannula was used to filter the products, which were then dried *in vacuo*.

[Ru(*p*-cymene)(L1)(SPR)]PF_6_ (**1a**)

Physical state: mustard yellow powder. Yield: 1.08 g (92.15%). Melting point; 237 °C. Elemental anal. calcd. for C_34_H_50_F_6_N_4_O_5_PRuS: C, 46.84; H, 5.66; N, 6.43, S, 3.68. Found: C, 46.18; H, 5.60; N, 6.28, S, 3,64. Selected IR absorption bands (ATR, cm^−1^): 3325, 3184 (ʋ, NH_2_), 3064 (δ, = NH), 1634 (ʋ, C = O), 1335 (ʋ_asym_, SO_2_), 1092 (ʋ_sym_, SO_2_). ^1^H NMR (500 MHz, DMSO-*d6*) δ 8.38 (s, 1H, NH; SPR), 8.27 (d, *J* = 2.4 Hz, 1H, CH; SPR), 7.90 (dd, J = 8.7, 2.4 Hz, 1H, CH; SPR), 7.88 (m, 2H, NH_2_; SPR), 7.88 (m, 1H, CH; SPR), 7.88 (m, 5H, CH, CH, CH, CH, CH overlapping; L1), 5.81 (m, 1H, CH; *p*-cymene), 5.76 (s, 1H, CH; *p*-cymene), 5.37 (m, 2H, CH, CH; *p*-cymene), 3.97 (m, 3H, CH_3_O; SPR), 3.50 (m, 2H, CH_2_O; L1), 3.21 (s, 2H, CH_2_(NH); SPR), 3.14 (m, 1H, CH(NH_2_); L1), 2.84 (m, 2H, CH_2_; SPR), 2.77 (s, 1H, CH; SPR); 2.25 (m, 2H, CH_2_; L1), 2.15 (m, 1H, CH; SPR); 2.09 (m, 6H, CH3, CH3; *p*-cymene), 1.85 (m, 2H, CH_2_; SPR), 1.55 (m, 2H, CH_2_; SPR), 1.20 (d, J = 6.9 Hz, 1H, CH: *p*-cymene), 1.18 (m, 3H, CH_3_; SPR). ^13^C NMR (126 MHz, DMSO-*d6*) δ 163.6 (C = O; SPR), 159.2 (CO; SPR), 137 (CS; SPR), 129.9 (CH; SPR), 129.2 (CH; SPR), 128.5 (CH; L1), 126.7 (CH, L1), 122.7 (C; SPR), 112.6 (CH; SPR), 106.4 (C; *p*-cymene), 86.35 (CH; *p*-cymene), 85.50 (CH; *p*-cymene); 62.18 (CH; SPR), 60.36 (CH2O; L1), 56.58 (CH_3_O; SPR); 53.82 (CH(NH_2_); L1), 53.15 (CH_2_; SPR), 47.67 (CH_2_; L1), 45.54 (CH_2_; SPR), 29.97 (CH_3_; *p*-cymene), 28.12 (CH_2_; SPR), 22.49 (CH_2_; SPR), 21.49 (CH; *p*-cymene), 17.86 (CH_3_; *p*-cymene), 13.77 (CH_3_; SPR). ^31^P-NMR (202 MHz, DMSO-*d*_6_) δ -144.19 (d, *J* = 711.3 Hz). UV-Vis λ_max_ (nm)_:_ 286, 340,377.

[Ru(*p*-cymene)(L2)(SPR)]PF_6_ (**2a**)

Physical state: brown residue. Yield: 0.602 g (70.40%). Melting point: 222 °C. Elemental anal. calcd. for C_27_H_44_F_6_N_4_O_5_PRuS: C, 41.40; H, 5.62; N, 7.15, S, 4.08. Found: C, 41.21; H, 5.39; N, 7.11, S, 4.00. Selected IR absorption bands (ATR, cm^−1^): 3324, 3189 (ʋ, NH_2_), 3063 (δ, = NH), 1634 (ʋ, C = O), 1334 (ʋ_asym_, SO_2_), 1094 (ʋ_sym_, SO_2_). ^1^H NMR (500 MHz, DMSO-*d*_6_) δ 8.55 (s, 1H, NH; SPR), 8.25 (d, *J* = 2.2 Hz, 1H, CH; SPR), 7.90 (dd, *J* = 8.7, 2.4 Hz, 1H, CH; SPR), 7.34 (s, 1H, CH; SPR), 7.32 (s, 2H, NH_2_; SPR), 5.61 (m, 2H, CH, CH; *p*-cymene), 5.44 (m, 2H, CH, CH; *p*-cymene), 3.98 (m, 3H, CH_3_O; SPR), 3.58 (s, 2H, CH2(NH); SPR), 3.47 (m, 1H, CH; SPR), 3.47 (m, 1H, CH_2_O; L2), 2.92 (s, 2H, CH_2_(NH_2_); L2), 2.85 (s, 2H, CH_2_; SPR), 2.81 (d, *J* = 17.3 Hz, 1H, CH, *p*-cymene), 2.17 (s, 3H, CH_3_; *p*-cymene), 2.13 (m, 2H, CH_2_; SPR), 1.77 (m, 4H, CH_2_, CH_2_; SPR), 1.22 (d, *J* = 6.8 Hz, 6H, CH_3_, CH_3_; *p*-cymene), 1.17 (d, *J* = 2.7 Hz, 3H, CH_3_; SPR). ^13^C NMR (126 MHz, DMSO-*d*_6_) δ 163.6 (C = O; SPR), 159.2 (CO; SPR), 136.4 (CS; SPR), 129.8 (CH; SPR), 128.6 (CH; SPR), 122.7 (C; SPR), 112.6 (CH; SPR), 82.50 (CH; *p*-cymene), 80.26 (CH; *p*-cymene), 61.35 (CH; SPR), 61.30 (CH_2_O; L2), 56.57 (CH_3_O; SPR), 53.12 (CH_2_; SPR), 51.46 (CH_2_(NH_2_); L2); 47.63 (CH_2_; SPR), 41.23 (CH_2_(NH); SPR), 30.24 (CH; *p*-cymene), 28.11 (CH_2_; SPR), 22.47 (CH_2_; SPR), 21.94 (CH_3_; *p*-cymene), 17.43 (CH_3_; *p*-cymene), 13.77 (CH_3_; SPR). UV-Vis λ_max_ (nm)_:_ 280, 342, 378.

[Ru(*p*-cymene)(L3)(SPR)]PF_6_ (**3a**)

Physical state: mustard yellow powder. Yield: 1.04 g (98.7%). Melting point; 226 °C. Elemental anal. calcd. for C_28_H_46_F_6_N_4_O_5_PRuS: C, 42.18; H, 5.77; N, 7.03, S, 4.02. Found: C, 41.95; H, 5.67; N, 7.00, S, 3.97. Selected IR absorption bands (ATR, cm^−1^): 3325 3185 (ʋ, NH_2_), 3063 (δ, = NH), 1634 (ʋ, C=O), 1335 (ʋ_asym_, SO_2_), 1094 (ʋ_sym_, SO_2_). ^1^H NMR (500 MHz, DMSO-*d*_6_) δ 8.39 (s, 1H, NH; SPR), 8.27 (d, *J* = 2.4 Hz, 1H, CH; SPR), 7.89 (dd, *J* = 8.7, 2.5 Hz, 1H, CH; SPR), 7.33 (d, *J* = 8.8 Hz, 1H, CH, SPR), 7.33 (2H, NH_2_; SPR), 5.79 (d, *J* = 15.3 Hz, 4H, CH, CH, CH, CH; *p*-cymene), 3.97 (s, 3H, CH_3_O; SPR), 3.51 (s, 2H, CH_2_(NH); L3), 3.38 (s, 1H, CH; SPR), 3.23 (s, 2H, CH_2_O; L3), 2.88 (bs, 1H, CH(NH_2_); L3), 2.88 (bs, 1H, CH; *p*-cymene), 2.88 (bs, 2H, CH_2_; SPR), 2.69 (s, 1H, CH; SPR), 2.24 (s, 1H, CH; SPR), 2.09 (s, 3H, CH_3_; *p*-cymene), 1.69–1.55 (m, 4H, CH_2_, CH_2_; SPR), 1.19 (m, 6H, CH_3_, CH_3_; *p*-cymene), 1.11 (m,3H, CH_3_; SPR), 1.08 (m, 3H, CH_3_; L3). ^13^C NMR (126 MHz, DMSO-*d*_6_) δ 163.6 (C=O; SPR), 159.2 (CO; SPR), 136.4 (CS; SPR), 129.8 (CH; SPR), 128.6 (CH; SPR), 122.7 (C; SPR), 112.6 (CH; SPR), 106.4 (C; *p*-cymene), 100.1 (C; *p*-cymene), 86.33 (CH; *p*-cymene), 85.49 (CH; *p*-cymene), 62.53 (CH; SPR), 62.17 (CH_2_O; L3), 56.57 (CH_3_O; SPR), 53.12 (CH; SPR), 48.45 (CH(NH_2_); L3), 47.65 (CH; SPR), 41.54 (CH(NH); SPR), 29.95 (CH; *p*-cymene), 28.10 (CH_2_; SPR), 22.46 (CH_2_; SPR), 21.48 (CH_3_; *p*-cymene), 17.84 (CH_3_; *p*-cymene), 14.99 (CH_3_; L3), 13.74 (CH_3_; SPR). UV-Vis λ_max_ (nm): 289, 345,377.

[Ru(*p* -cymene)(L4)(SPR)]PF_6_ (**4a**)

Physical state: brown residue. Yield: 0.658 g (75.57%). Melting point: 260 °C. Elemental anal. calcd. for C_28_H_46_F_6_N_4_O_5_PRuS: C, 42.18; H, 5.77; N 7.03, S, 4.02. Found: C, 42.11; H, 5.74; N, 6.96, S, 4.01. Selected IR absorption bands (ATR, cm^−1^): 3324, 3223 (ʋ, NH_2_), 3071 (υ, = NH), 1634 (ʋ, C = O), 1335 (ʋ_asym_, SO_2_), 1093 (ʋ_sym_, SO_2_). ^1^H NMR (500 MHz, DMSO-*d*_6_) δ 8.66 (s, 1H, NH; SPR), 8.25 (m, 1H, CH; SPR), 7.91 (dd, *J* = 8.7, 2.2 Hz, 1H, CH; SPR), 7.34 (s, 2H, NH_2_;SPR), 7.32 (s, 1H, CH; SPR), 5.61 (d, *J* = 5.5 Hz, 1H, CH; *p*-cymene), 5.52 (q, *J* = 5.9, 5.0 Hz, 1H,CH; *p*-cymene), 5.45 (d, *J* = 6.0 Hz, 1H,CH; *p* -cymene), 5.41 (d, *J* = 5.7 Hz, 1H,CH; *p* -cymene), 3.99 (s, 3H, CH_3_O; SPR), 3.47 (m, 2H, CH_2_(NH); SPR), 3.45 (m, 2H, CH_2_O; L4), 3.39 (m, 1H, CH; SPR), 2.90 (m, 2H, CH_2_; SPR), 2.88 (m, 2H, CH_2_(NH); L4), 2.81 (dt, *J* = 13.9, 5.6 Hz, 1H, CH; *p*-cymene), 2.13 (m, 3H, CH_3_; *p*-cymene), 1.84 (m, 2H, CH_2_; SPR), 1.65 (m, 2H, CH_2_; SPR), 1.64 (dt, *J* = 13.1, 6.6 Hz, 2H, CH_2_; L4), 1.20 (d, *J* = 6.9 Hz, 6H, CH_3_, CH_3_; *p*-cymene), 1.18 (m, 3H, CH_3_; SPR). ^13^C NMR (126 MHz, DMSO-*d*_6_) δ 163.7 (C = O; SPR), 159.1 (CO; SPR), 136.4 (CS; SPR), 129.8 (CH; SPR), 128.6 (CH; SPR), 122.6 (C; SPR), 112.6 (CH; SPR), 102.7 (C; *p*-cymene), 95.27 (C; *p*-cymene), 86.29 (CH; *p*-cymene), 85.44 (CH; *p*-cymene), 62.51 (CH; SPR), 58.65 (CH_2_O; L4), 56.54 (CH_3_O; SPR), 53.04 (CH; SPR), 47.74 (CH; SPR), 46.95 (CH(NH); L4), 46.56 (CH; L4), 41.35 (CH_2_(NH); L4), 34.99 (CH; L4), 34.83 (CH; L4), 28.01 (CH_2_; SPR), 22.38 (CH_2_; SPR), 21.95 (CH_3_; *p*-cymene), 21.44 (CH_3_; *p*-cymene), 17.51 (CH_3_; *p*-cymene), 13.58 (CH_3_; SPR). UV-Vis λ_max_ (nm): 287, 345, 377.

[Ru(*p* -cymene)(L5)(SPR)]PF_6_ (**5a**)

Physical state: mustard yellow powder. Yield: 1.04 g (98.6%). Melting point; 252 °C. Elemental anal. calcd. for C_30_H_48_F_6_N_4_O_5_PRuS: C, 43.76; H, 5.84; N 6.81, S, 3.89. Found: C, 43.61; H, 5.74; N, 6.75, S, 3,80. Selected IR absorption bands (ATR, cm^−1^): 3383, 3328 (ʋ, NH_2_), 3190 (υ, = NH), 1634 (ʋ, C = O), 1335 (ʋ_asym_, SO_2_), 1095 (ʋ_sym_, SO_2_). ^1^H NMR (500 MHz, DMSO-*d*_6_) δ 8.44 (s, 1H, NH; L5), 8.26 (s, 1H, CH; SPR), 7.90 (d, *J* = 8.7 Hz, 1H, CH; SPR), 7.34 (s, 1H, CH; SPR), 7.32 (s, 2H, NH_2_; SPR), 5.79 (d, *J* = 15.8 Hz, 2H, CH, CH; *p*-cymene), 5.48 (dd, *J* = 118.6, 5.5 Hz, 2H, CH, CH; *p*-cymene), 3.98 (s, 3H, CH_3_; SPR), 3.53 (m, 1H, CH; SPR), 3.29 (s, 2H, CH_2_(NH); SPR), 3.12 (s, 2H, CH_2_O; L5), 2.98 (s, 2H, CH_2_(NH); L5), 2.94 (m, 2H, CH_2_; SPR), 2.82 (s, 1H, CH; *p*-cymene), 2.36 (s, 2H, CH_2_; SPR), 2.10 (d, *J* = 11.2 Hz, 3H, CH_3_; *p*-cymene), 1.88 (m, 2H, CH_2_; SPR), 1.72 (m, 2H, CH_2_; L5), 1.60 (m, 2H, CH_2_; SPR), 1.20 (s, 3H, CH_3_; *p*-cymene), 1.11 (s, 3H, CH_3_; SPR). ^13^C NMR (126 MHz, DMSO-*d*_6_) δ 164.10 (C=O; SPR), 159.18 (CO; SPR), 136.35 (CS; SPR), 129.92 (CH; SPR), 128.59 (CH; SPR), 122.57 (C; SPR), 106.37 (C; *p*-cymene), 101.68 (C; *p*-cymene), 86.34 (CH; *p*-cymene), 85.49 (CH; *p*-cymene); 81.83 (CH; *p*-cymene); 79.89 (CH; *p*-cymene), 60.78 (CH_2_O; L5); 60.13 (CH; SPR), 56.58 (CH_3_O; SPR), 53.09 (CH_2_; SPR), 47.92 (CH_2_; SPR), 45.59 (CH_2_; L5), 44.83 (CH_2_; L5); 41.10 (CH_2_(NH); SPR), 29.95 (CH; *p*-cymene), 27.94 (CH_2_(NH); L5), 25.76 (CH_2_; SPR), 23.30 (CH_2_; SPR), 22.13 (CH_3_; *p*-cymene), 21.48 (CH_3_; *p*-cymene), 17.85 (CH_3_; *p*-cymene), 13.12 (CH_3_; SPR). UV-Vis λ_max_ (nm)_:_ 274, 340, 378.

Several attempts to grow single crystals of complexes **1a- 5a** for X-ray diffraction analysis were undertaken but remained unsuccessful.

The metal complexes **1a**, **3a** and **5a** were chosen for the solubility, dissolution, permeation and cytotoxicity studies described below. This is due to their favourable yields (>90%) and physical state (powder). The products, in fact, needed to be accurately weighed for these studies; which made powder more suitable than oil residues.

### Solubility Studies of free SPR and SPR in complexes 1a, 3a and 5a

Solubility studies were conducted in Millipore water, PBS pH 6.8, PBS pH 7.4 and methanol by adapting published methods^69,70^. A known amount of each compound was dissolved in a known quantity of each solvent (twenty replicates) and the resulting solutions were placed in an orbital shaker incubator LM-530 (Lasec, South Africa) at a speed of 25 rpm for 24 hours at 37 °C. The resulting solutions were filtered. The amount of SPR dissolved in each solvent was then quantified using a UV-Vis calibration curve.

### Dissolution studies of free SPR and SPR in complexes 1a, 3a and 5a

Dissolution studies of commercially available sulpiride (50 mg Eglonyl® capsules), 106 mg complex 1a, 95.4 mg complex 3a and 99.2 mg complex 5a were performed as per USP guidelines. The UV-Vis calibration curve for SPR was used to ensure that all metal complexes had an equivalent SPR amount (50 mg). All experiments were performed in triplicate. The samples were prepared by inserting them into empty capsules equivalent in size and shape to Eglonyl® capsules. DT 700 dissolution tester (Erweka, Germany) in paddle mode was used. The dissolution medium was PBS (900 mL) at different pH conditions (1.5, 6.8 and 7.4) to simulate different parts of the gastrointestinal tract. The stirring rate was 100 rpm and the temperature was kept at 37 ± 0.5 °C for the duration of the experiment (24 hours). A stainless mesh ring was placed into the dissolution, below the paddle, in order to minimise sample floating. Sampling (5 mL) was done with replacement with the dissolution medium at 0.5, 1, 1.5, 2, 3, 4, 5, 6, 7, 8, 9, 10, 12 and 24 hours for all pH values investigated. Withdrawn samples were assayed for dissolved SPR using a UV-Vis calibration curve.

### Permeation studies of free SPR and SPR in complexes 1a, 3a and 5a

*Ex vivo* permeation studies were performed to evaluate the comparative intestinal absorption of free SPR against SPR in metal complexes. This study was approved by the Animal Ethics Screening Committee (AESC) of the University of the Witwatersrand, Johannesburg (Ref: Reference: Gretta Mbitsi-Ibouily 14-11-14 O) and all experiments were performed in accordance with guidelines and regulations prescribed by the AESC. Intestinal tissue from euthanised pigs was obtained from at the Central Animal Service (CAS) of the University of the Witwatersrand. Porcine small intestines were surgically removed, transported to the laboratory where they were cleaned, and the exogenous tissues and subcutaneous layers carefully removed. They were then stored at −80 °C for further use. On the day of the experiment, intestinal tissues were thawed at room temperature, cut in pieces prior to use and mounted on vertical Franz diffusion cells (United Scientific, South Africa). Each Franz diffusion cell had a membrane area of 1.77 cm^2^ exposed and a 12 mL receptor chamber capacity. The tissue membranes were mounted between the donor and receptor compartment with the apical side facing the donor compartment and the basolateral side facing the receptor medium, which was filled with PBS, pH 7.4. Each sample was done in triplicate. Samples consisted of 5 mg SPR, 10.6 mg complex 1a, 9.5 mg complex 3a and 9.9 mg complex 5a. SPR’s UV-Vis calibration curve was used to ensure that all metal complexes contained equivalent amounts of SPR (5 mg). Each sample was applied to the donor compartment and 3 mL PBS, pH 6.8, was added. Temperature was kept at 37 ± 0.5 °C. Samples of 0.1 mL were withdrawn at intervals 0.5, 1, 1.5, 2, 3, 4, 5, 6, 7, 8, 12 and 24 hours, and replaced with the same volume of buffer solution. Withdrawn samples were assayed for dissolved SPR using a UV-Vis calibration curve.

The cumulative amount of SPR permeated across the membrane and the flux (J) values across the membrane were calculated in accordance with the formulas below:

3 $${\rm{Cumulative}}\,{\rm{amount}}\,{\rm{of}}\,{\rm{drug}}\,{\rm{permeated}}=\frac{{\boldsymbol{Q}}}{{\boldsymbol{A}}}(\mu g.{{\rm{cm}}}^{-2})$$

Where Q = amount of substance crossing the membrane (µg)

A = membrane area exposed (cm^2^)

4 $${\rm{J}}=\frac{{\boldsymbol{Q}}}{{\boldsymbol{At}}}(\mu g.{{\rm{cm}}}^{-2}.{{\rm{h}}}^{-1})$$

Where Q = amount of substance crossing the membrane (µg)

A = membrane area exposed (cm^2^)

t = exposure time (h)

In *ex vivo* studies, confirmation of tissue integrity is essential, since any compromised tissue integrity during handling will result in inaccurate permeation results^71^. Ionic conductivity as a measure of the porcine intestinal tissue integrity was determined using a SevenMulti S40 pH/electrical conductivity meter (Mettler-Toledo, Zurich, Switzerland) prior to (t_0_) the experimental procedure and at t_8_, t_12_ and t_24_. The FTIR spectrum of the intestinal tissue was also collected and at t_0_ t_8_, t_12_ and t_24_^71,72^.

### *In vitro* toxicity testing of free SPR and SPR in complexes 1a, 3a and 5a using Caco-2 cell line

The small intestinal lumen surface area is lined with an epithelial cell monolayer, which isolates the systemic circulation from the intestinal lumen. This epithelial monolayer prevents the invasion of bacteria and toxic compounds from the gastrointestinal tract. Intestinal epithelial cells can be disturbed or damaged by either toxic chemical compounds or toxicity generated during digestion. Disturbance or damage in the intestinal epithelial tissues may result in the weakening of its protective role. Therefore, the possible cytotoxicity of free sulpiride and sulpiride in complexes 1a, 3a and 5a was investigated using Caco-2 intestinal cell line (Cellonex, South Africa), a human cell line derived from a colon adenocarcinoma. This cell line was selected due to its wide use in assays involving drug absorption following oral administration, as well as its similar characteristics to those of the absorptive intestinal epithelium^73–75^.

#### Caco-2 cell line culturing

Caco-2 cells (Cellonex, South Africa) were grown in culture flasks containing solution Dulbecco’s Modified Eagle Medium (DMEM), supplemented with 20% fetal bovine serum with 4.0 mM l-glutamine and sodium pyruvate, with added 1 mL penicillin/streptomycin (Sigma- Aldrich; St. Louise, MO, USA). Cells were maintained in an incubator (RS Biotech Galaxy, Irvine, UK) under humidified atmosphere of 5% CO_2_ at 37 °C during cell growth. The cell medium was replaced every 2 to 3 days. Cells were grown until they reached 60–90% confluence before cytotoxicity tests were conducted.

#### Cell counting using trypan blue solution assay and a haemocytometer

When 60–90% confluence was reached, the medium was discarded from the cultured flask, followed by addition of trypsin-EDTA (3 mL) and incubation for 5 minutes to detach the cells. The cultured flask was scrapped to ensure detachment of all cells. The incubated solution was centrifuged at 1500 g for 5 minutes. The supernatant was discarded, and cells were resuspended in fresh medium 1 mL). Trypan blue solution (100 μL) was added to the suspended cells (100 μL). The disposable haemocytometer chamber was filled with a mixture of trypan blue solution added to the suspended cells. Light microscopy (Olympus CKS53 microscope, Olympus, Japan) was used to examine the chamber for cell counting. Trypan blue solution only stains dead cells. By counting unstained cell, the number of living cells in the sample was determined.

#### *In vitro* cytotoxicity evaluation using methyl thiazolyl tetrazolium (MTT) assay

Cytotoxicity of the of free SPR and SPR in complexes 1a, 3a and 5a in Caco-2 cell line was evaluated using the MTT assay. Multi well plates (96) were seeded with Caco-2 cells at a density of 2 × 10^4^ cells/well and incubated for 24 hours. After culturing the cells in 96 well plates for 24 hours in the incubator (RS Biotech Galaxy, Irvine, UK) under humidified atmospheric conditions of 5% CO_2_ at 37 °C, the culture was removed from the incubator into a laminar flow unit. Thereafter, different concentrations of prepared SPR and complexes 1a, 3a and 5a solutions (50, 100, 250, 500 and 1000 mg/L SPR) of equal volumes were added to the initial culture media. The cells were incubated for further 24 hours at 37 °C. At the end of the 24-hour incubation, 10 μL of MTT solution was added to the wells, and the 96 well plate was incubated for 4 hours to allow the conversion of MTT to formazan by mitochondrial dehydrogenase. Following the 4-hour incubation period, the medium was removed from the wells. The formazan formed crystals were dissolved by adding DMSO solution (100 µL). The plates were placed in an orbital shaker overnight. Absorbance was measured at a wavelength of 570 nm. The background absorbance of the multi well plates was measured at 690 nm and was subtracted from the 570nm measurement. The resulting measurements were presented as percentage cell viability (mean ± standard deviation). Equation (5) was used to calculate the percentage cell viability:

5 $${\rm{Percentage}}\,{\rm{cell}}\,{\rm{viability}}=\frac{{\boldsymbol{Mean}}\,{\boldsymbol{absorbance}}\,{\boldsymbol{at}}\,{\boldsymbol{each}}\,{\boldsymbol{concentration}}}{{\boldsymbol{Mean}}\,{\boldsymbol{absorbance}}\,{\boldsymbol{of}}\,{\boldsymbol{control}}}\times 100$$

## Supplementary information

Supplementary file (R1)

## Data Availability

The datasets generated during and/or analysed during the current study are available from the corresponding author on reasonable request.
