# Supplementary material for: Synthesis, Characterisation and In Vitro Permeation, Dissolution and Cytotoxic Evaluation of Ruthenium(II)-Liganded Sulpiride and Amino Alcohol
Source: Sci Rep. 2019 Mar 11;9:4146. doi: 10.1038/s41598-019-40538-1 (PMC6412051; doi:10.1038/s41598-019-40538-1)
Supplement: Supplementary file 1 — Supplementary file (R1) [file 41598_2019_40538_MOESM1_ESM.pdf]

*Supplementary Information for*

# Synthesis, Characterisation and *In Vitro* Permeation, Dissolution and Cytotoxic Evaluation of Ruthenium(II)-Liganded Sulpiride and Amino Alcohol

**Gretta C. M'bitsi-Ibouily[1], Thashree Marimuthu[1], Pradeep Kumar[1], Yahya E. Choonara[1], Lisa C. du Toit[1], Priyamvada Pradeep[1], Girish Modi[2], Viness Pillay*,[1]**

[1] Wits Advanced Drug Delivery Platform Research Unit, Department of Pharmacy and Pharmacology, School of Therapeutic Sciences, Faculty of Health Sciences, University of the Witwatersrand, Johannesburg, 7 York Road, Parktown 2193, South Africa

[2] Department of Neurology, Division of Neurosciences, Faculty of Health Sciences, University of the Witwatersrand, Johannesburg, 7 York Road, Parktown 2193, South Africa

* **Author for correspondence:**
Professor Viness Pillay
Email: viness.pillay@wits.ac.za
Tel.: +27-11-717-2274

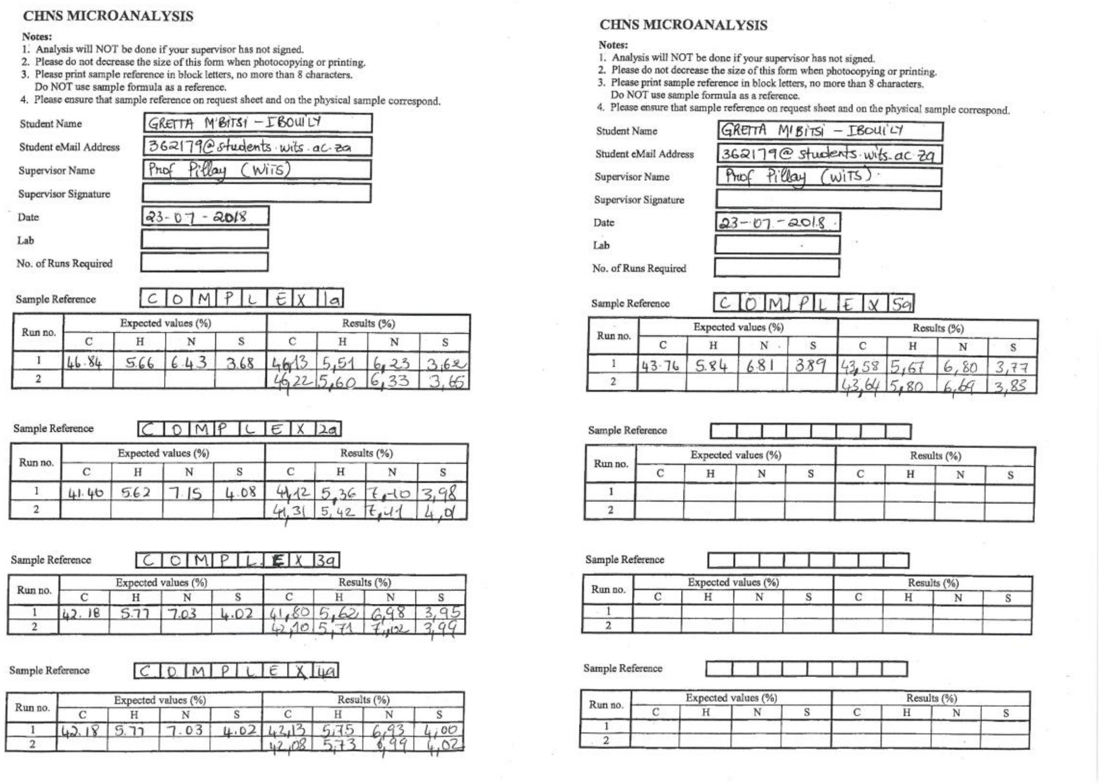

**CHNS MICROANALYSIS**

Notes:
1. Analysis will NOT be done if your supervisor has not signed.
2. Please do not decrease the size of this form when photocopying or printing.
3. Please print sample reference in block letters, no more than 8 characters. Do NOT use sample formula as a reference.
4. Please ensure that sample reference on request sheet and on the physical sample correspond.

Student Name: GRETTA M'BITSI – IBOUI'LY
Student eMail Address: 362179@students.wits.ac.za
Supervisor Name: Prof Pillay (WITS)
Supervisor Signature:
Date: 23-07-2018
Lab:
No. of Runs Required:

Sample Reference: COMPLEX 1a

| Run no. | Expected values (%) | | | | Results (%) | | | |
|---|---|---|---|---|---|---|---|---|
| | C | H | N | S | C | H | N | S |
| 1 | 46.84 | 5.66 | 6.43 | 3.68 | 46,13 | 5,51 | 6,23 | 3,62 |
| 2 | | | | | 46,22 | 5,60 | 6,33 | 3,65 |

Sample Reference: COMPLEX 2a

| Run no. | Expected values (%) | | | | Results (%) | | | |
|---|---|---|---|---|---|---|---|---|
| | C | H | N | S | C | H | N | S |
| 1 | 41.40 | 5.62 | 7.15 | 4.08 | 41,12 | 5,36 | 7,10 | 3,98 |
| 2 | | | | | 41,31 | 5,42 | 7,41 | 4,01 |

Sample Reference: COMPLEX 3a

| Run no. | Expected values (%) | | | | Results (%) | | | |
|---|---|---|---|---|---|---|---|---|
| | C | H | N | S | C | H | N | S |
| 1 | 42.18 | 5.77 | 7.03 | 4.02 | 41,80 | 5,62 | 6,98 | 3,95 |
| 2 | | | | | 42,10 | 5,71 | 7,02 | 3,99 |

Sample Reference: COMPLEX 4a

| Run no. | Expected values (%) | | | | Results (%) | | | |
|---|---|---|---|---|---|---|---|---|
| | C | H | N | S | C | H | N | S |
| 1 | 42.18 | 5.77 | 7.03 | 4.02 | 42,13 | 5,75 | 6,93 | 4,00 |
| 2 | | | | | 42,08 | 5,73 | 6,99 | 4,02 |

**CHNS MICROANALYSIS**

Notes:
1. Analysis will NOT be done if your supervisor has not signed.
2. Please do not decrease the size of this form when photocopying or printing.
3. Please print sample reference in block letters, no more than 8 characters. Do NOT use sample formula as a reference.
4. Please ensure that sample reference on request sheet and on the physical sample correspond.

Student Name: GRETTA M'BITSI – IBOUI'LY
Student eMail Address: 362179@students.wits.ac.za
Supervisor Name: Prof Pillay (WITS)
Supervisor Signature:
Date: 23-07-2018
Lab:
No. of Runs Required:

Sample Reference: COMPLEX 5a

| Run no. | Expected values (%) | | | | Results (%) | | | |
|---|---|---|---|---|---|---|---|---|
| | C | H | N | S | C | H | N | S |
| 1 | 43.76 | 5.84 | 6.81 | 3.89 | 43,58 | 5,67 | 6,80 | 3,77 |
| 2 | | | | | 43,64 | 5,80 | 6,69 | 3,83 |

Sample Reference:

| Run no. | Expected values (%) | | | | Results (%) | | | |
|---|---|---|---|---|---|---|---|---|
| | C | H | N | S | C | H | N | S |
| 1 | | | | | | | | |
| 2 | | | | | | | | |

Sample Reference:

| Run no. | Expected values (%) | | | | Results (%) | | | |
|---|---|---|---|---|---|---|---|---|
| | C | H | N | S | C | H | N | S |
| 1 | | | | | | | | |
| 2 | | | | | | | | |

Sample Reference:

| Run no. | Expected values (%) | | | | Results (%) | | | |
|---|---|---|---|---|---|---|---|---|
| | C | H | N | S | C | H | N | S |
| 1 | | | | | | | | |
| 2 | | | | | | | | |

**Figure S1**. Elemental analysis results of complexes 1a-5a

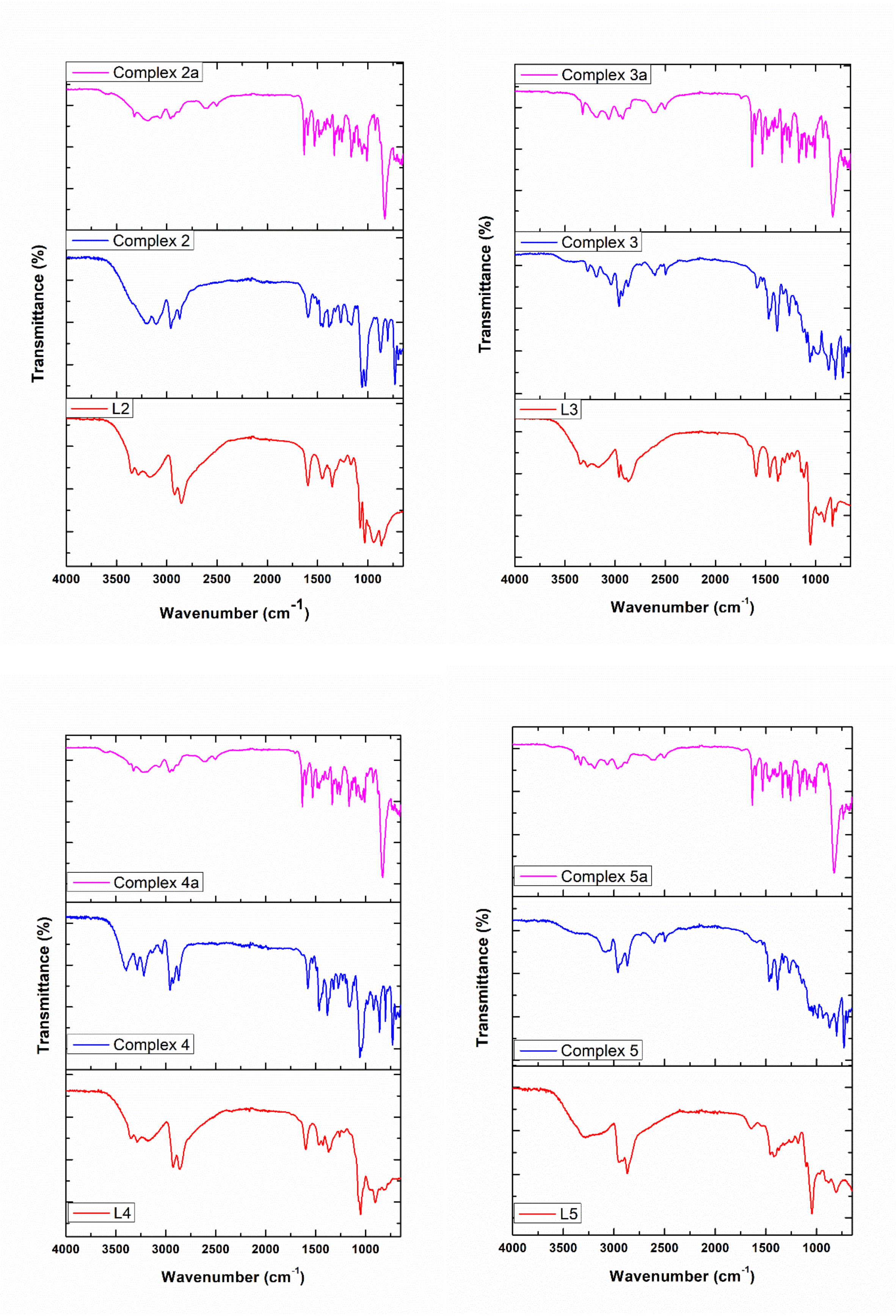


**Figure S2**. FTIR spectra of L2-5, Complexes **2-5** and complexes **2a-5a**

**Table S1**. Selected $^{1}H$ NMR (500 MHz, DMSO-d6) and $^{13}C$ NMR (126 MHz, DMSO-d6) chemical shifts (ppm) of ligands L1-L5, complexes **1-5** and complexes **1a-5a.**

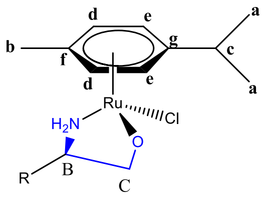


**1** L1, R = $CH_2$-$C_6H_6$
**2** L2, No R
**3** L3, R = $CH_3$

**Complexes 1-3**

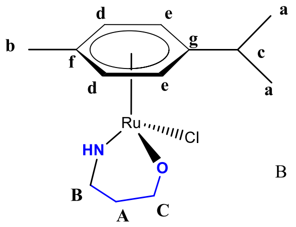


**Complex 4**

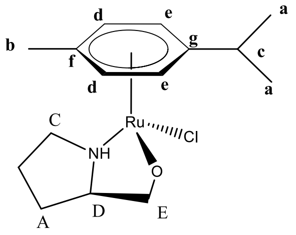


**Complex 5**

| | **H-C=C-H,** ***p*-cymene)** **$^{1}H$ NMR** | **$^{13}C$ NMR** | **$CH_2$-$NH_2$** **$^{1}H$ NMR** | **$^{13}C$ NMR** | **O-$CH_2$** **$^{1}H$ NMR** | **$^{13}C$ NMR** |
|---|---|---|---|---|---|---|
| **L1** | - | - | 3.16 - 2.82(m) | 54.31 | 3.68 (dd, J = 10.7, 3.8 Hz, 1H), 3.45 (dd, J = 10.6, 7.2 Hz, 1H) | 40.96 |
| **L2** | - | - | 2.73 (m) | 43.79 | 3.51 (m) | 63.25 |
| **L3** | - | - | 2.96 (dddd, J = 10.5, 7.8, 6.5, 3.9 Hz) | 48.41 | 3.48 (dd, J = 10.6, 4.0 Hz, 1H), 3.20 (dd, J = 10.6, 7.8 Hz, 1H) | 68.17 |
| **L4** | - | - | 2.78-2.74 (m) | 40.14 | 3.61-3.58 (m) | 61.22 |
| **L5** | - | - | 2.91-2.73 (m) | | 3.49 (s, 1H), 3.29 (dd, J = 10.9, 7.4 Hz, 1H) | |
| **Complex 1** | 5.79 (m, 4H) | 81.12 (CH) 80.73 (CH) | 2.88 (s) | 59.54 | 3.06 (m) | 39.14 |
| **Complex 2** | 5.57 (d, J = 5.8 Hz, 2H), 5.42 (d, J = 5.7 Hz, 2H) | 82.76 (CH) 80.93 (CH) | 3.14 (bs) | 52.28 | 3.75 (bs), | 62.63 |
| **Complex 3** | 5.95 (d, J = 5.3 Hz, 1H), 5.85 (s, 1H,), 5.82 (d, J = 5.4 Hz, 1H), 5.60 (m, 1H) | 81.02 (CH), 80.74 (CH), 80.06 (CH), 78.58 (CH) | 2.88 (bs) | 39.16 | 3.23 (s), | 67.13 |
| **Complex 4** | 5.45 (d, J = 5.9 Hz, 2H), 5.37 (d, J = 5.9 Hz, 2H) | 81.22 (CH), 80.41 (CH) | 3.75 (t, J = 5.1 Hz, 2H) | 47.61 | 3.23 (m) | 60.71 |
| **Complex 5** | 6.01 (d, J = 32.0 Hz, 1H), 5.83 (1H, CH), 5.78 (d, J = 46.1 Hz, 1H) 5.73 (1H, CH) | 81.42 (CH), 81.06 (CH), 80.66 (CH), 79.85 (CH) | 2.20 (s) | 49.40 | 3.29 (s) | 68.61 |
| **Complex 1a** | 5.81 (m, 1H), 5.76 (s, 1H), 5.37 (m, 2H) | 86.35 (CH), 85.50 (CH) | 3.14 (m) | 53.82 | 3.50 (m) | 60.36 |
| **Complex 2a** | 5.61 (m, 2H), 5.44 (m, 2H), | 82.50 (CH), 80.26 (CH) | 2.92 (s) | 51.46 | 3.47 (m) | 61.30 |
| **Complex 3a** | 5.79 (d, J = 15.3 Hz, 4H) | 86.33 (CH), 85.49 (CH) | 2.88 (bs) | 48.45 | 3.23 (s) | 62.17 |
| **Complex 4a** | 5.61 (d, J = 5.5 Hz, 1H), 5.52 (q, J = 5.9, 5.0 Hz), 5.45 (d, J = 6.0 Hz, 1H), 5.41 (d, J = 5.7 Hz, 1H) | 86.29 (CH); 85.44 (CH) | 2.88 (m) | 46.95 | 3.45 (m) | 58.65 |
| **Complex 5a** | 5.79 (d, J = 15.8 Hz, 2H), 5.48 (dd, J = 118.6, 5.5 Hz, 2H) | 86.34 (CH), 85.49 (CH) | 2.98 (s) | 27.94 | 3.12 (s) | 60.78 |

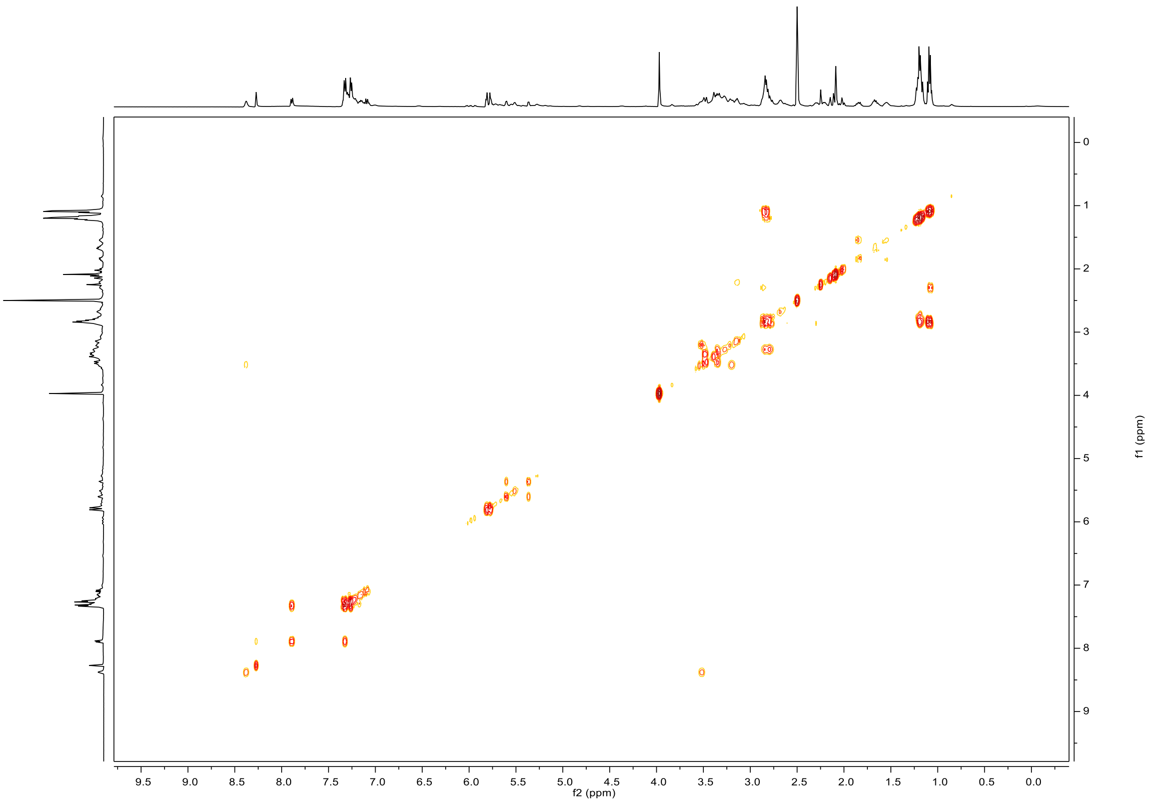


**Figure S3**. COSY (500 MHz, DMSO-d6) of complex **1a**

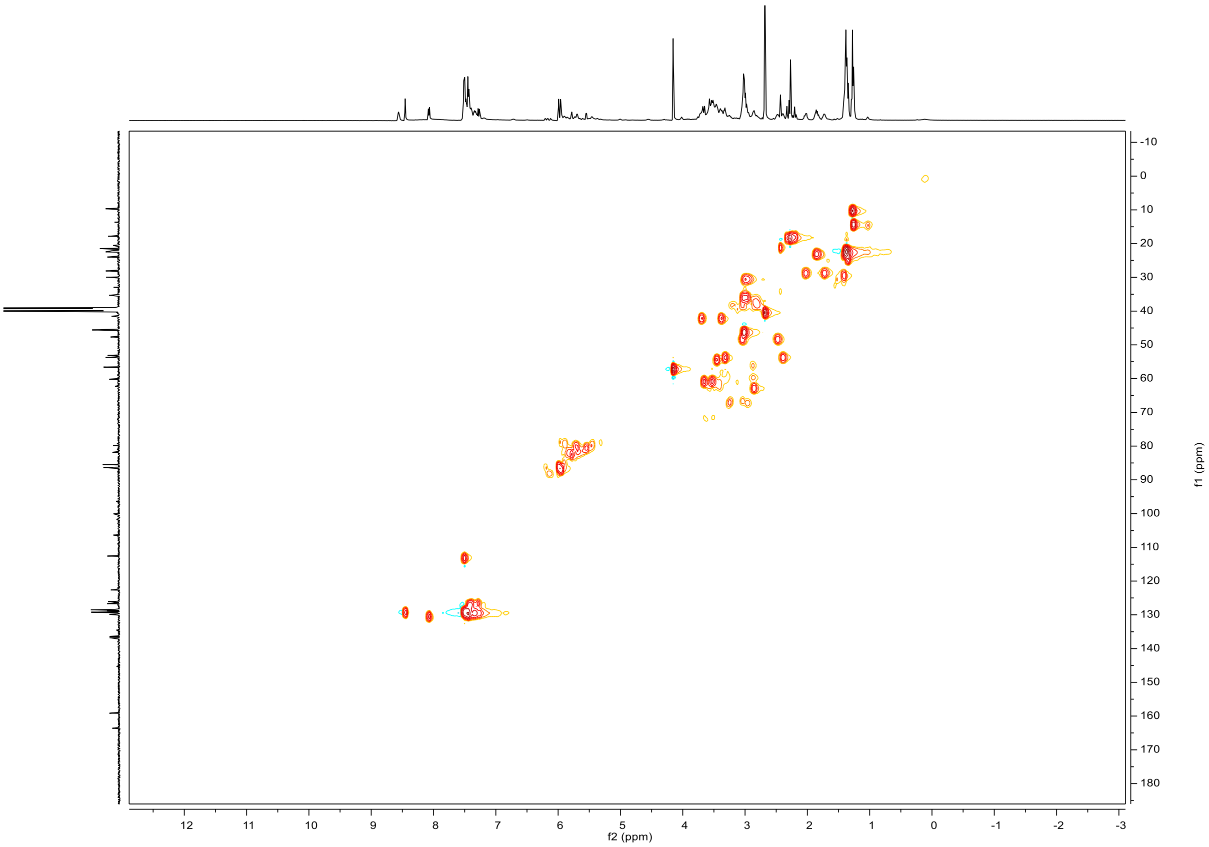


**Figure S4**. HMQC (500 MHz, DMSO-d6) of complex **1a**

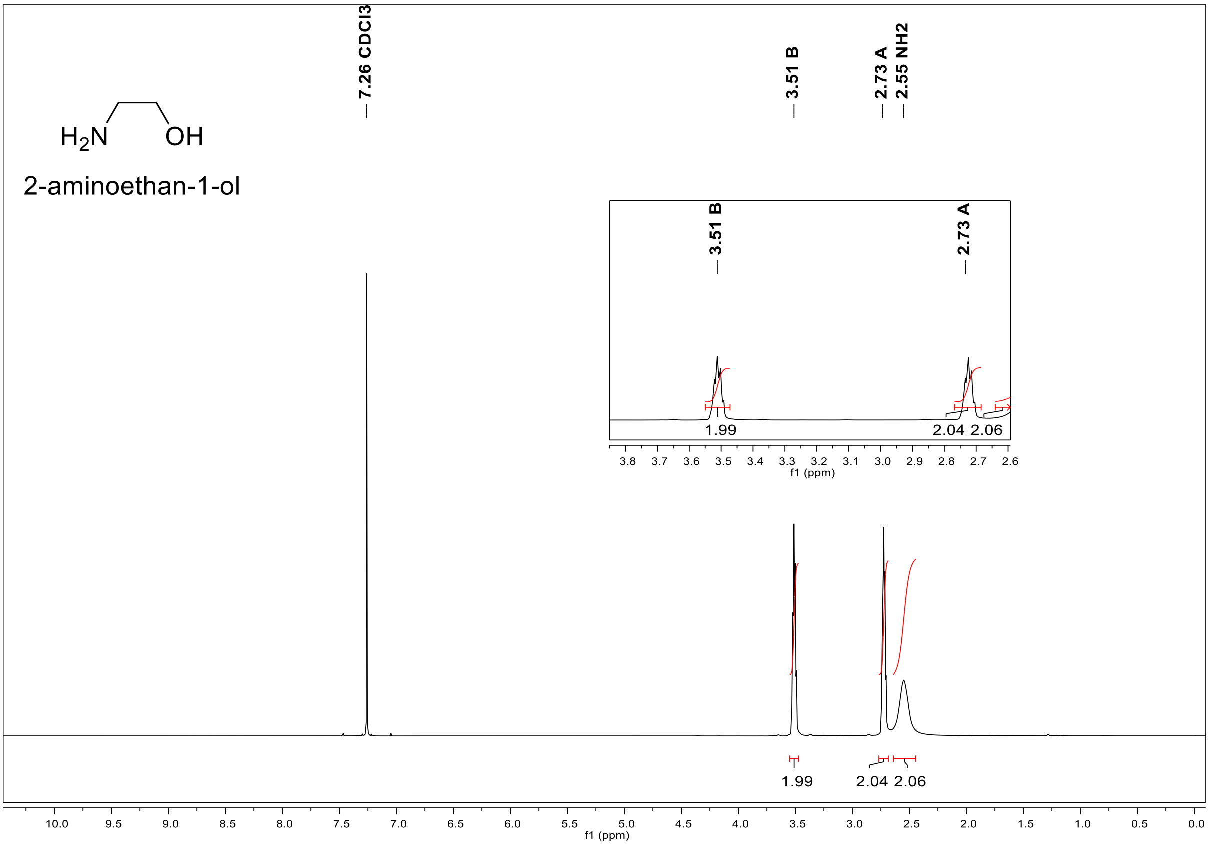


**Figure S5**. $^{1}$H NMR (500 MHz, Chloroform-*d*) of 2-aminoethan-1-ol (L2)

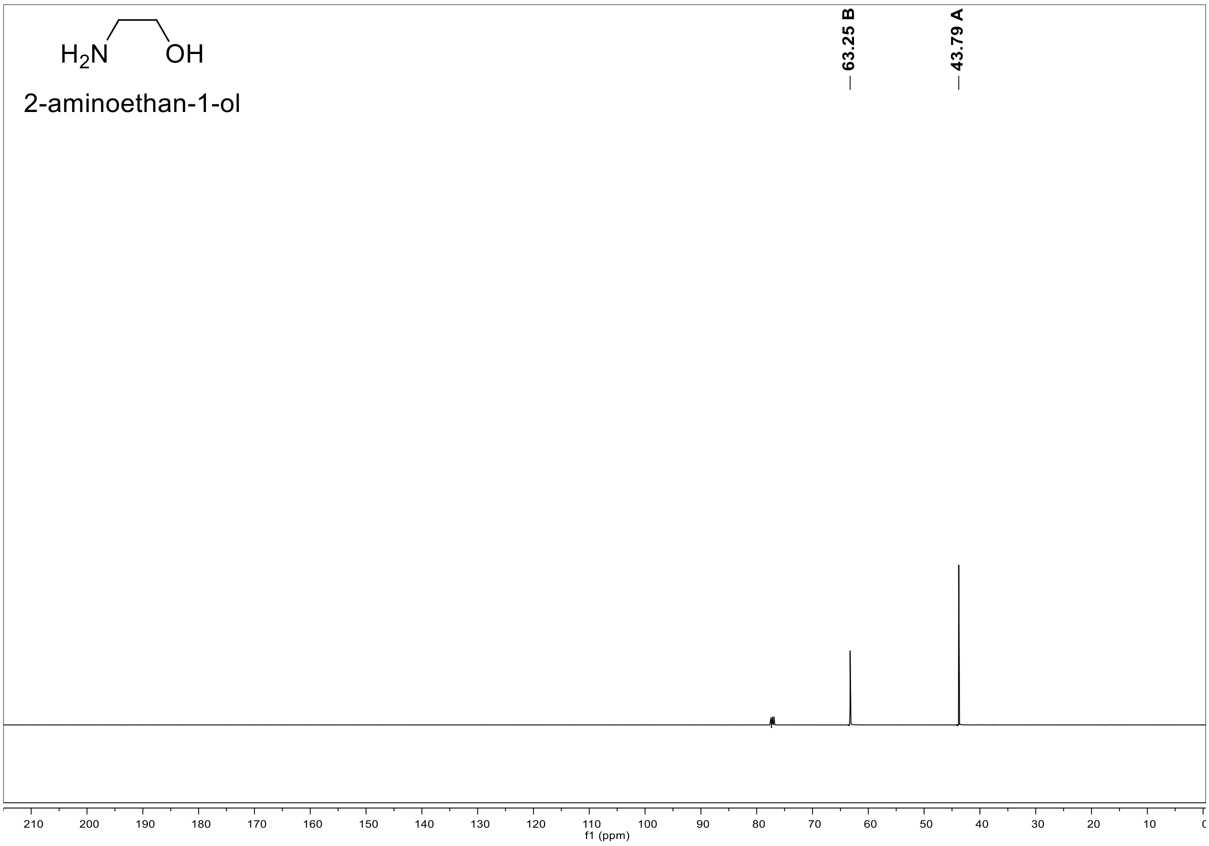


**Figure S6**. $^{13}$C NMR (126 MHz, Chloroform-*d*) of 2-aminoethan-1-ol (L2)

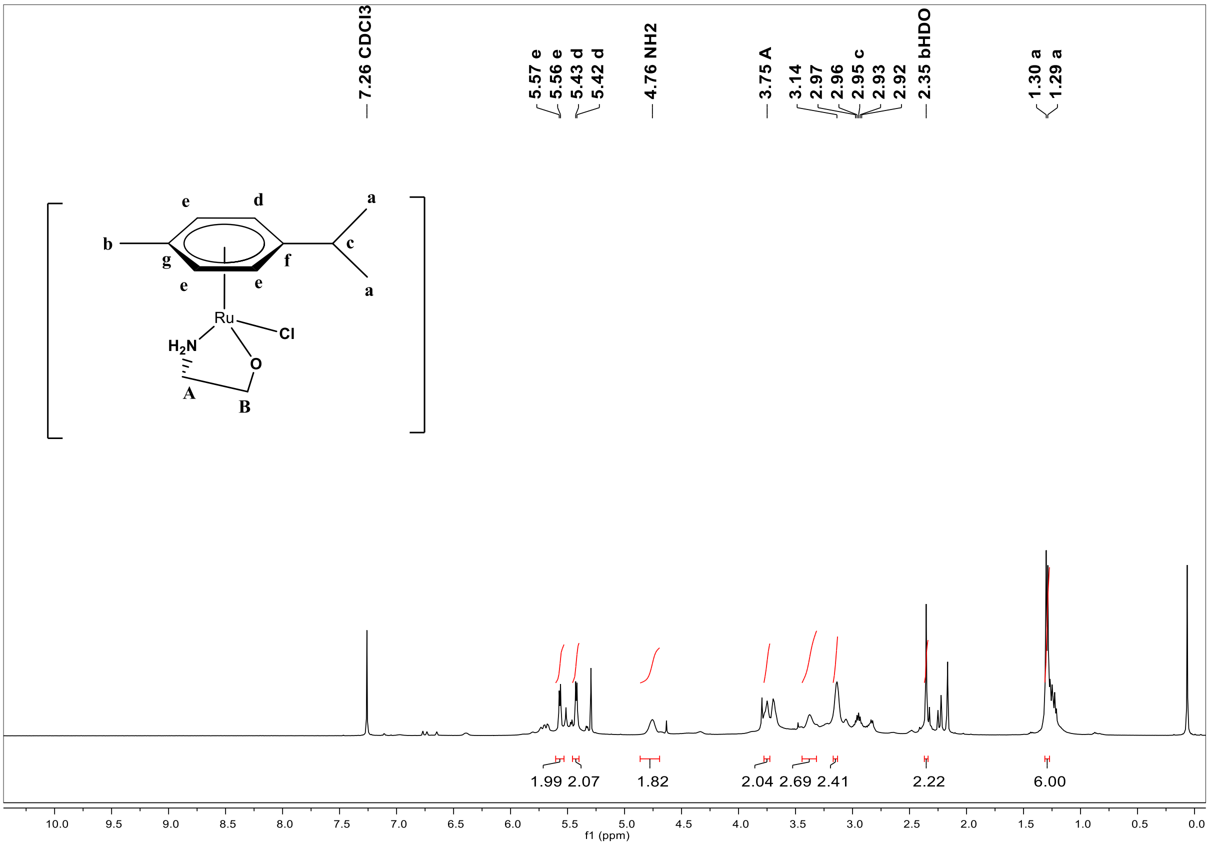


**Figure S7**. $^{1}$H NMR (500 MHz, Chloroform-*d*) of complex **2**

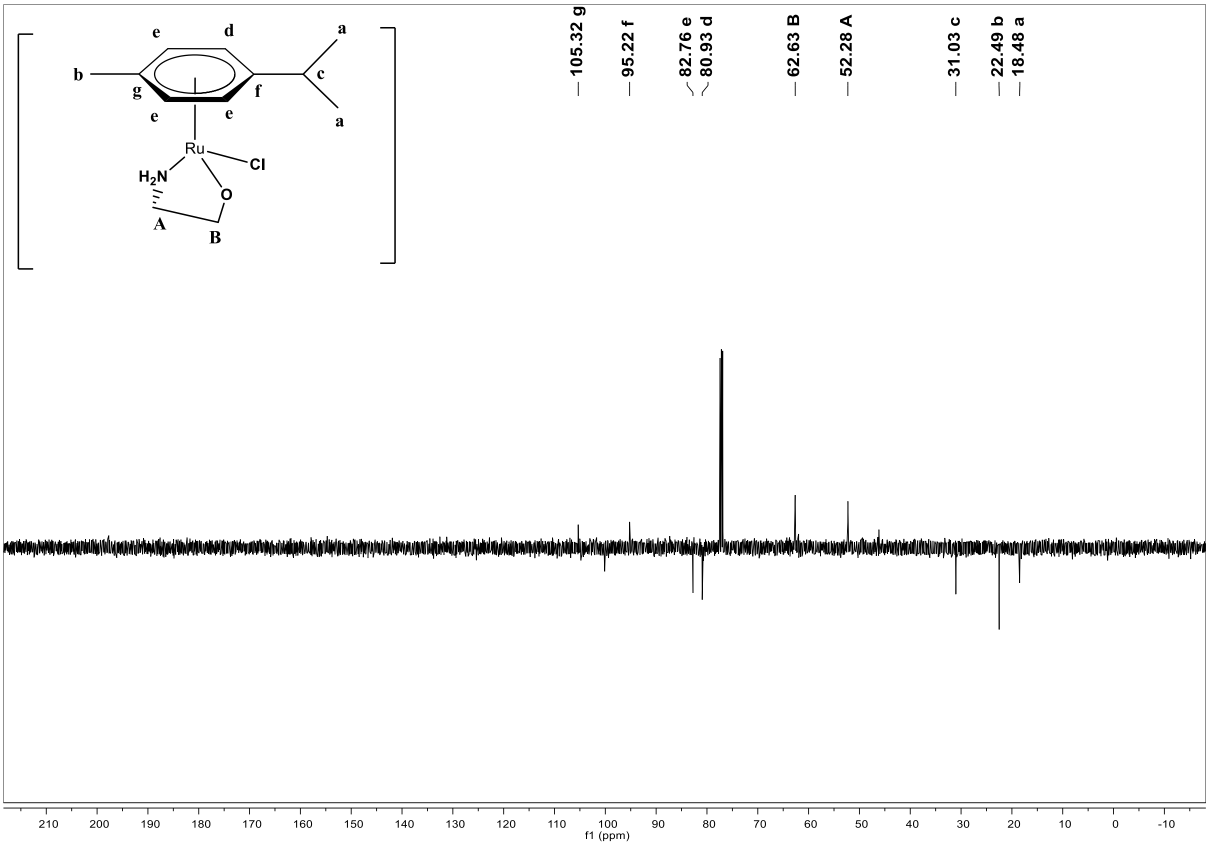


**Figure S8.**$^{13}$C NMR (126 MHz, Chloroform-*d*) of complex **2**

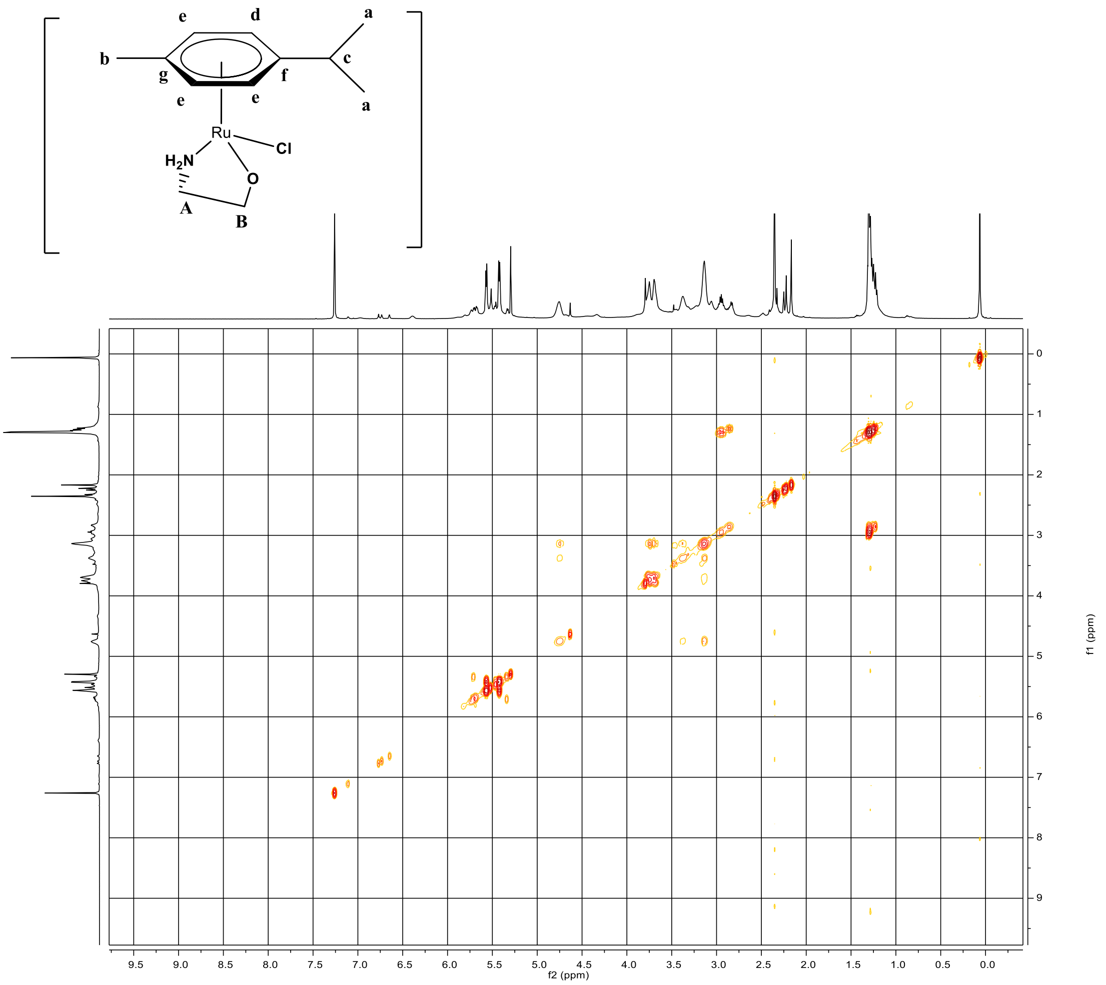


**Figure S9**. COSY (300 MHz, Chloroform-*d*) of complex **2**

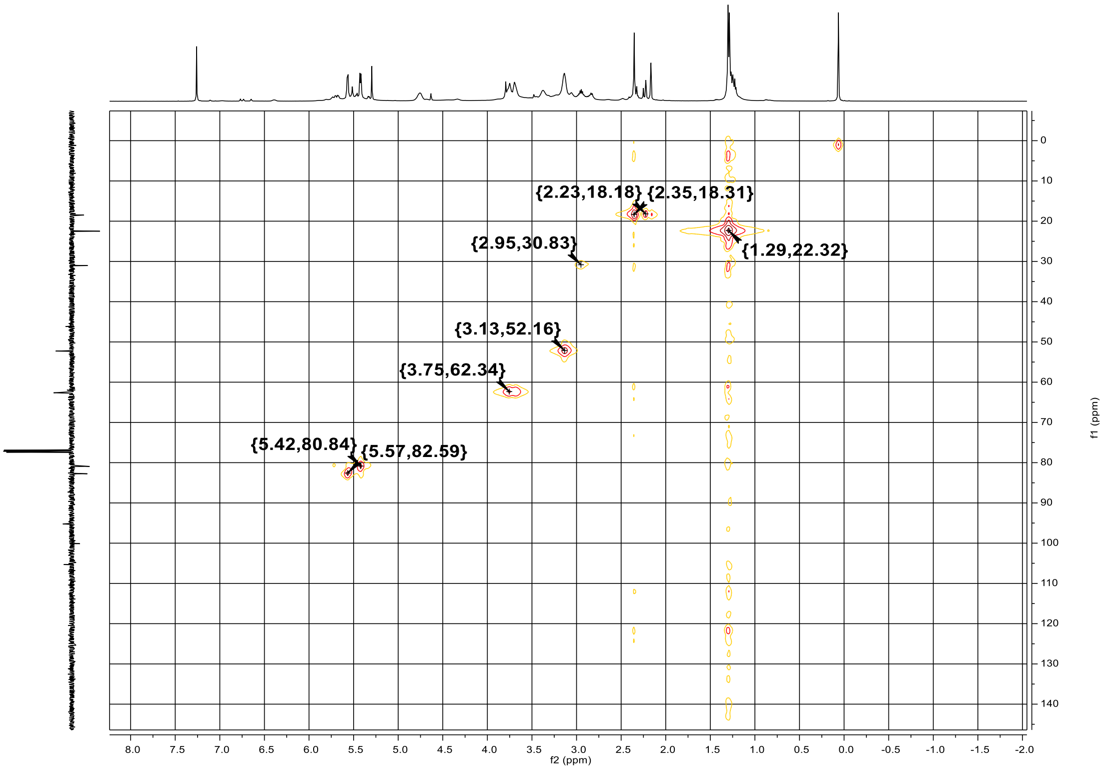


**Figure S10**. HMQC (126 MHz, Chloroform-*d*) of complex **2**

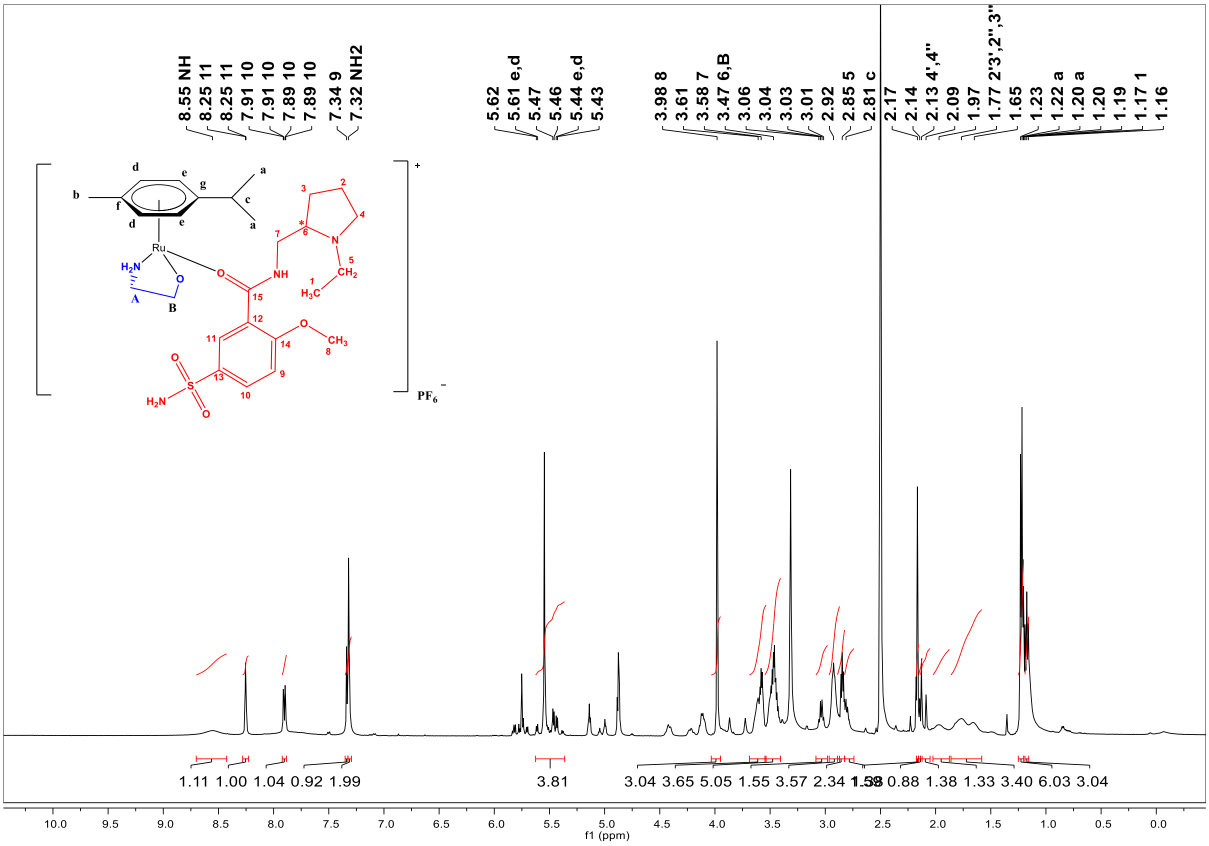

**Figure S11**. $^{1}H$ NMR (126 MHz, DMSO-d6) of complex **2a**

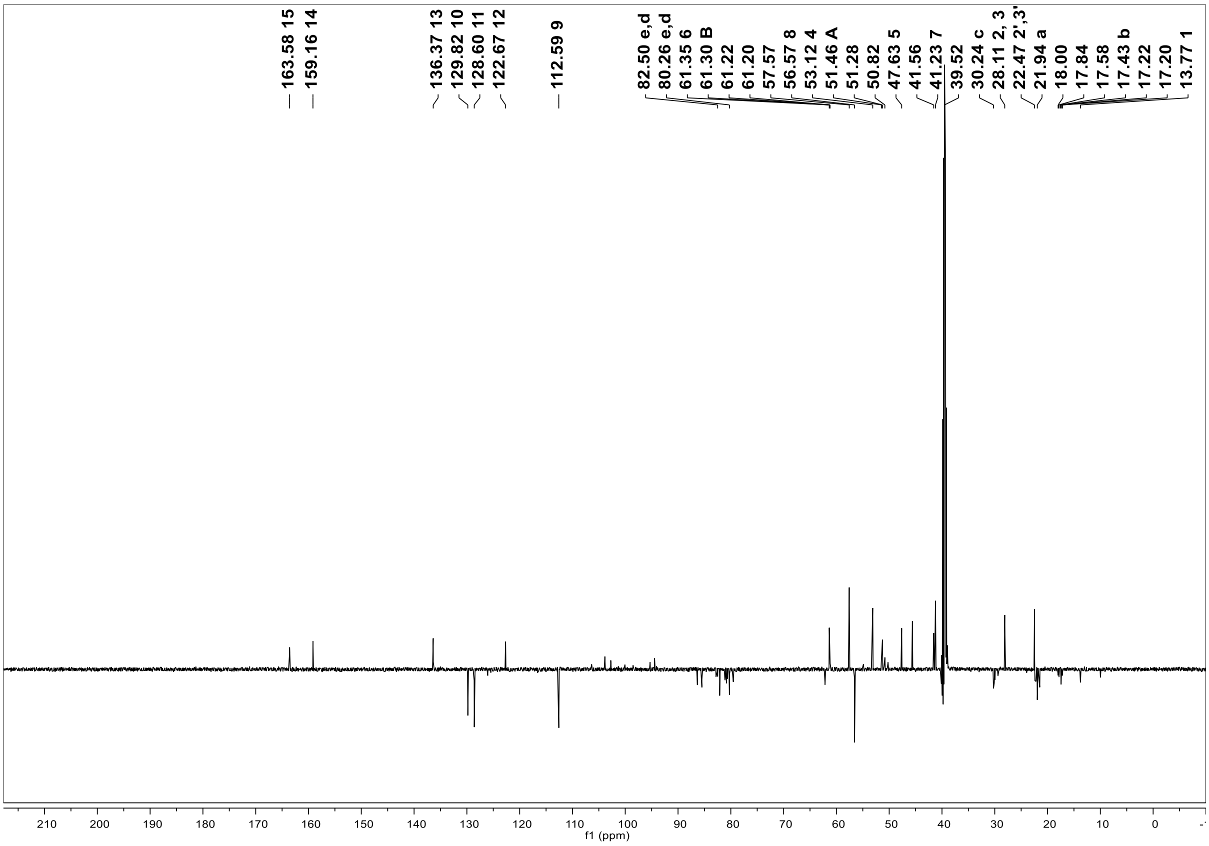

**Figure S12**. $^{13}C$ NMR (126 MHz, DMSO-d6) of complex **2a**

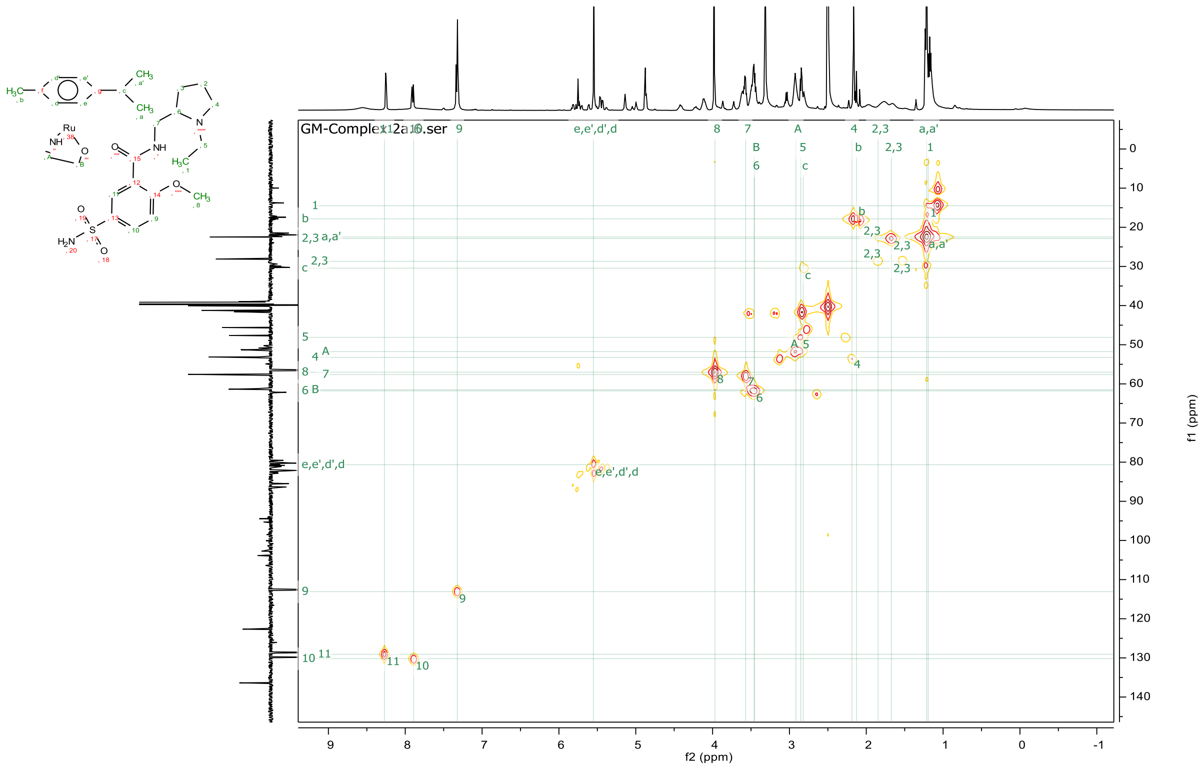

**Figure S13**. HMQC (126 MHz, DMSO-d6) of complex **2a**

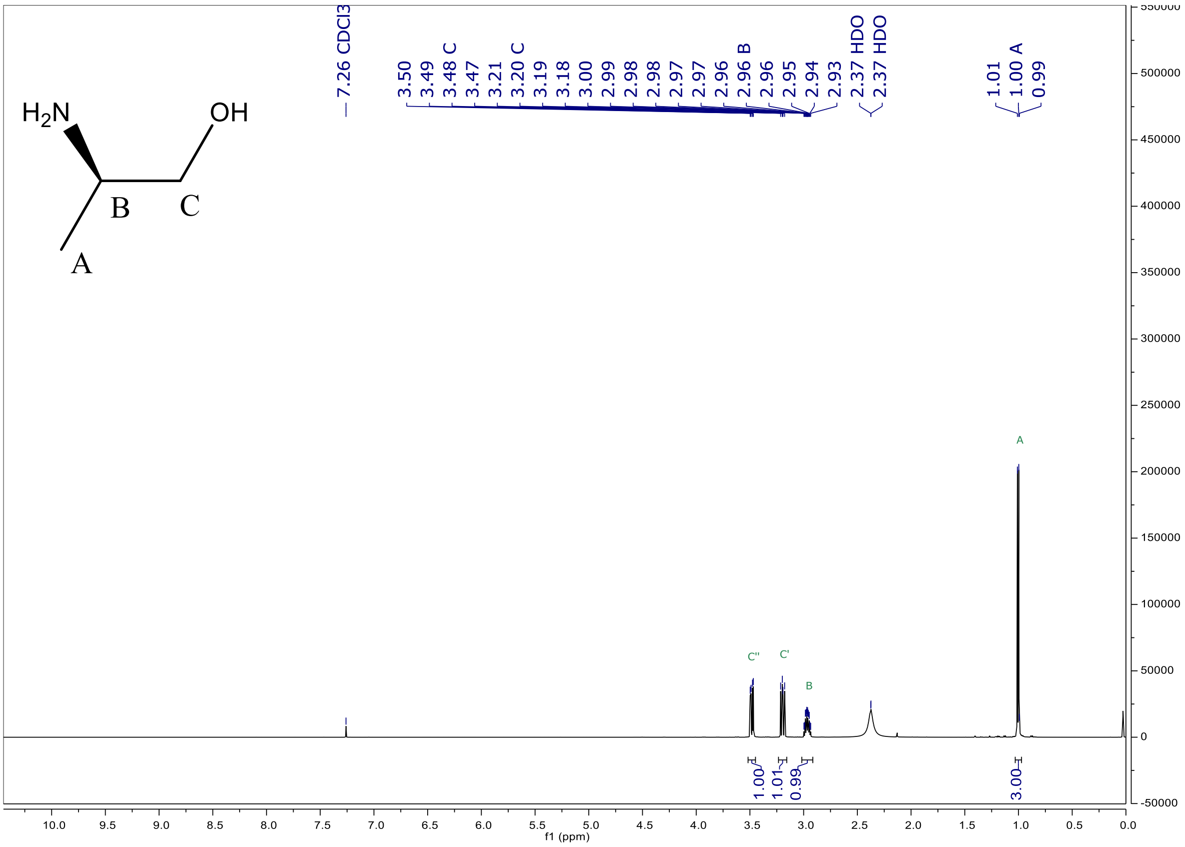

**Figure S14**. $^{1}$H NMR (500 MHz, Chloroform-*d*) of (L3)

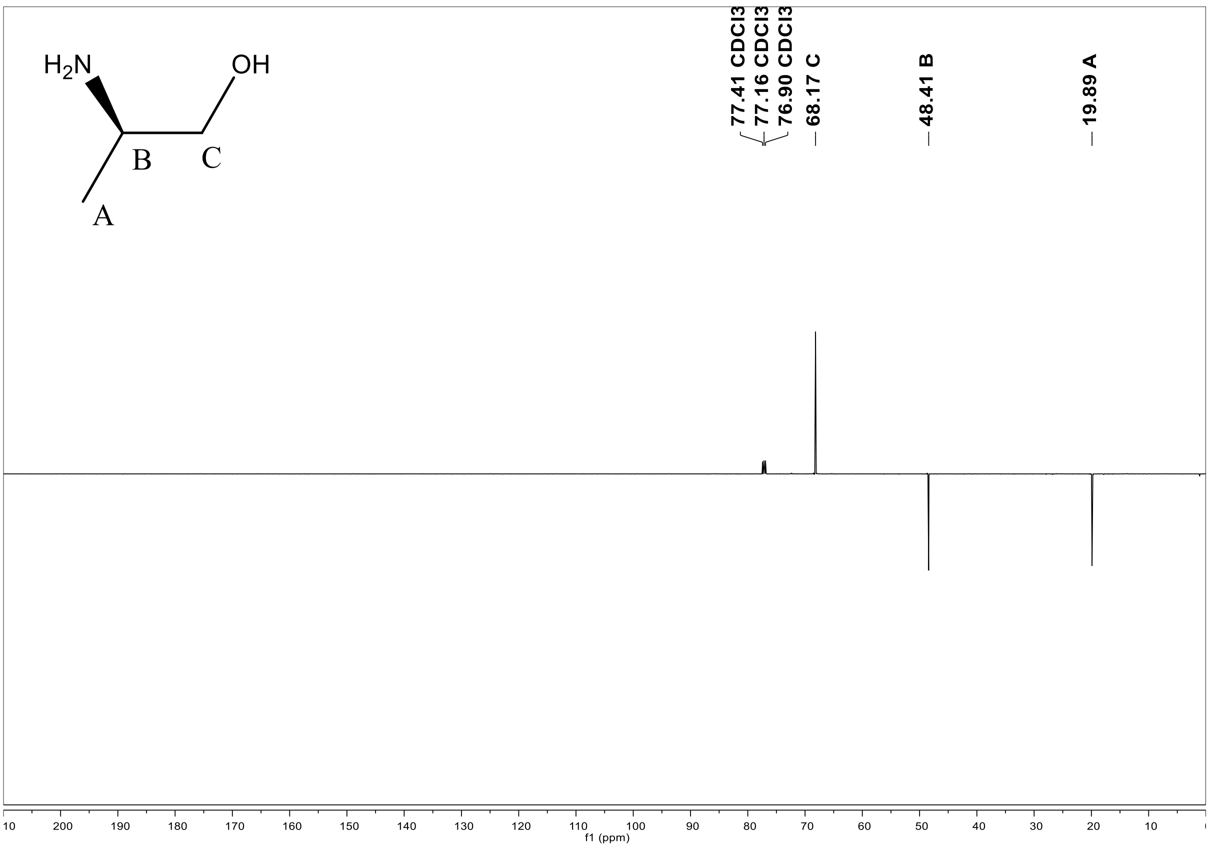


**Figure S15**. $^{13}$C NMR (500 MHz, Chloroform-*d*) of (L3)

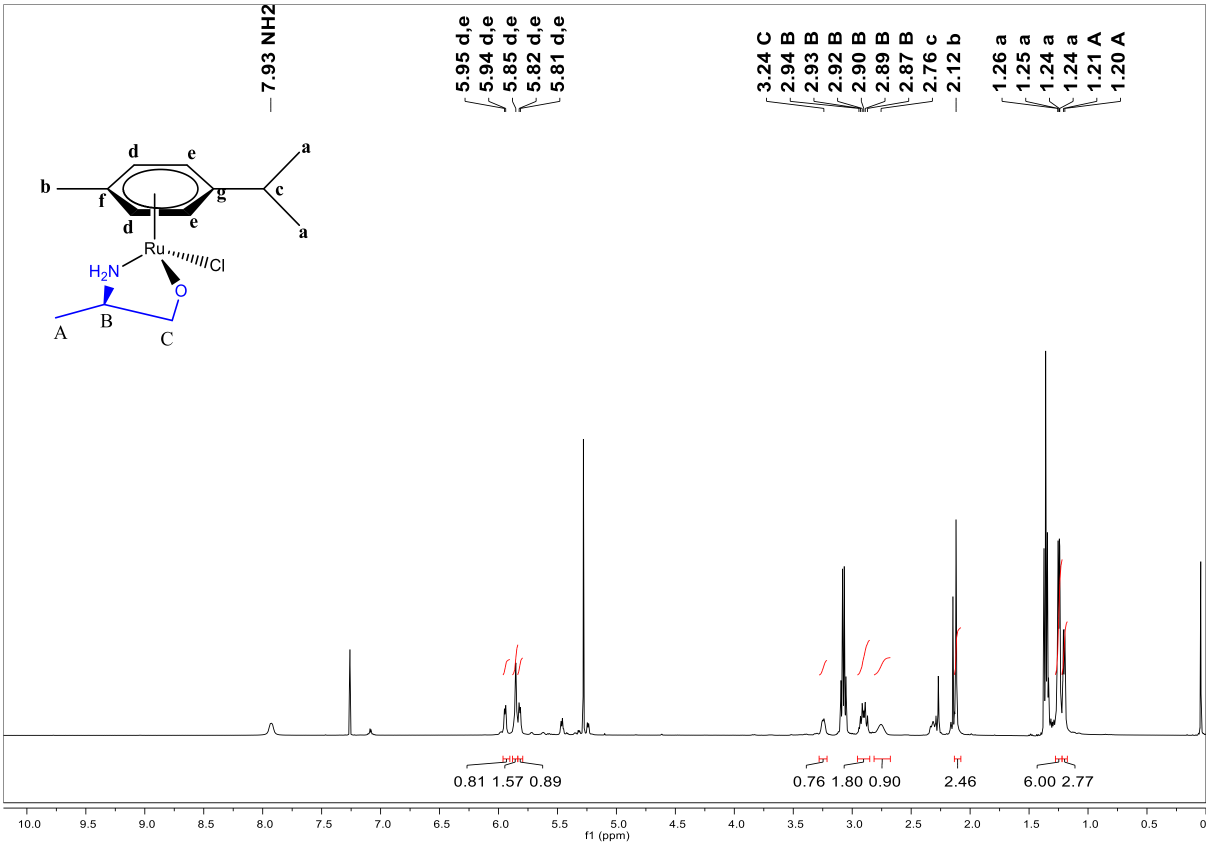


**Figure S16**. $^{1}$H NMR (500 MHz, Chloroform-*d*) of Complex **3**

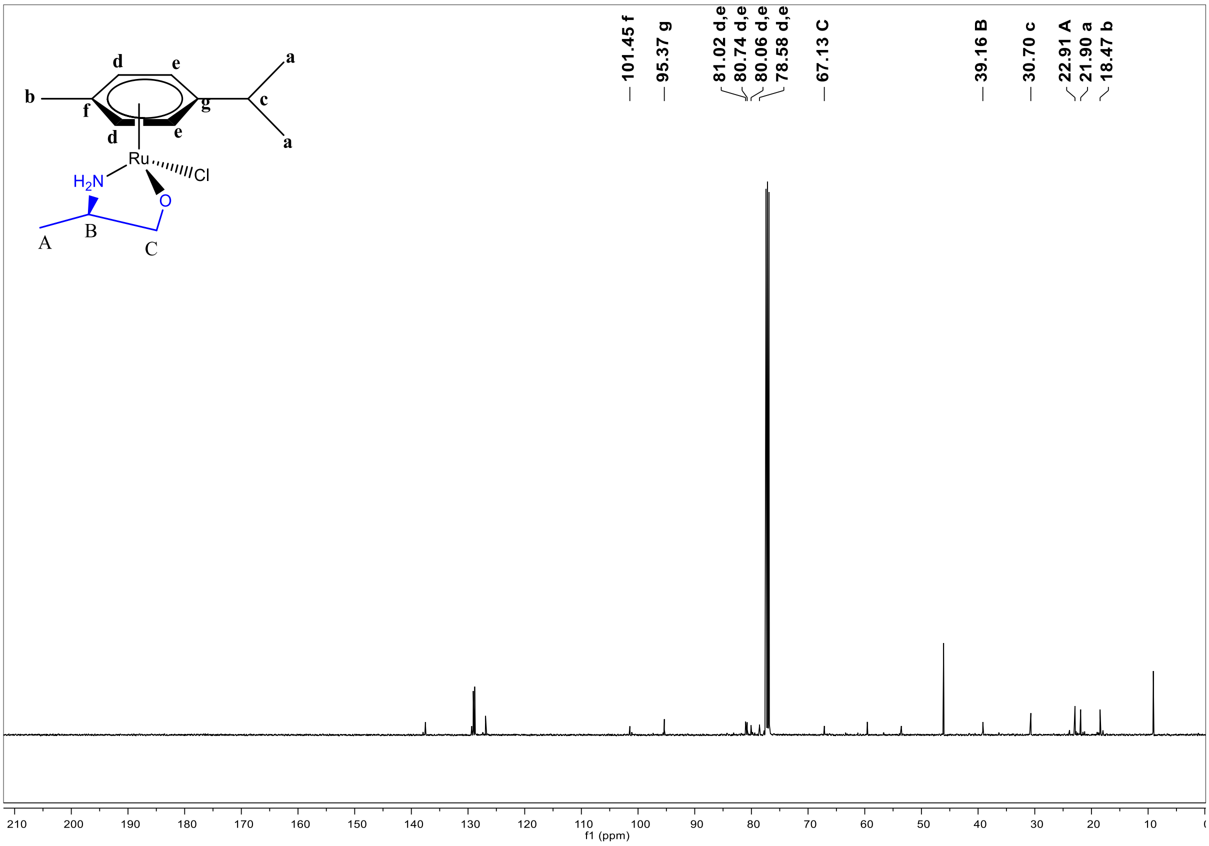


**Figure S17**. $^{13}C$ NMR (500 MHz, Chloroform-*d*) of Complex **3**

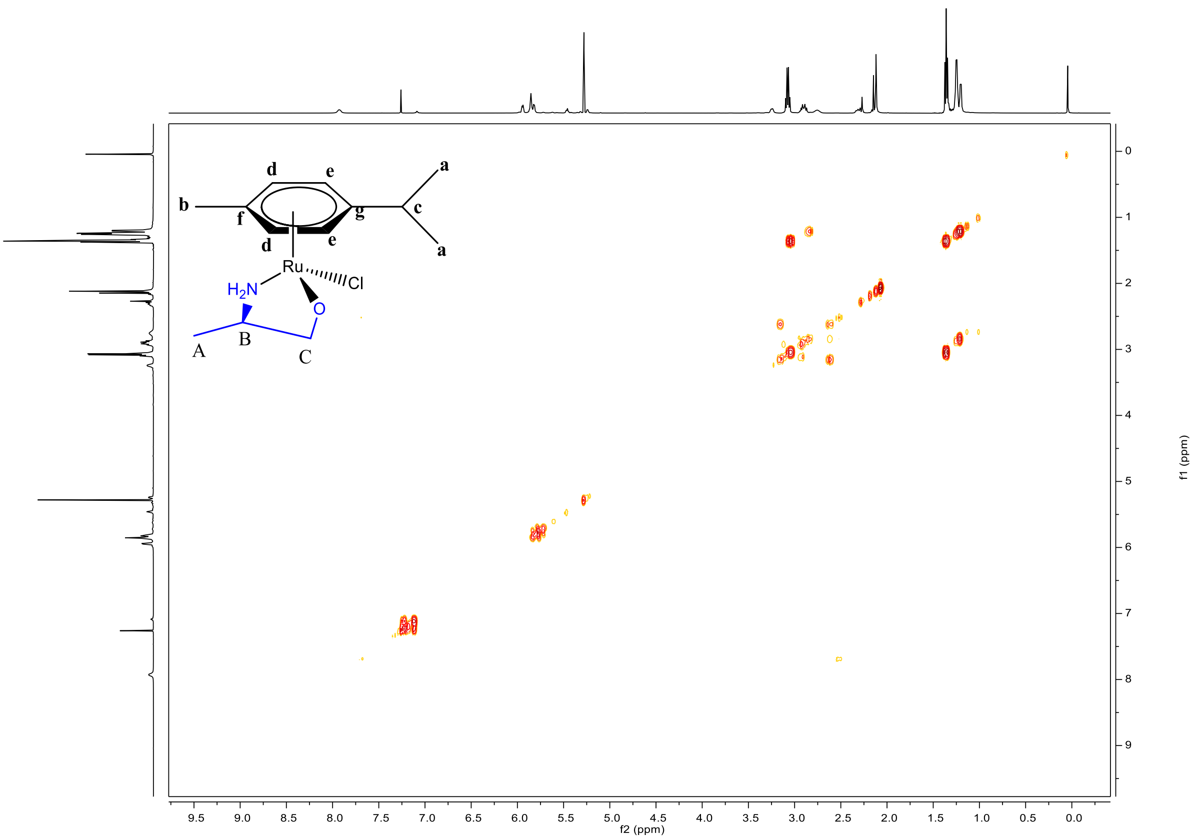


**Figure S18**. COSY NMR (500 MHz, Chloroform-*d*) of Complex **3**

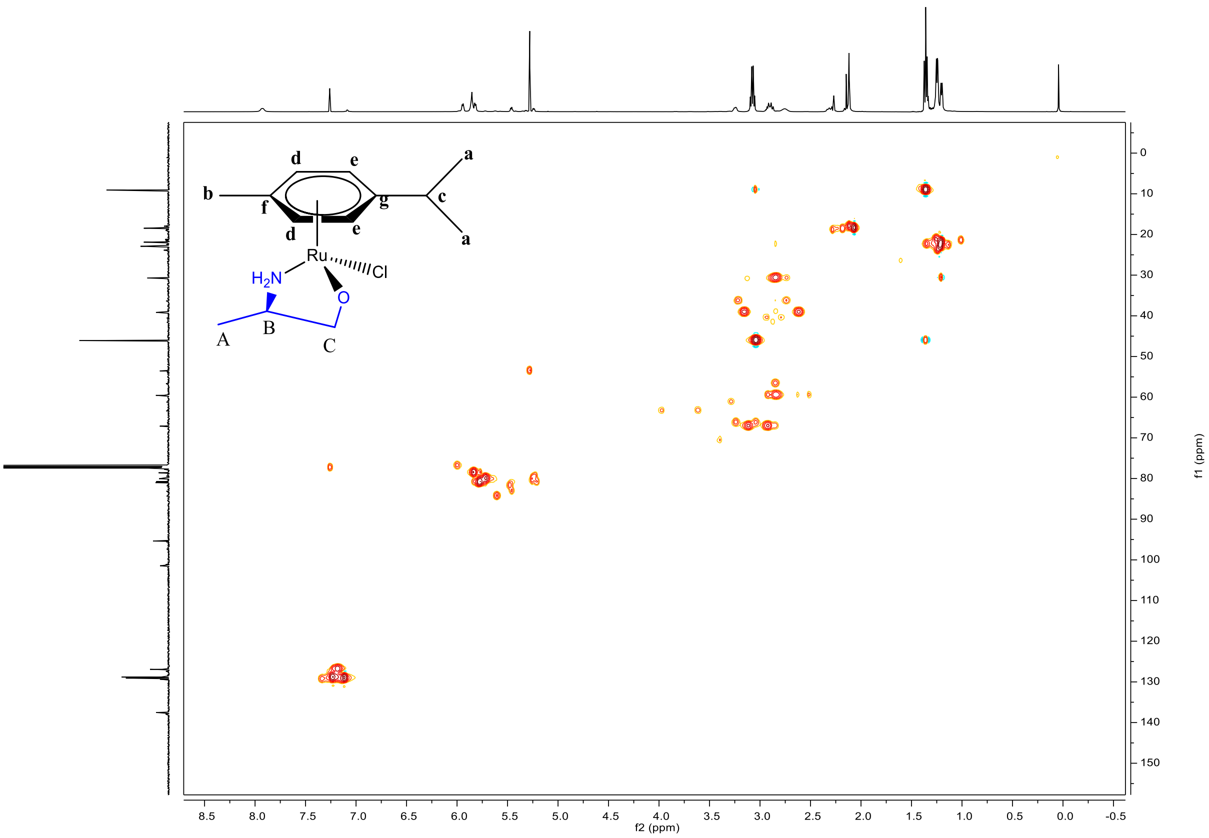


**Figure S19**. HSQC NMR (500 MHz, Chloroform-*d*) of Complex **3**

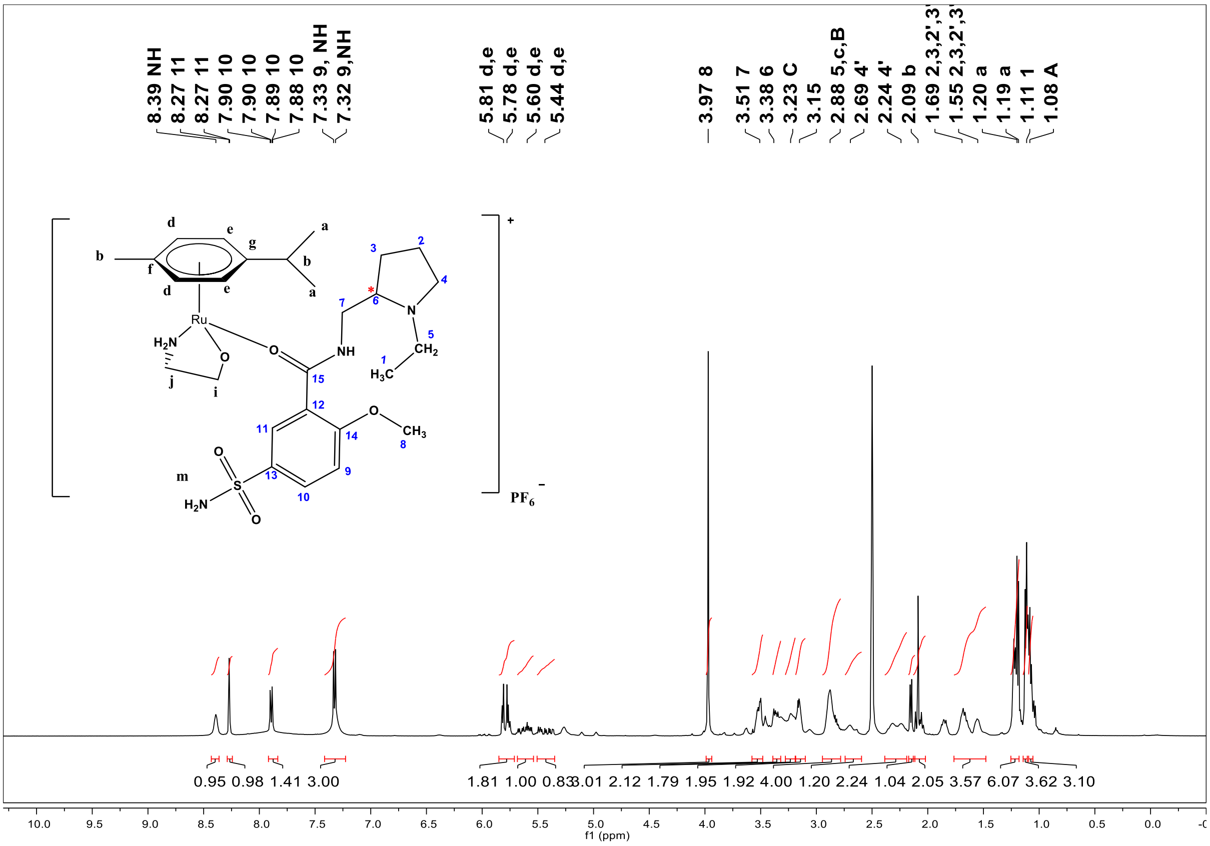


**Figure S20**. $^{1}H$ NMR (500 MHz, DMSO-*d6*) of Complex **3a**

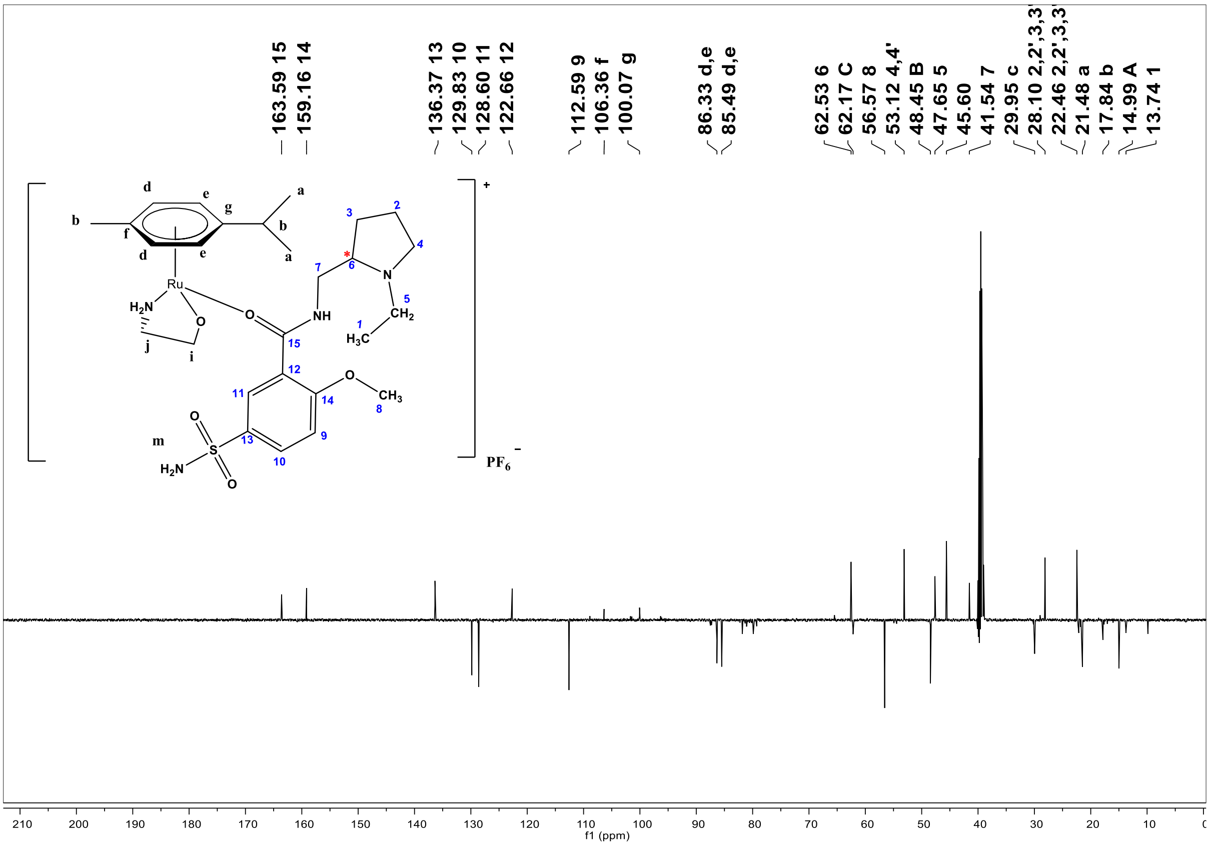


**Figure S21**. $^{13}$C NMR (500 MHz, DMSO-*d6*) of Complex **3a**

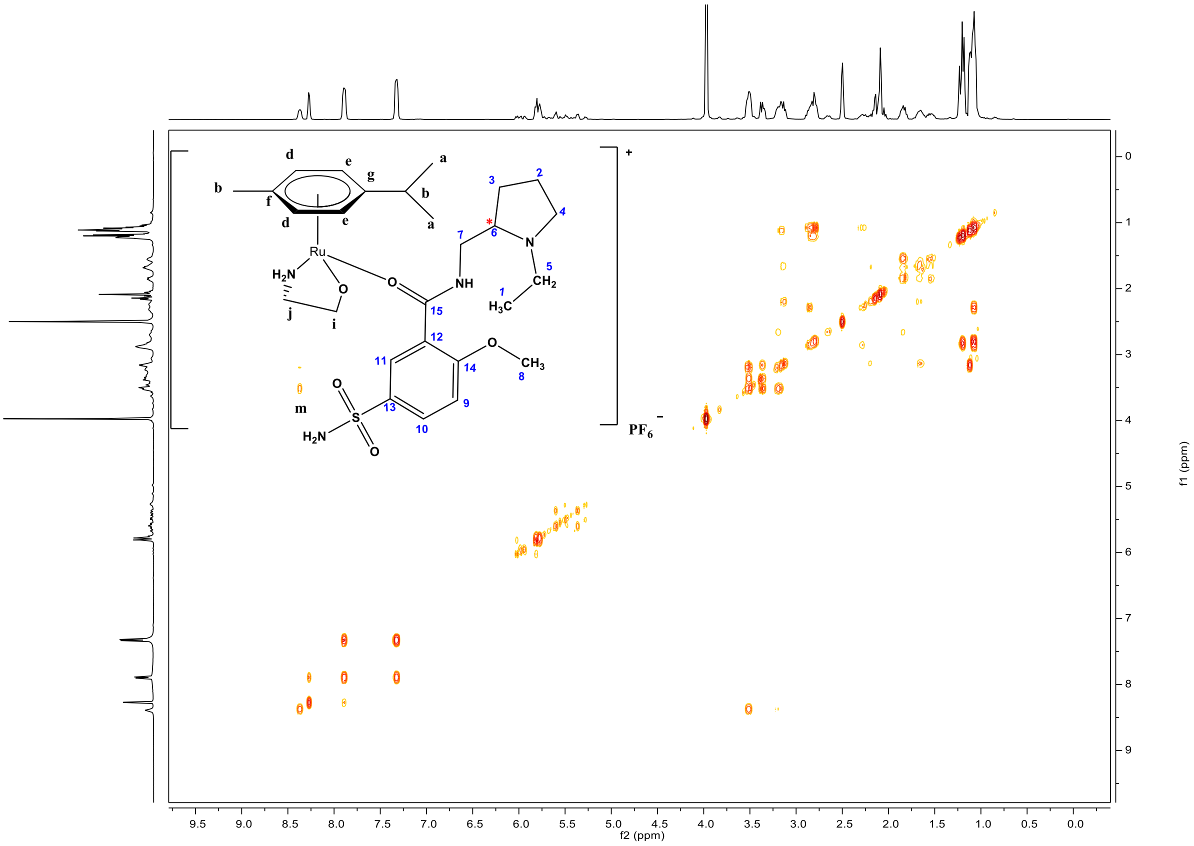


**Figure S22**. COSY NMR (500 MHz, , DMSO-*d6*) of Complex **3a**

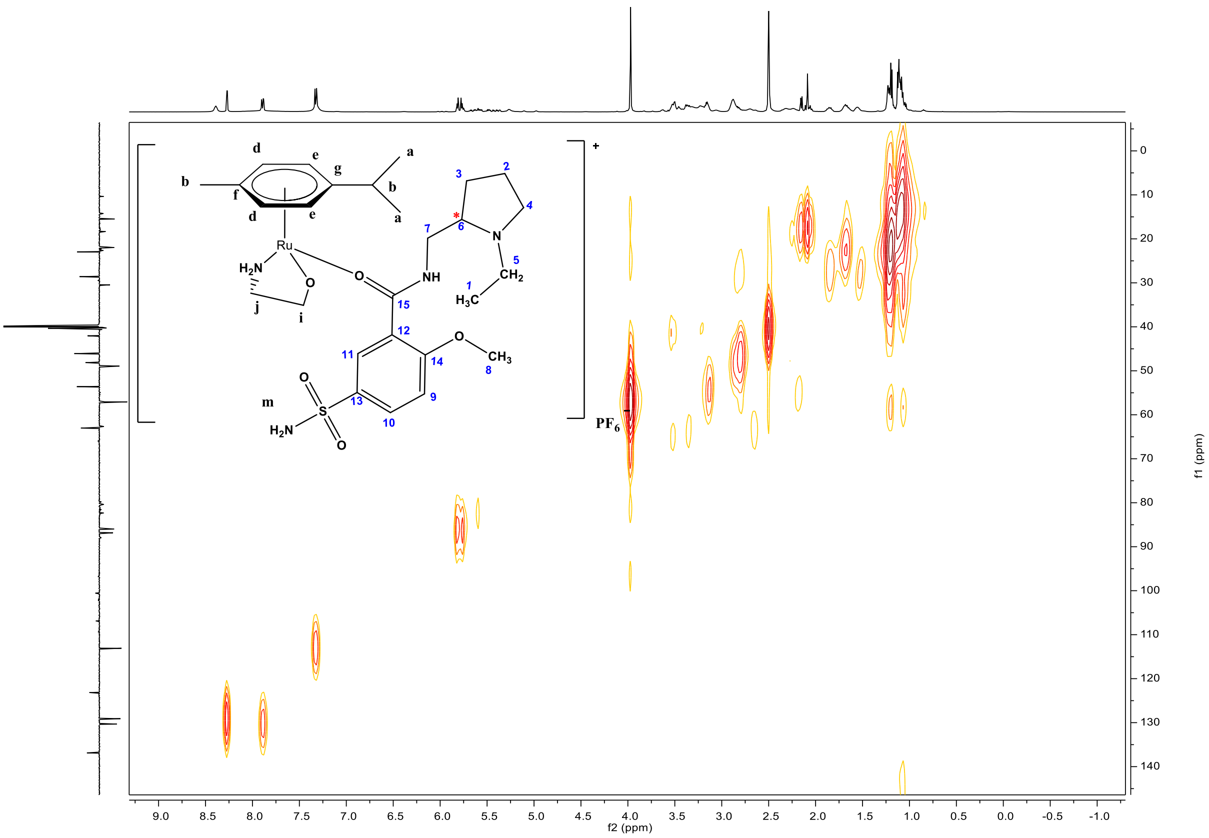

**Figure S23**. HSQC NMR (500 MHz, , DMSO-*d6*) of Complex **3a**

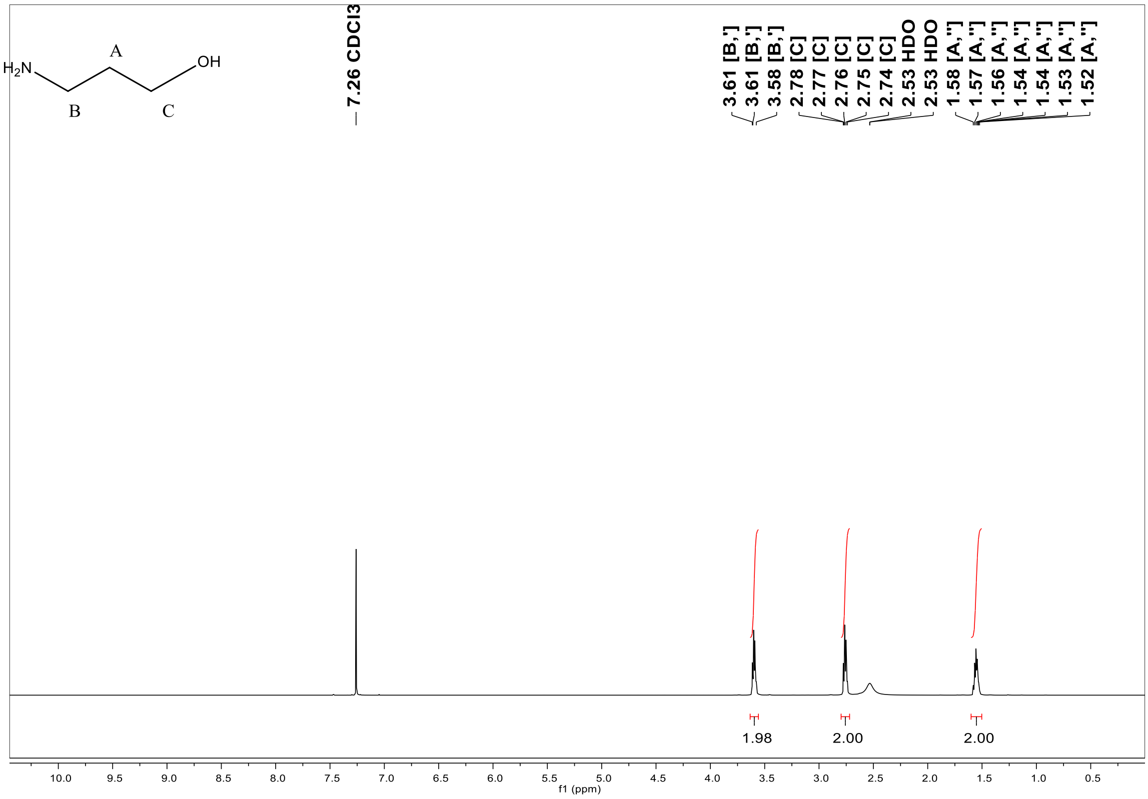


**Figure S24**. $^{1}$H NMR (500 MHz, Chloroform-*d*) of (L4)

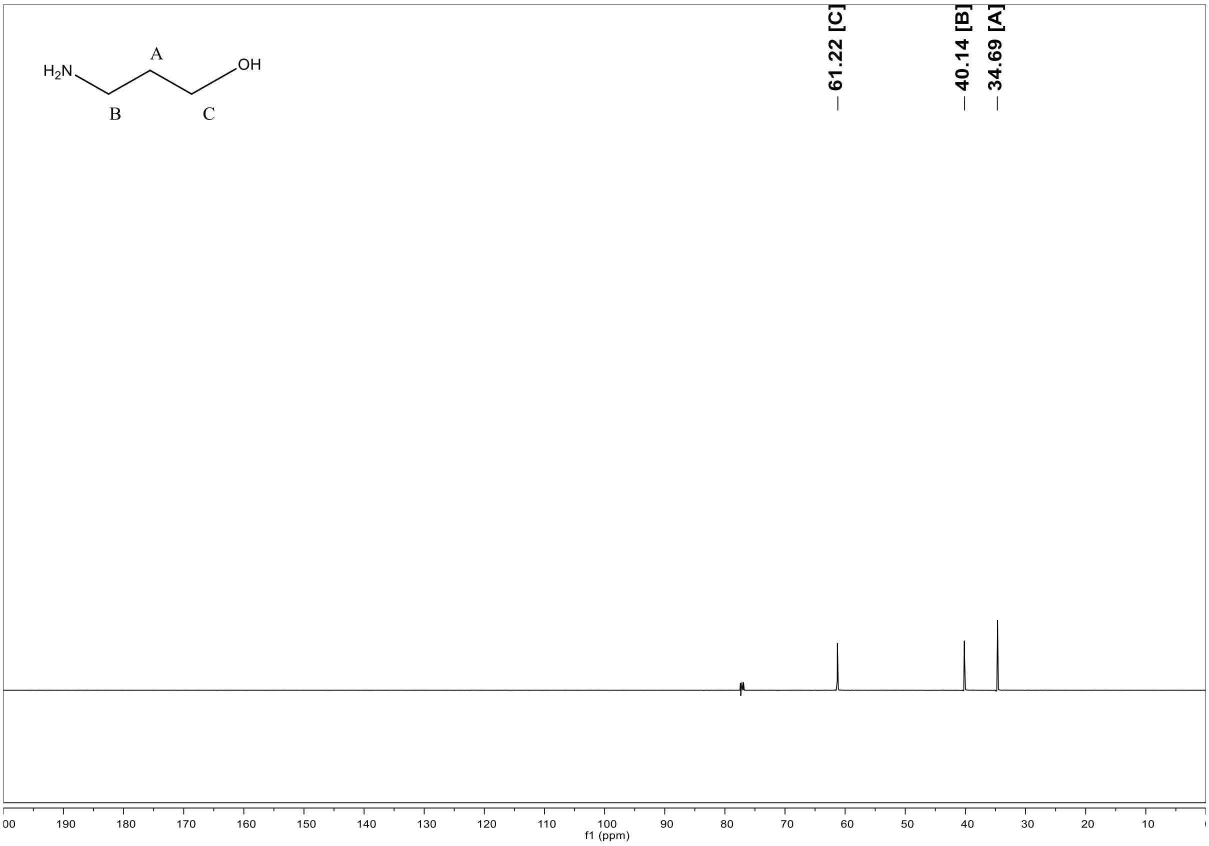


**Figure S25**. $^{13}C$ NMR (126 MHz, Chloroform-*d*) of (L4)

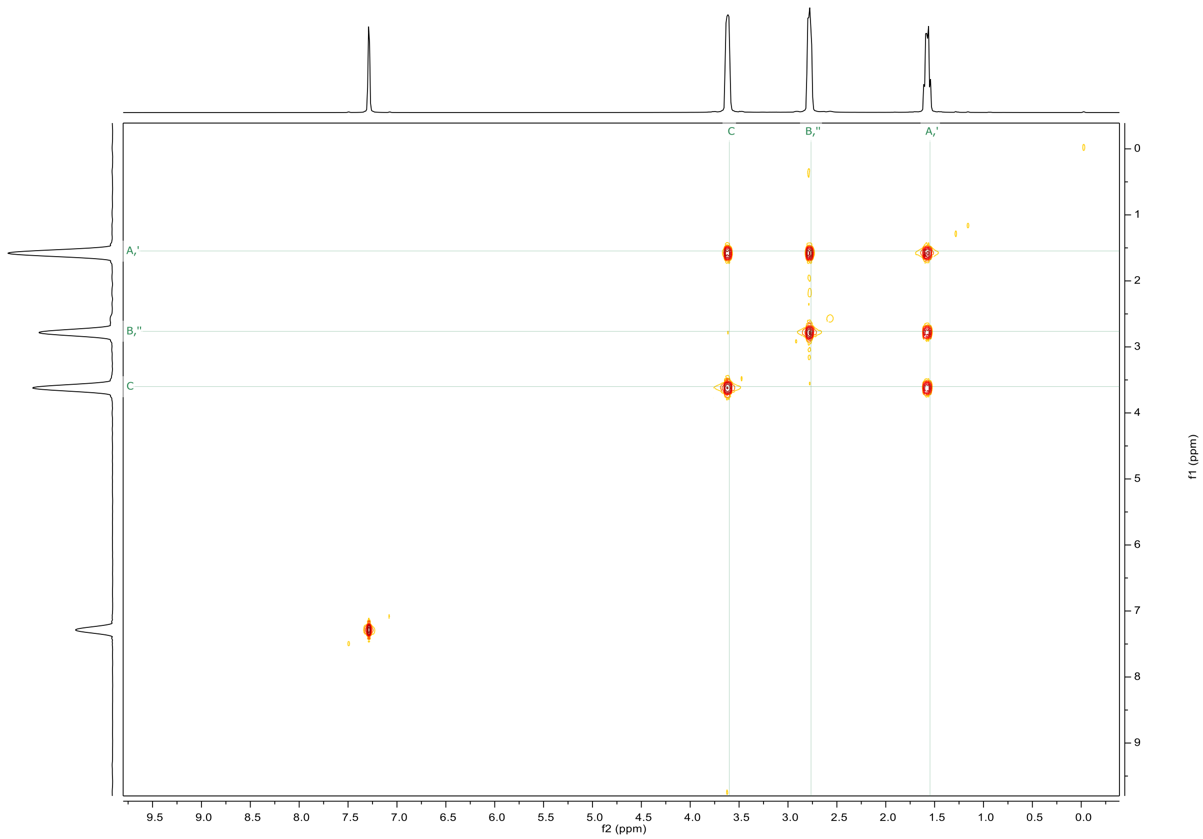


**Figure S26**. COSY NMR (500 MHz, Chloroform-*d*) of (L4)

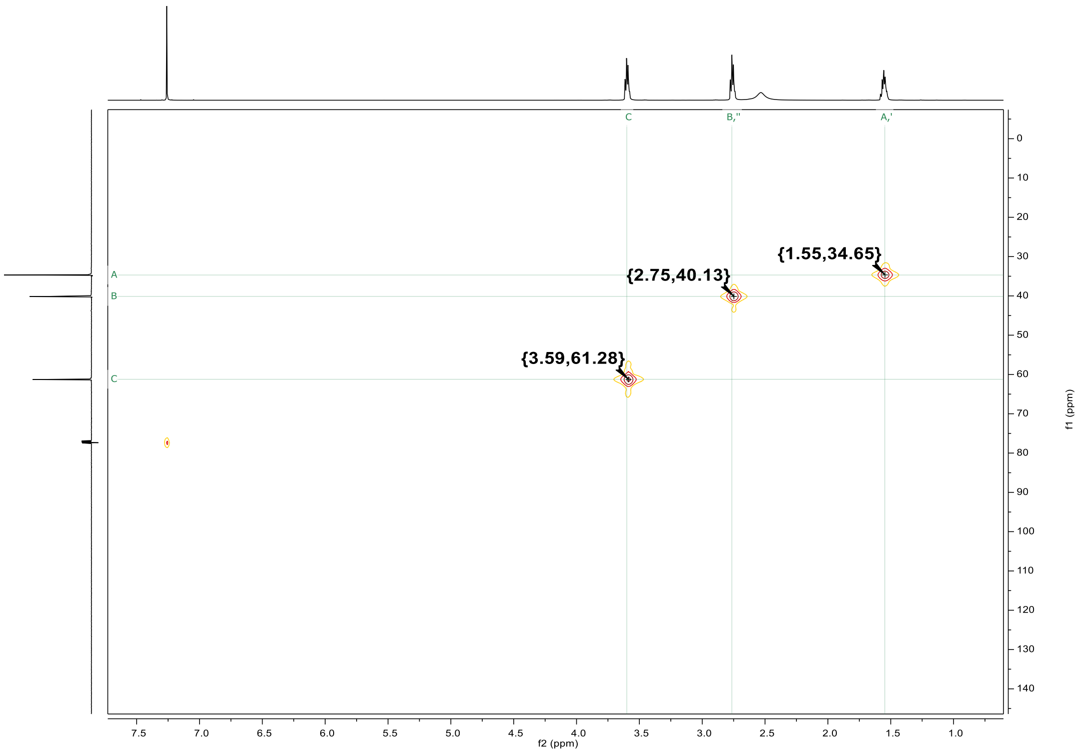


**Figure S27**. HMQC NMR (126 MHz, Chloroform-*d*) of (L4)

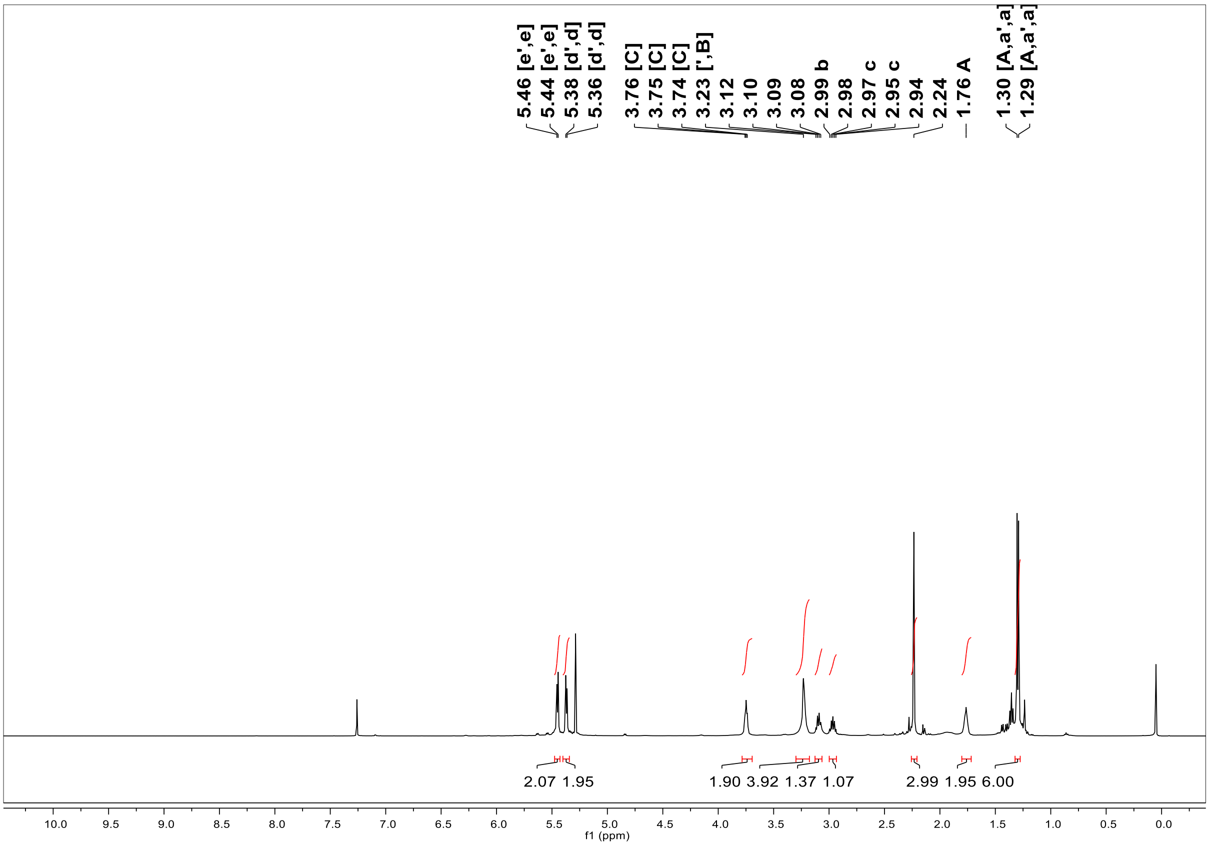


**Figure S28**. $^{1}$H NMR (500 MHz, Chloroform-*d*) of complex **4**

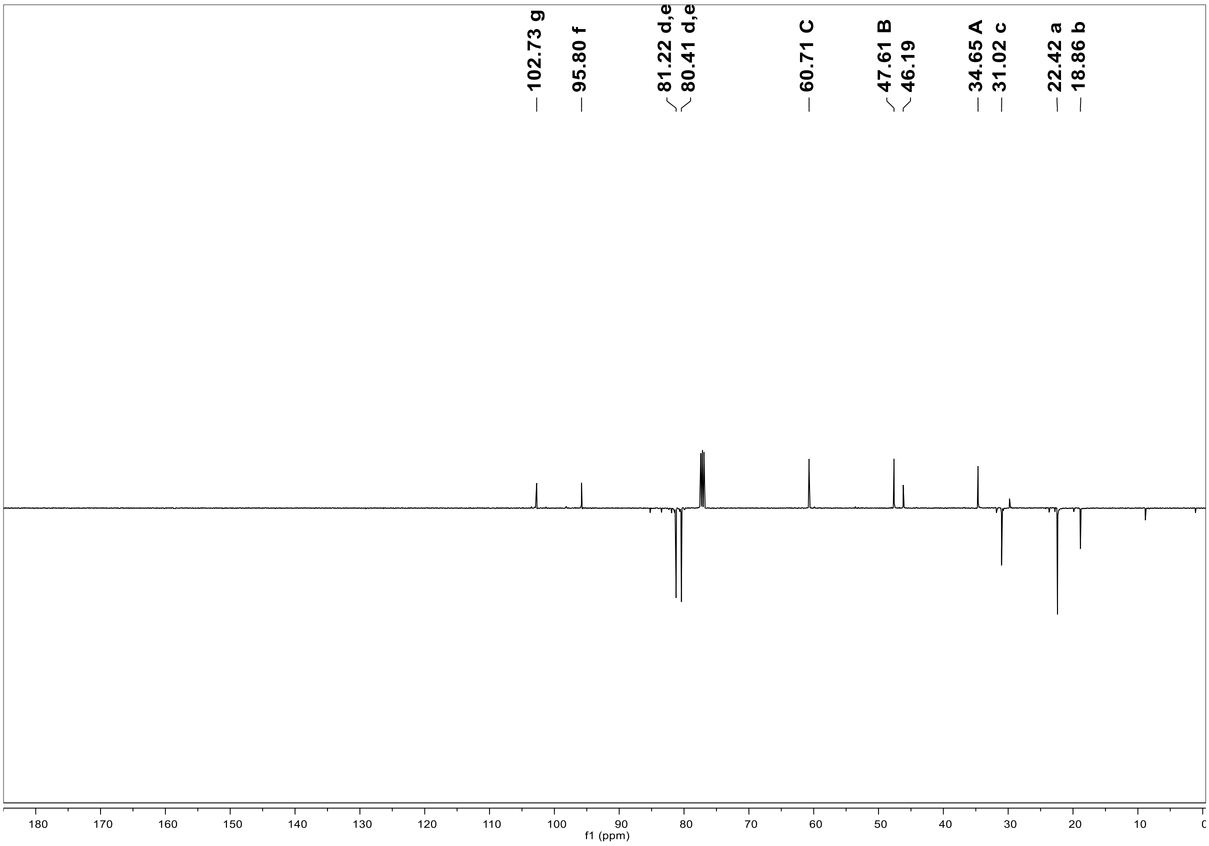


**Figure S29**. $^{13}$C NMR (126 MHz, Chloroform-*d*) of complex **4**

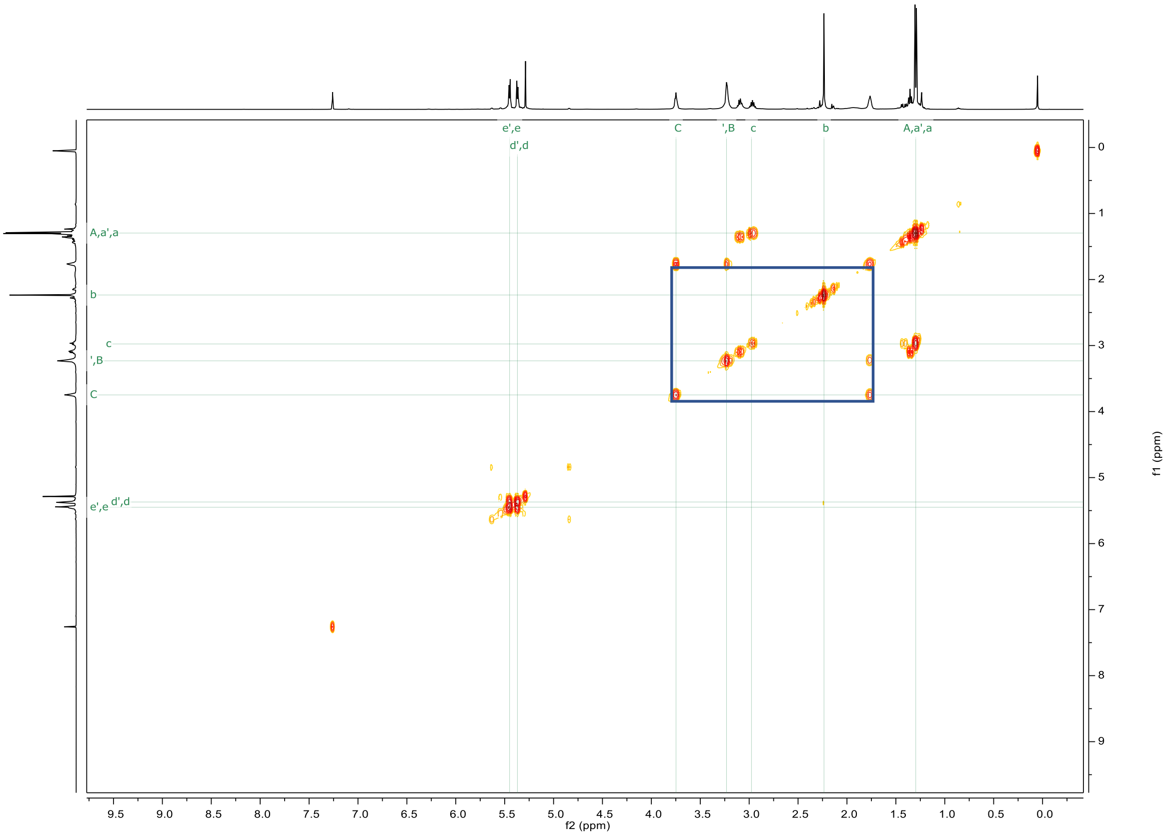


**Figure S30**. COSY (300 MHz, Chloroform-*d*) of complex **4**

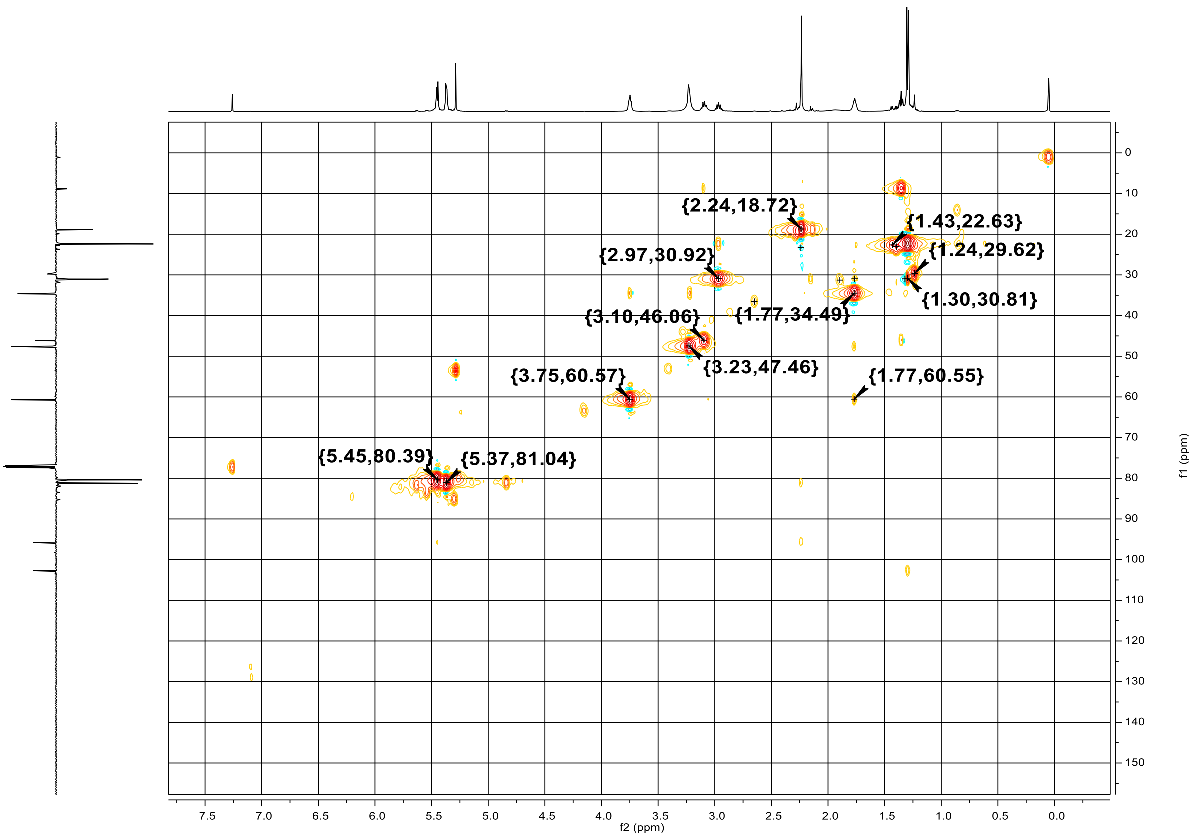


**Figure S31**. HMQC (126 MHz, Chloroform-*d*) of complex **4**

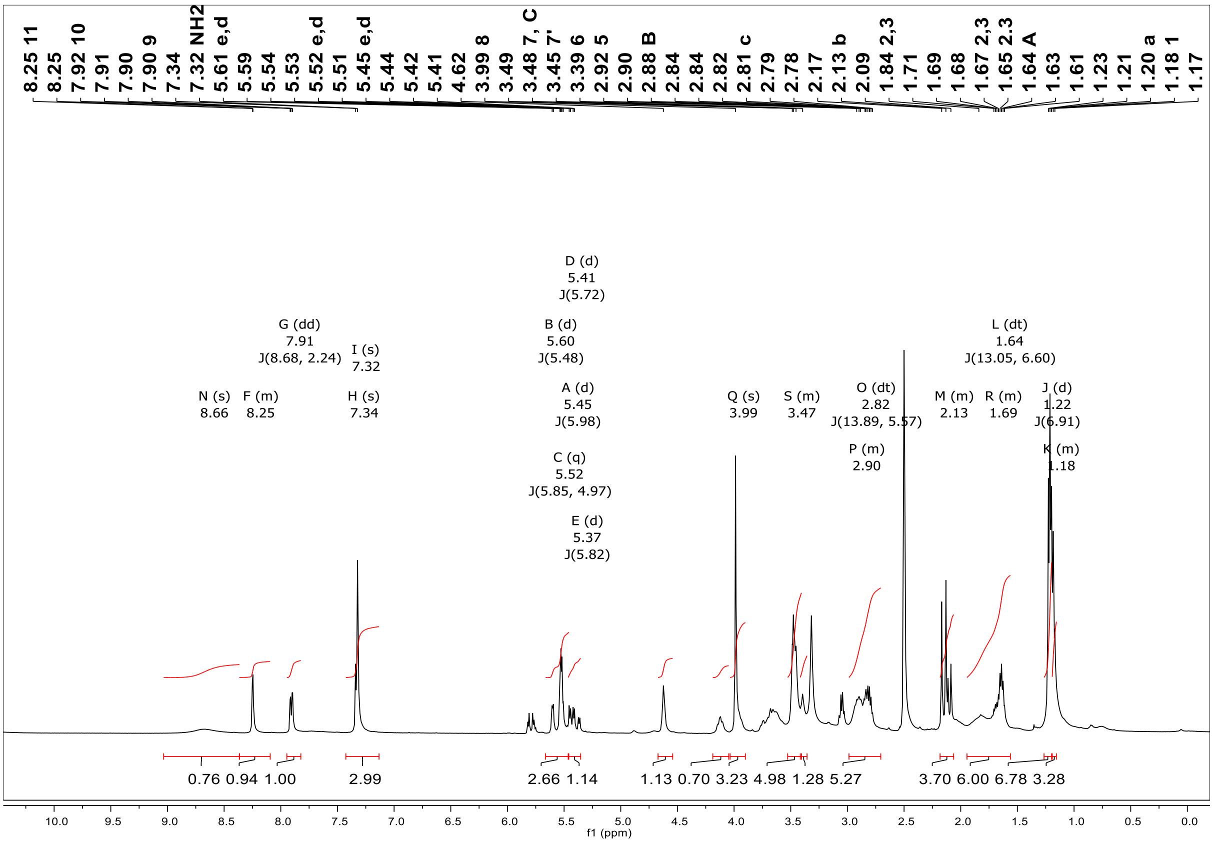


**Figure S32**. $^{1}$H NMR (126 MHz, Chloroform-*d*) of complex **4a**

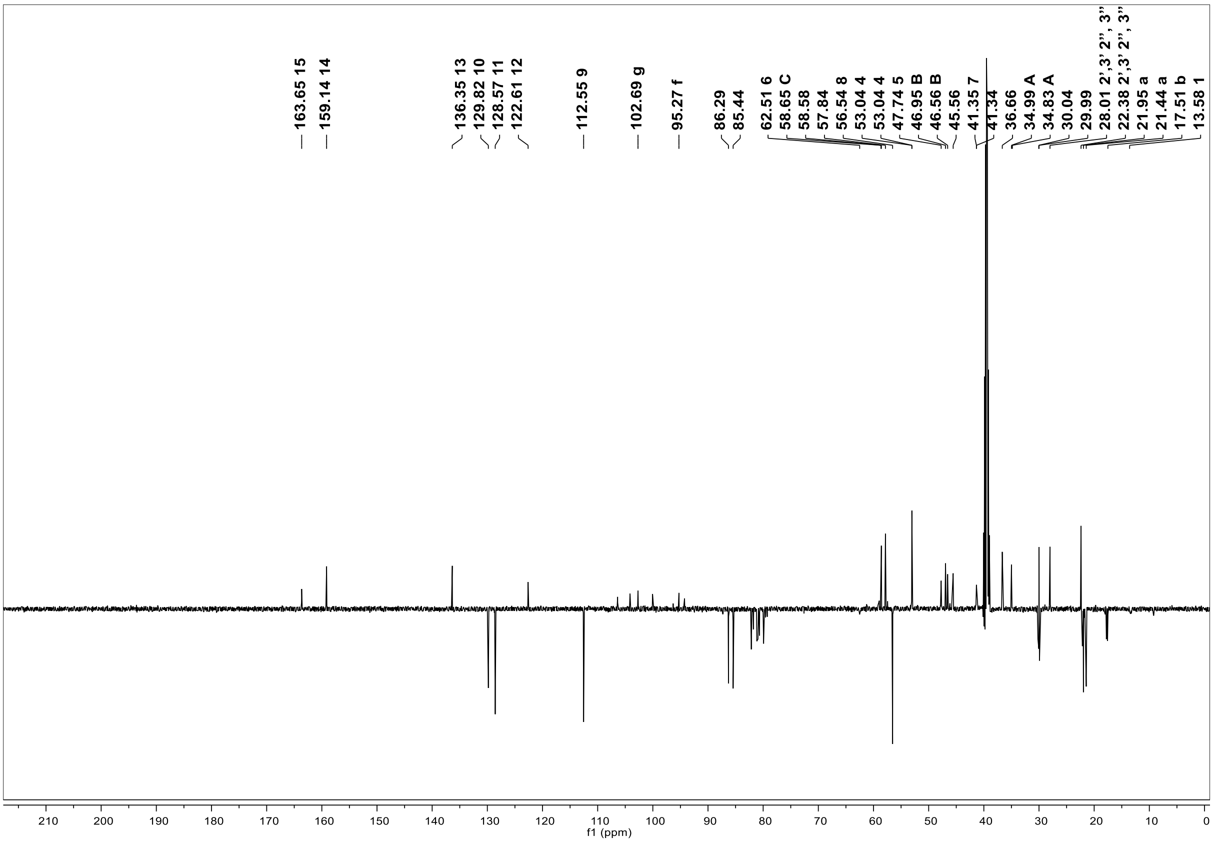


**Figure S33**. $^{13}C$ NMR (126 MHz, Chloroform-*d*) of complex **4a**

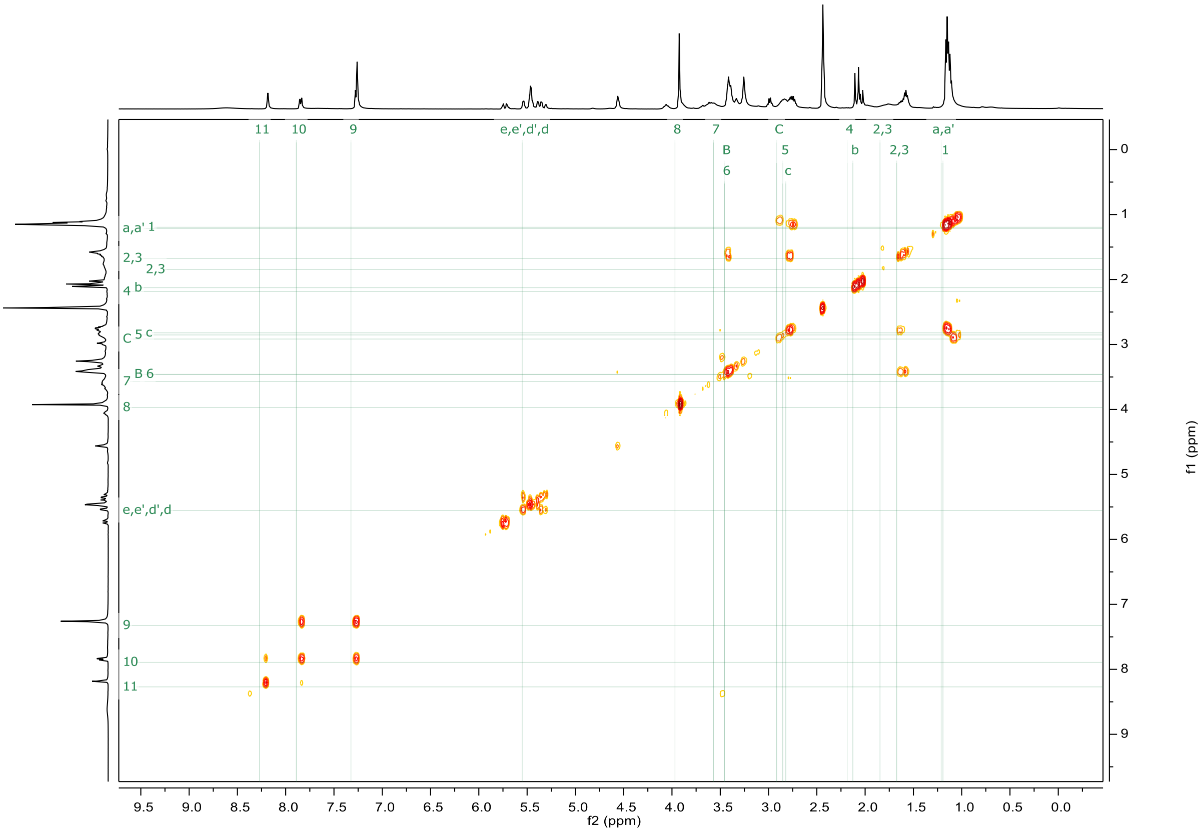


**Figure S34**. COSY (300 MHz, Chloroform-*d*) of complex **4a**

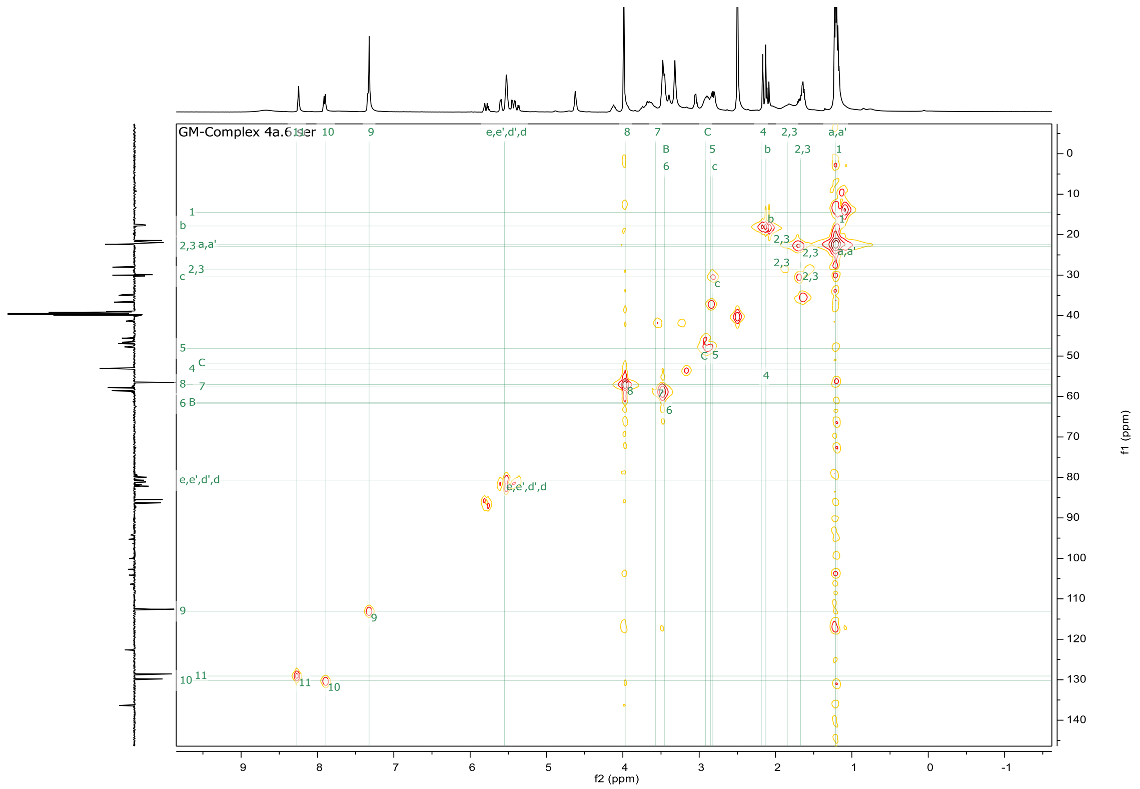

**Figure S35**. HMQC (126 MHz, Chloroform-*d*) of complex **4a**

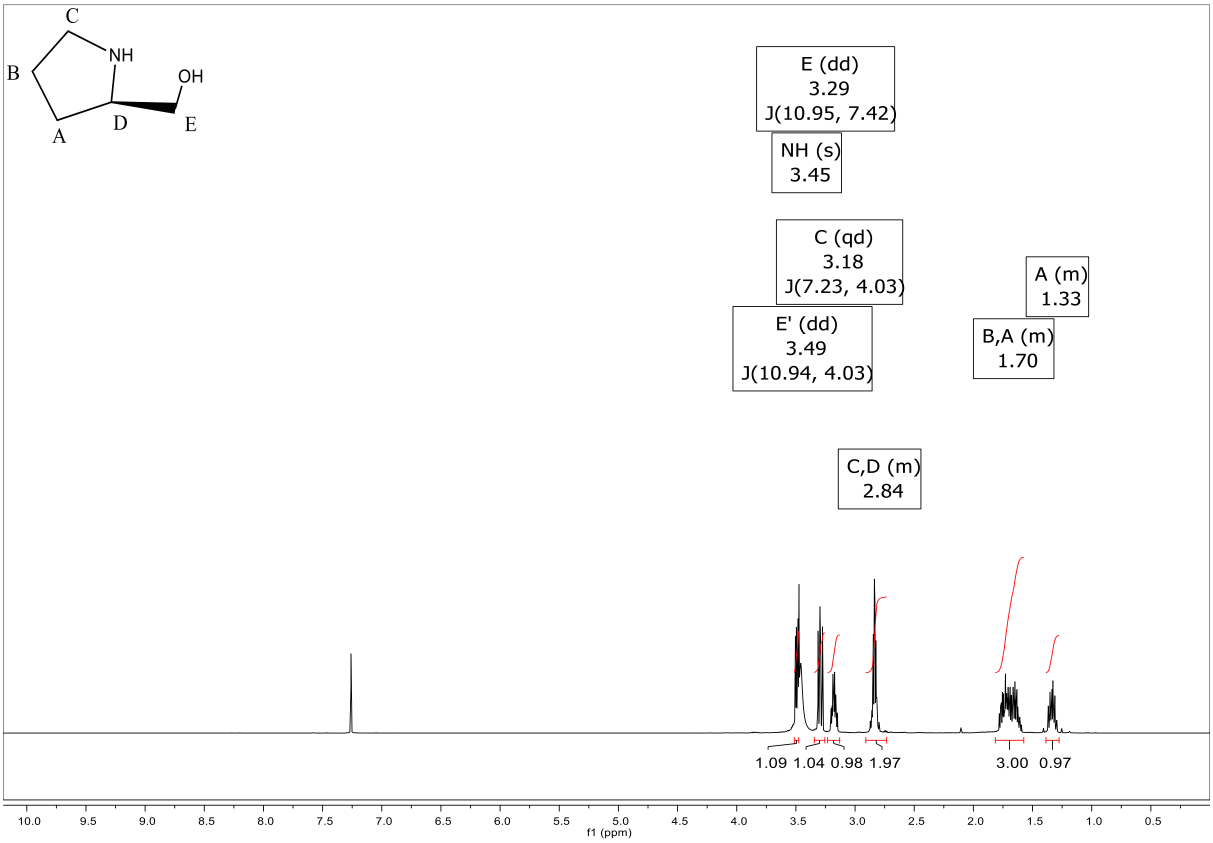

**Figure S36**. $^{1}$H NMR (500 MHz, Chloroform-*d*) of L5

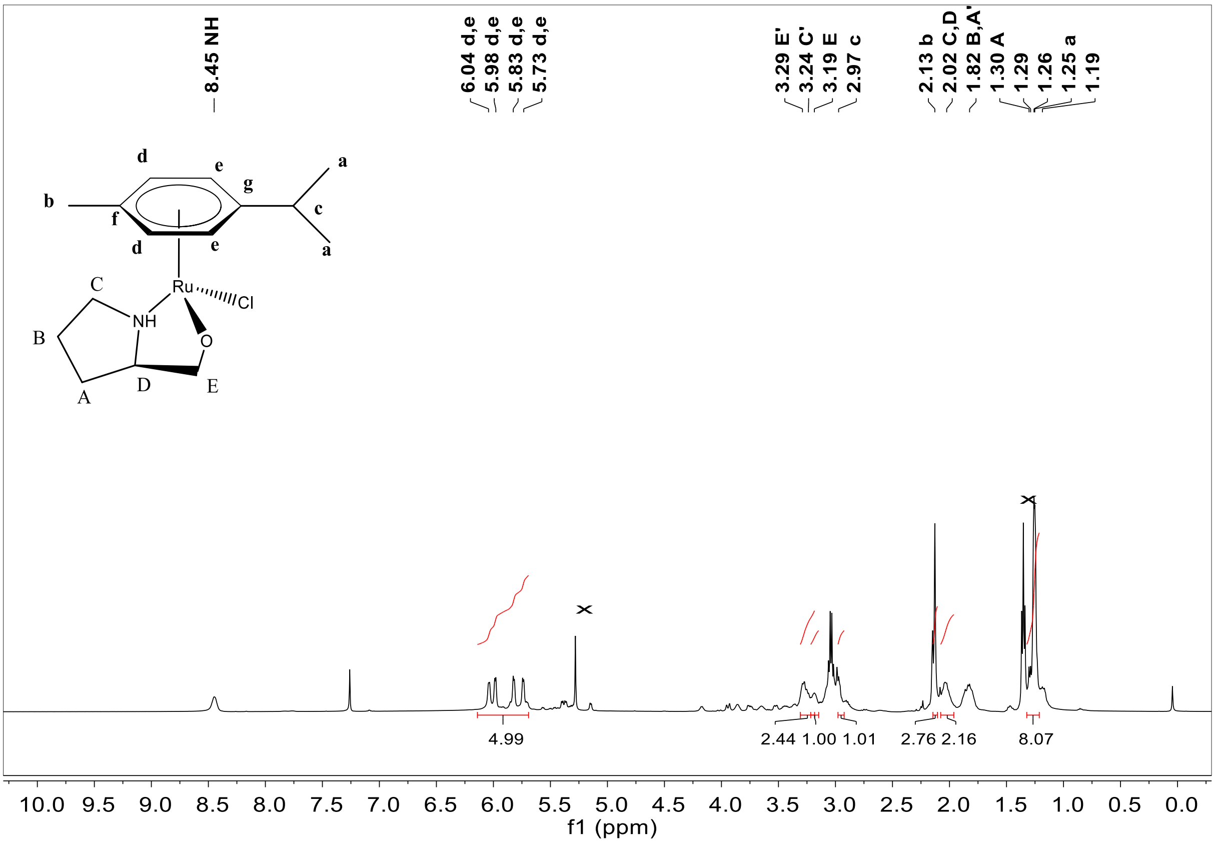


**Figure S37**. $^{1}$H NMR (500 MHz, Chloroform-*d*) of Complex **5**

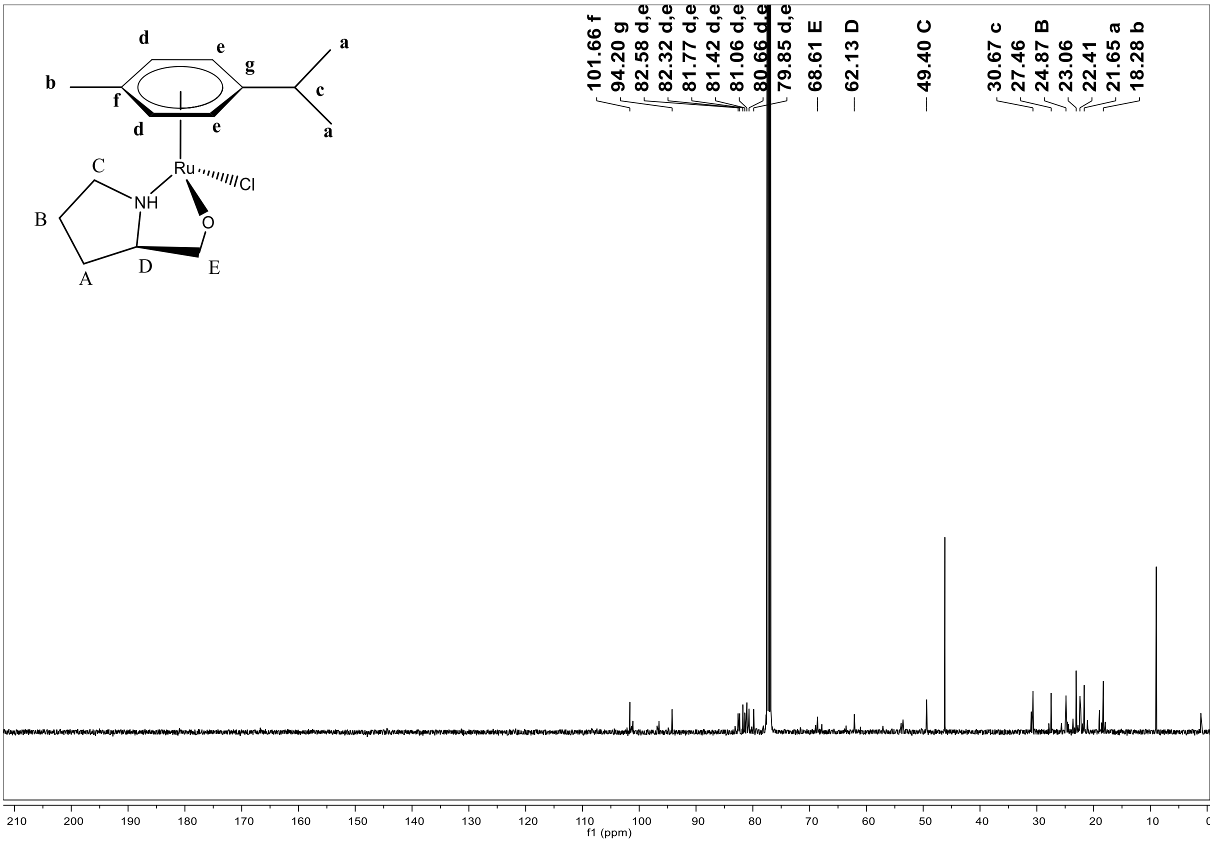


**Figure S38**. $^{13}$C NMR (126 MHz, Chloroform-*d*) of complex **5**

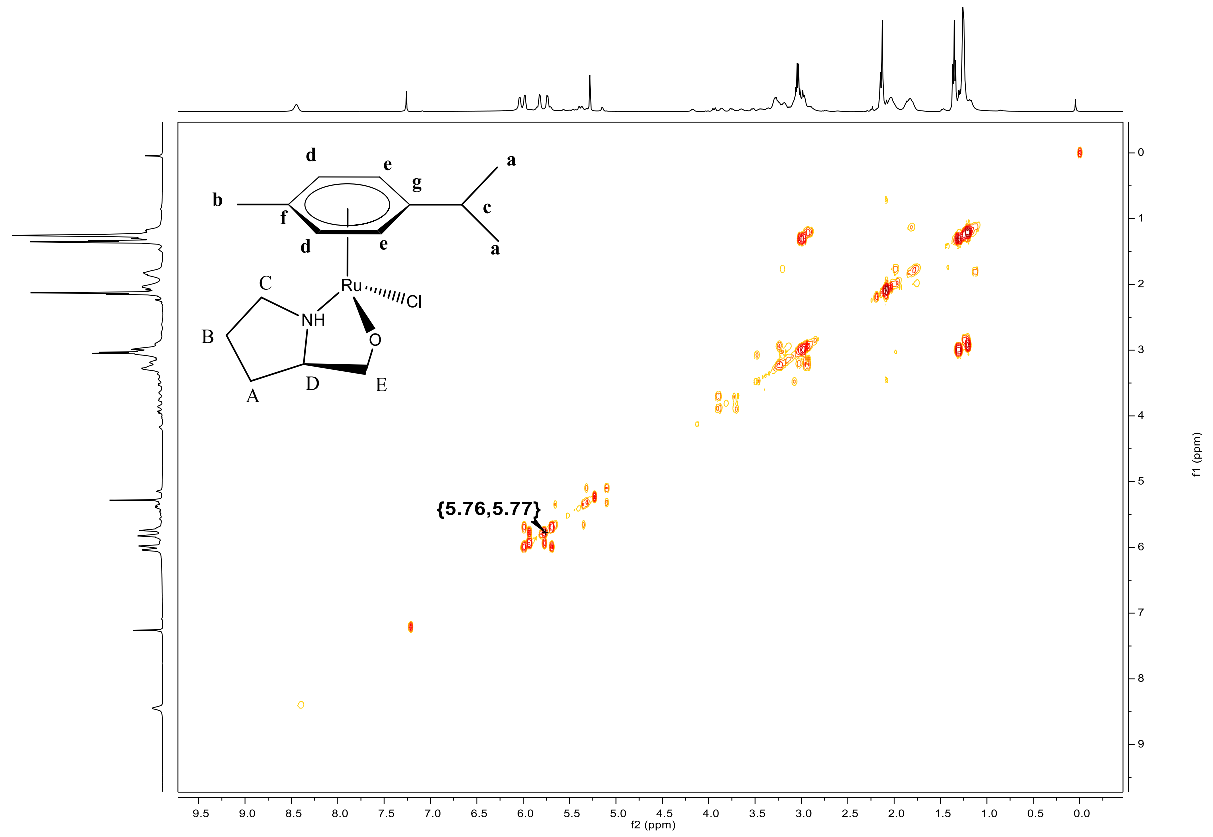


**Figure S39**. COSY NMR (500 MHz, Chloroform-*d*) of complex **5**

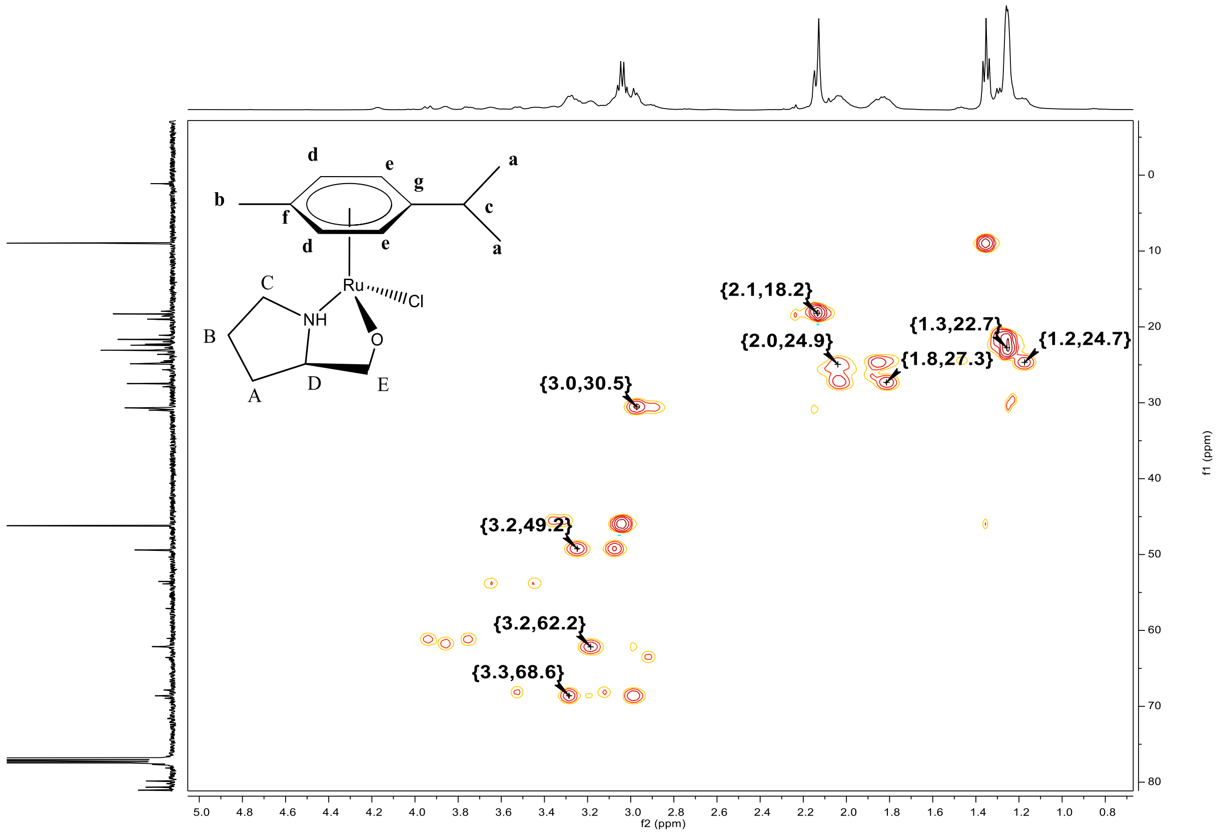


**Figure S40**. HMQC NMR (126 MHz, Chloroform-*d*) of complex **5**

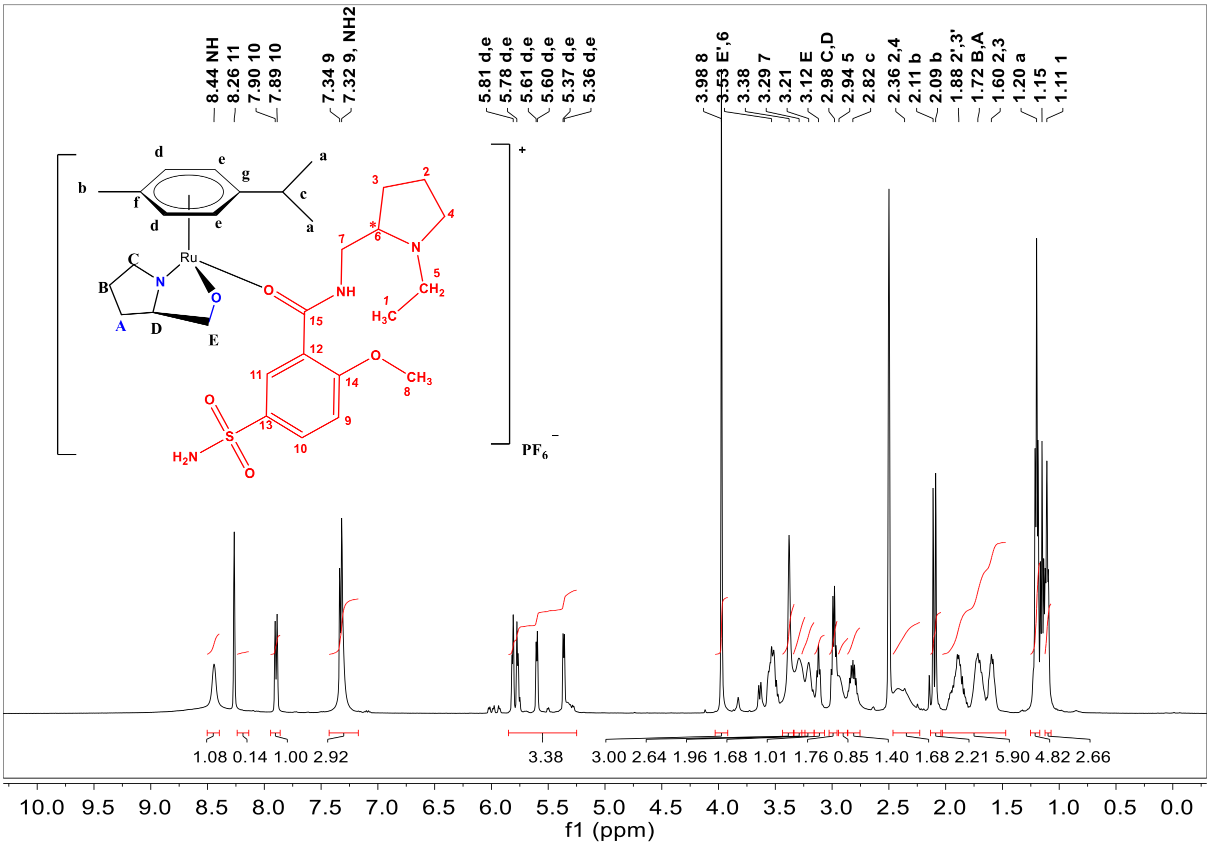

**Figure S41**. $^{1}$H NMR (126 MHz, Chloroform-*d*) of complex **5a**

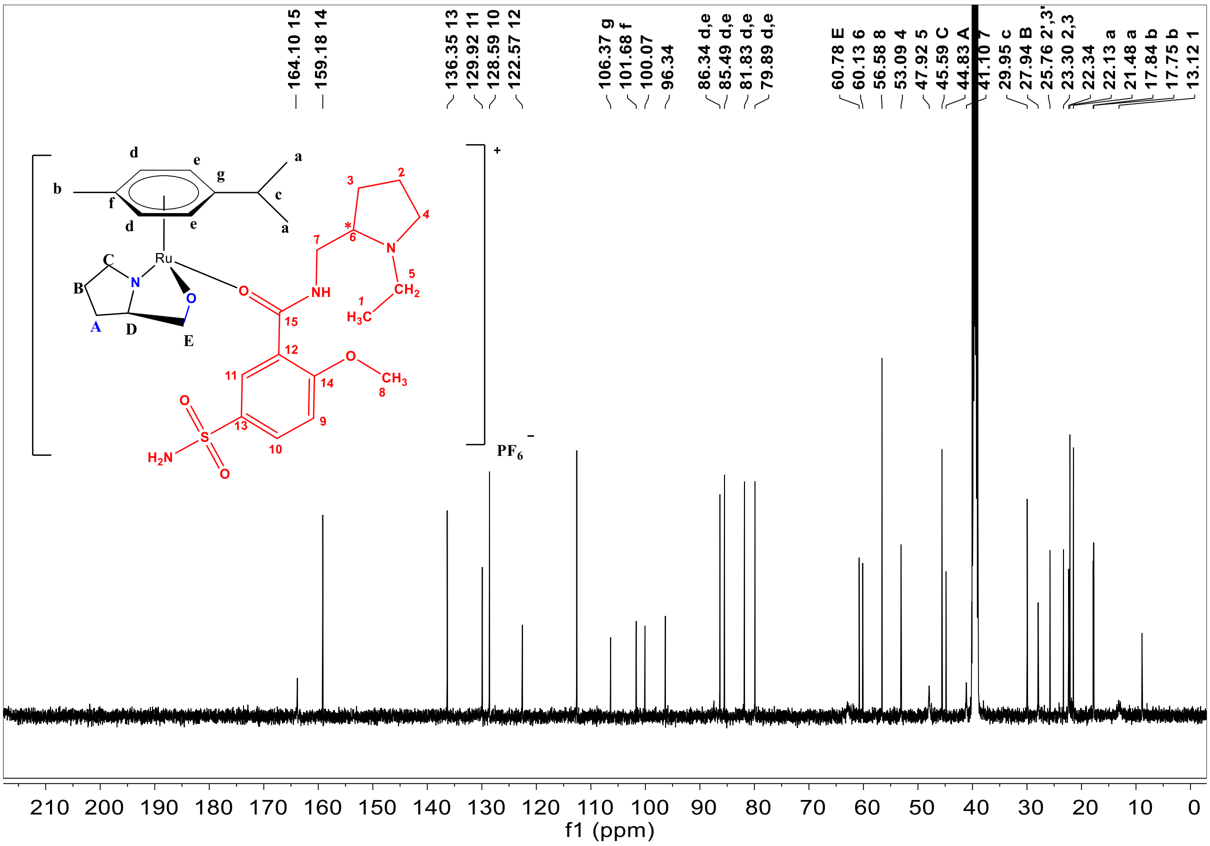

**Figure S42**. $^{13}$C NMR (126 MHz, Chloroform-*d*) of complex **5a**

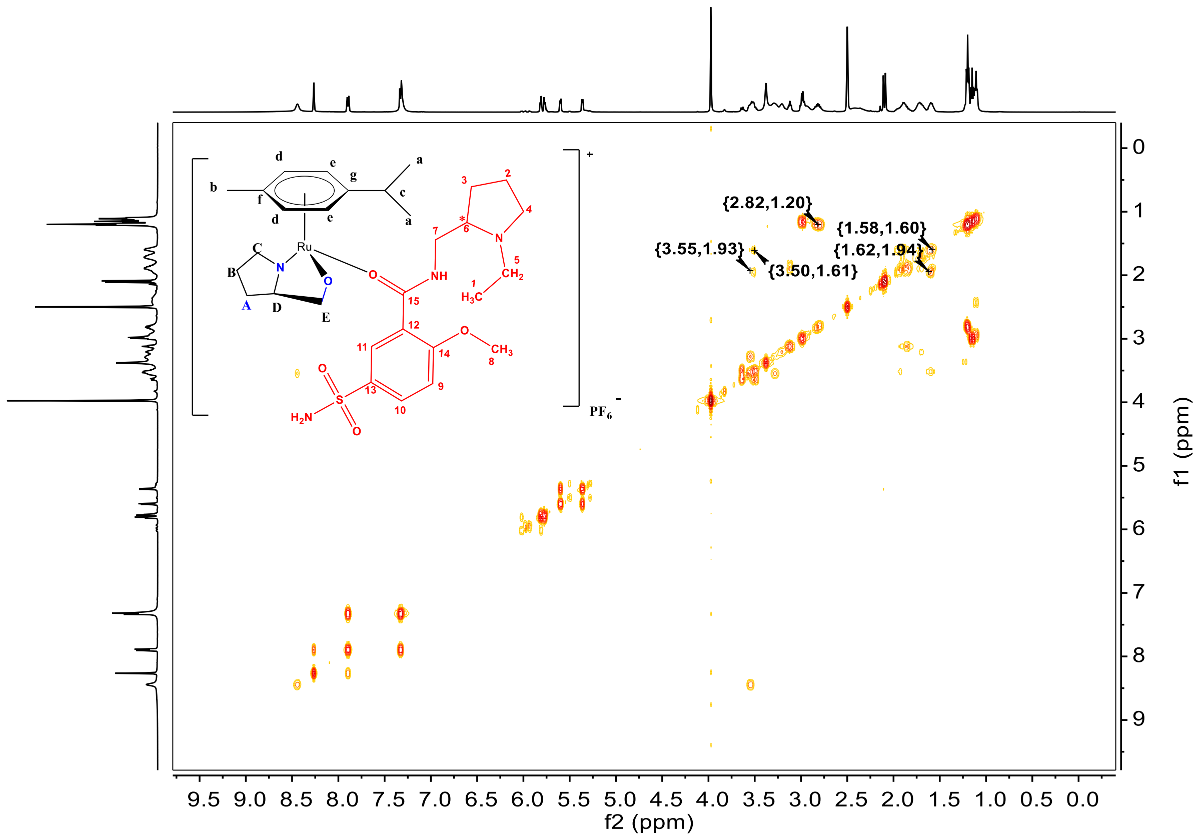

**Figure S43**. COSY (300 MHz, Chloroform-*d*) of complex **5a**

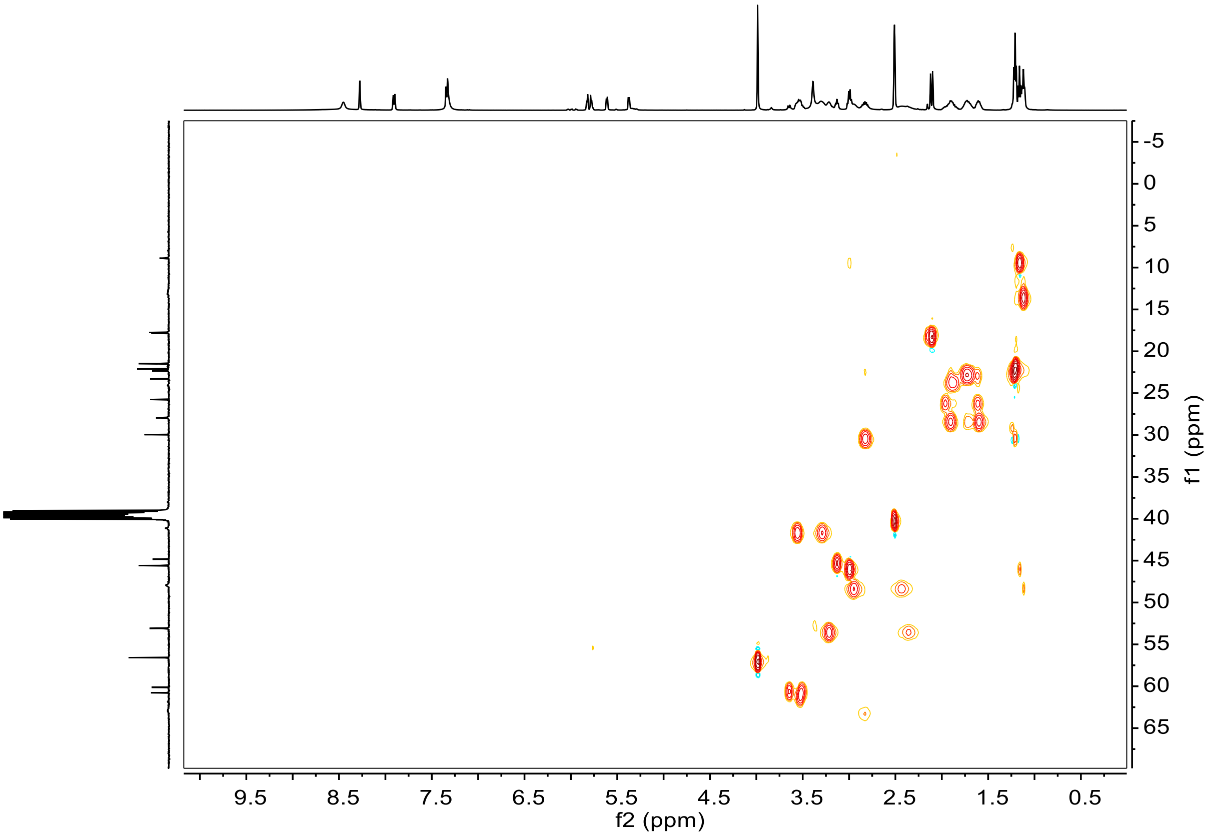

**Figure S44**. HMQC (126 MHz, Chloroform-*d*) of complex **5a**

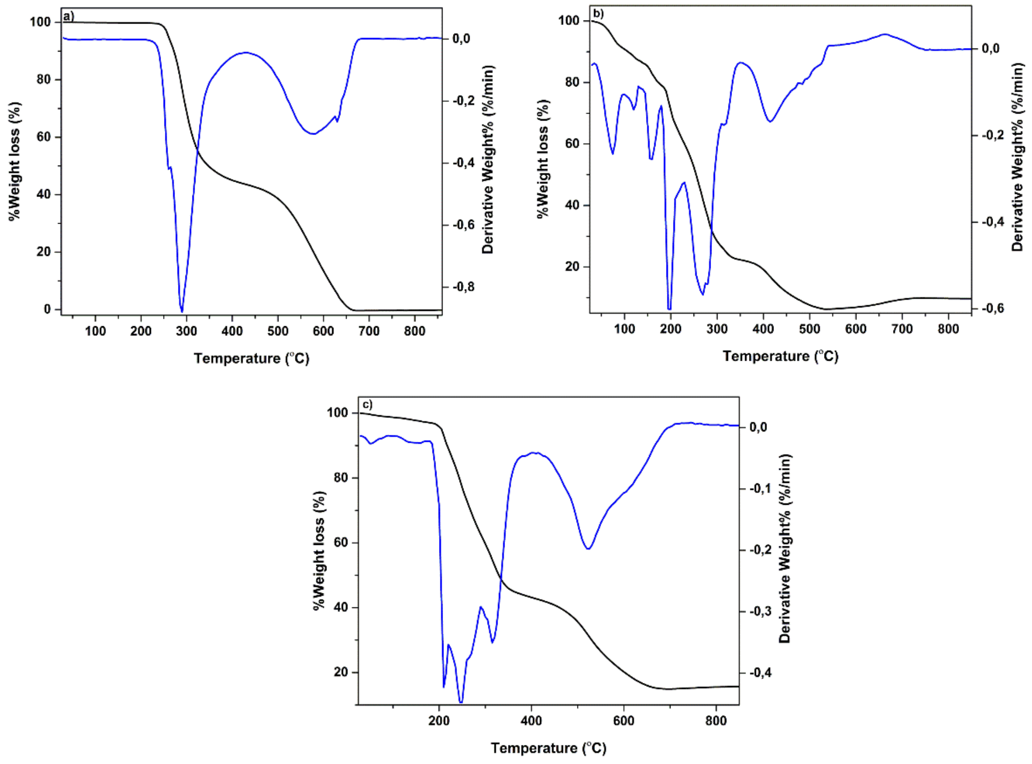


**Figure S45**. TGA thermograms of a) Sulpiride, b) Complex **1**) and c) Complex **1a**.

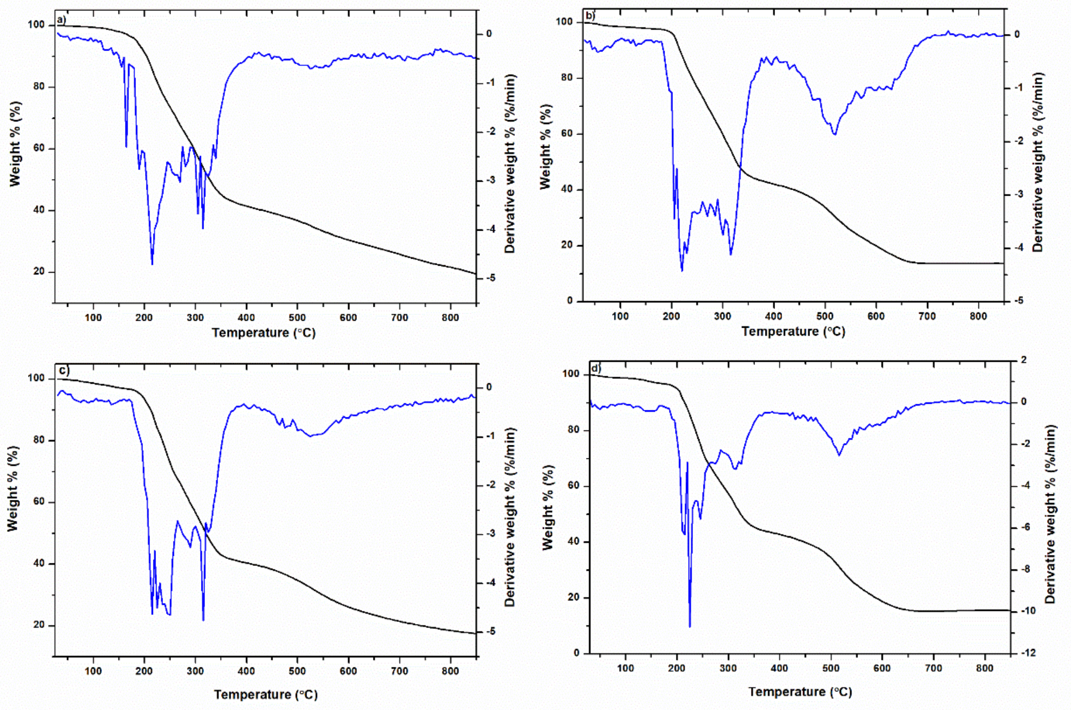


**Figure S46**. TGA thermograms of a) Complex **2a**, b) Complex **3a**, c) Complex **4a** and d) Complex **5a**

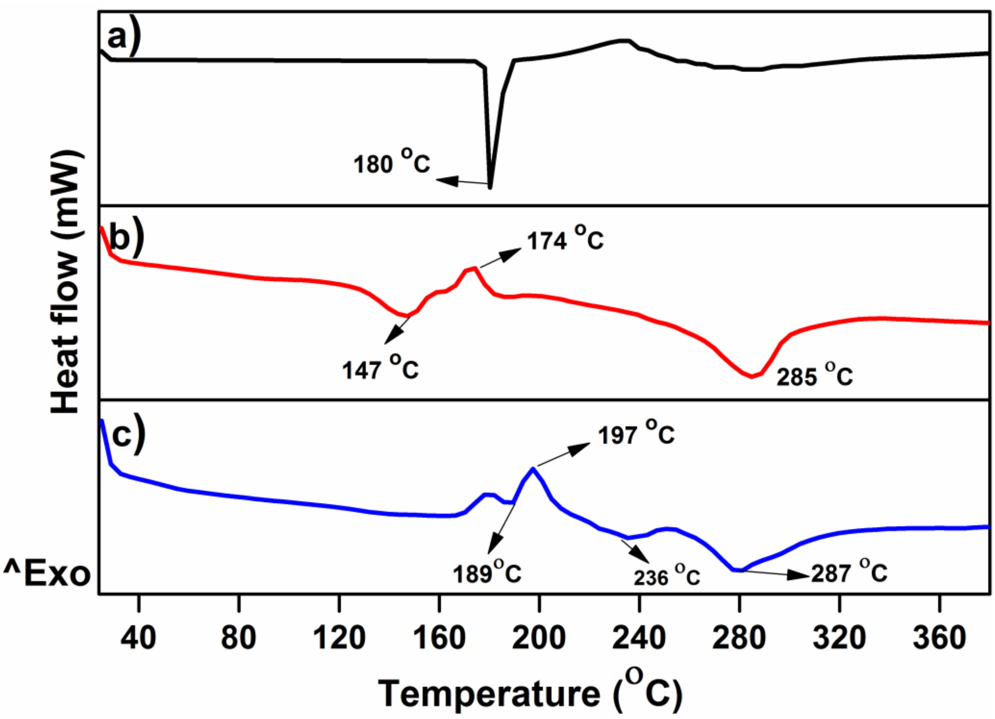


**Figure S47**. DSC curves of a) Sulpiride, b) Complex **1** and c) Complex **1a**)

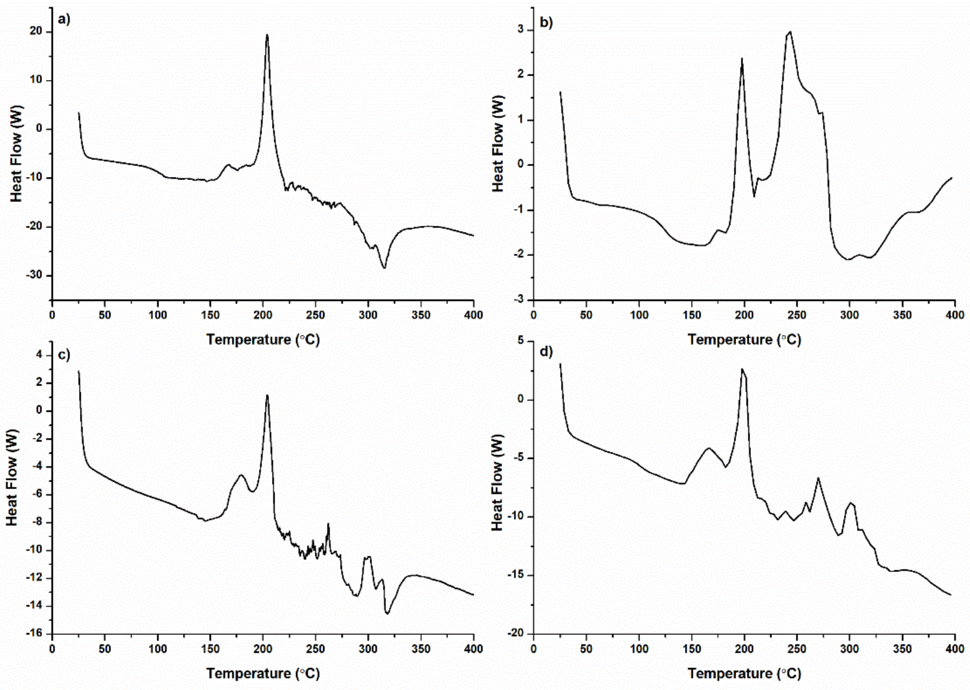


**Figure S48**. DSC curves of a) Complex **2a**, b) Complex **3a**, c) Complex **4a**, d) Complex **5a**

Sulpiride UV-Vis Calibration report

288 nm

| | |
|---|---|
| Std1.Replicate1 (A) | 0.1207 |
| Std1.Replicate2 (A) | 0.1209 |
| Std1.Replicate3 (A) | 0.1208 |
| Std1.Replicate4 (A) | 0.1208 |
| Std2.Replicate1 (A) | 0.2532 |
| Std2.Replicate2 (A) | 0.2531 |
| Std2.Replicate3 (A) | 0.2534 |
| Std2.Replicate4 (A) | 0.2532 |
| Std 3.Replicate1 (A) | 0.3883 |
| Std 3.Replicate2 (A) | 0.3882 |
| Std 3.Replicate3 (A) | 0.3881 |
| Std 3.Replicate4 (A) | 0.3885 |
| Std 4.Replicate1 (A) | 0.5391 |
| Std 4.Replicate2 (A) | 0.5392 |
| Std 4.Replicate3 (A) | 0.5392 |
| Std 4.Replicate4 (A) | 0.5392 |
| Std 5.Replicate1 (A) | 0.6623 |
| Std 5.Replicate2 (A) | 0.6618 |
| Std 5.Replicate3 (A) | 0.6618 |
| Std 5.Replicate4 (A) | 0.6617 |

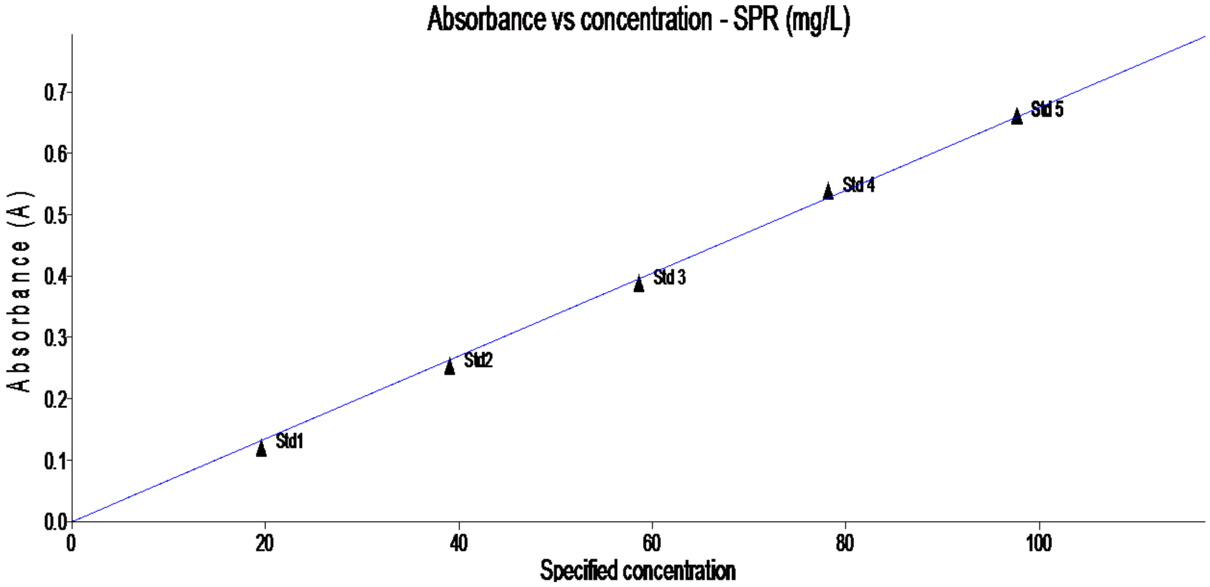


**Figure S47**. Sulpiride calibration curve

CALIBRATION REPORT

Calibration Time: Tuesday, December 02, 2014 4:37 PM South Africa Standard Time
User Full Name : Analyst

Component Name: SPR
Component Units: mg/L

Calibration: Calibration Curve - Linear (y=a1x+a0)
Baseline Correction: None

Settings (nm): Position:288.00

Force through Zero: Yes

Calibration Coefficients :
a0 = 0.000000
a1 = 0.006754

Specified Correlation Coefficient: 0.980000
Calculated Correlation Coefficient: 0.998854

| Index | StandardID | Specified | Calculated | Residual |
|---|---|---|---|---|
| 1 | Std1 | 19.5400 | 17.8796 | 1.6604 |
| 2 | Std1 | 19.5400 | 17.9035 | 1.6365 |
| 3 | Std1 | 19.5400 | 17.8816 | 1.6584 |
| 4 | Std1 | 19.5400 | 17.8879 | 1.6521 |
| 5 | Std2 | 39.0800 | 37.4984 | 1.5816 |
| 6 | Std2 | 39.0800 | 37.4815 | 1.5985 |
| 7 | Std2 | 39.0800 | 37.5253 | 1.5547 |
| 8 | Std2 | 39.0800 | 37.4843 | 1.5957 |
| 9 | Std 3 | 58.6200 | 57.4918 | 1.1282 |
| 10 | Std 3 | 58.6200 | 57.4841 | 1.1359 |
| 11 | Std 3 | 58.6200 | 57.4591 | 1.1609 |
| 12 | Std 3 | 58.6200 | 57.5227 | 1.0973 |
| 13 | Std 4 | 78.1600 | 79.8186 | -1.6586 |
| 14 | Std 4 | 78.1600 | 79.8350 | -1.6750 |
| 15 | Std 4 | 78.1600 | 79.8350 | -1.6750 |
| 16 | Std 4 | 78.1600 | 79.8459 | -1.6859 |
| 17 | Std 5 | 97.7000 | 98.0626 | -0.3626 |
| 18 | Std 5 | 97.7000 | 97.9902 | -0.2902 |
| 19 | Std 5 | 97.7000 | 97.9866 | -0.2866 |
| 20 | Std 5 | 97.7000 | 97.9721 | -0.2721 |
